# Metallo-β-Lactamase Inhibitor Phosphonamidate Monoesters

**DOI:** 10.1021/acsomega.1c06527

**Published:** 2022-01-25

**Authors:** Katarzyna Palica, Manuela Vorácová, Susann Skagseth, Anna Andersson Rasmussen, Lisa Allander, Madlen Hubert, Linus Sandegren, Hanna-Kirstirep Schrøder Leiros, Hanna Andersson, Máté Erdélyi

**Affiliations:** † Department of Chemistry—BMC, Organic Chemistry, Uppsala University, Husargatan 3, 752 37 Uppsala, Sweden; ‡ The Norwegian Structural Biology Centre (NorStruct), Department of Chemistry, Faculty of Science and Technology, UiT The Arctic University of Norway, N-9037 Tromsø, Norway; § Department of Medical Biochemistry and Microbiology—BMC, Uppsala University, Husargatan 3, 752 37 Uppsala, Sweden; ∥ Department of Pharmacy—BMC, Uppsala University, Husargatan 3, 752 37 Uppsala, Sweden

## Abstract

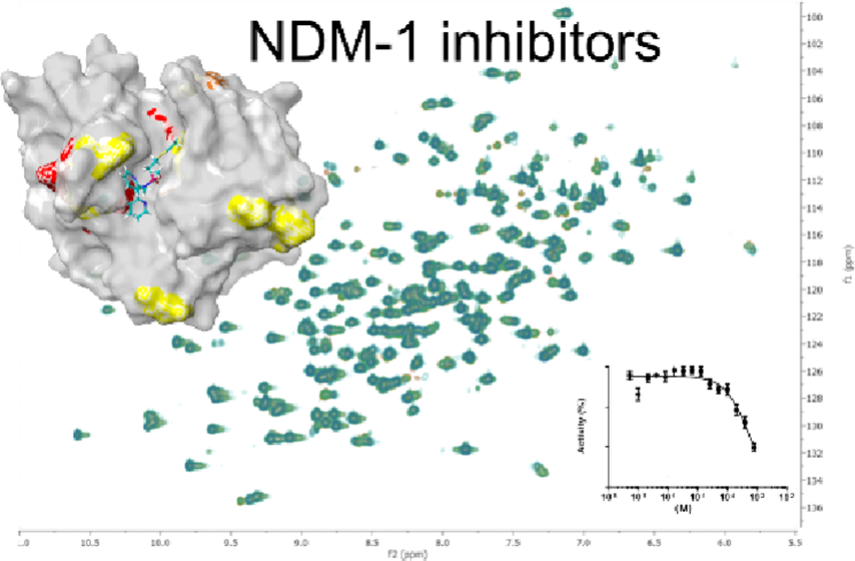

Being the second leading cause of death and the leading cause of disability-adjusted life years worldwide, infectious diseases remain—contrary to earlier predictions—a major consideration for the public health of the 21^st^ century. Resistance development of microbes to antimicrobial drugs constitutes a large part of this devastating problem. The most widely spread mechanism of bacterial resistance operates through the degradation of existing β-lactam antibiotics. Inhibition of metallo-β-lactamases is expected to allow the continued use of existing antibiotics, whose applicability is becoming ever more limited. Herein, we describe the synthesis, the metallo-β-lactamase inhibition activity, the cytotoxicity studies, and the NMR spectroscopic determination of the protein binding site of phosphonamidate monoesters. The expression of single- and double-labeled NDM-1 and its backbone NMR assignment are also disclosed, providing helpful information for future development of NDM-1 inhibitors. We show phosphonamidates to have the potential to become a new generation of antibiotic therapeutics to combat metallo-β-lactamase-resistant bacteria.

## Introduction

The deployment of antibiotics in the mid-twentieth century vastly decreased the mortality of infectious diseases.^1^ Most classes of antimicrobial agents in current use have been marketed over the 40 years following the discovery of penicillin.^2 ,3^ Those commercialized over the past decades have typically been associations or improvements of previously existing compounds. The rapidly growing antimicrobial resistance against the existing antibiotics is considered as one of the biggest public health challenges of the 21st century, whereas large pharmaceutical enterprises typically evade the antimicrobial resistance research area. No new antibiotics with a novel mode of action have lately reached the market;^4^ however, a handful of drug candidates targeting various antibiotic resistance mechanisms are currently in clinical trials,^5−7^ including, for example, the two efflux-bypassing drugs zoliflodacin and gepotidacin, which have reached phase three trials.^8^

β-Lactams are by far the most used antibiotics worldwide. This antibiotic class includes substance groups such as the penicillins, cephalosporins, monobactams, and carbapenems that share a β-lactam ring as their common structural feature. They act similarly by inactivating the penicillin-binding proteins that are essential for the formation of the bacterial cell wall. Among the antibacterial resistance mechanisms, β-lactamases are one of the most troublesome as they hydrolyze all existing types of β-lactams, including also carbapenems, our last-resort drugs.^9^ Based on their mechanism of action, β-lactamases are divided into serine-β-lactamases (SBLs) and metallo-β-lactamases (MBLs), of which the latter are characterized by large and complex structural variations within the group. Both groups of enzymes hydrolyze the β-lactam ring, resulting in the complete loss of antibiotic activity yet through different mechanisms.^10^ Besides developing new drugs acting through completely new mechanisms, an expected feasible strategy to combat bacterial resistance is the use of a β-lactamase inhibitor together with an existing β-lactam antibiotic, the former protecting the latter when used in combination therapy. SBL inhibitors for clinical use were introduced already in the 1980s, whereas no clinically approved inhibitors of MBLs have yet reached the market.

**Figure 1:**
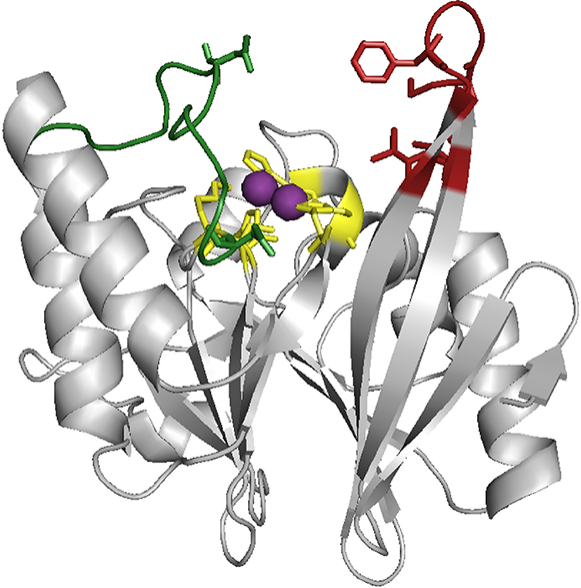
Secondary structure of NDM-1 with loop 3 (red), loop 10 (green), and the zinc (violet)-coordinating amino acids (yellow) being highlighted (PDB: 4hl2).

The New Delhi metallo-β-lactamase 1 (NDM-1) enzyme,^11−13^ first reported in 2008, is currently one of the major causes of concern. It is widespread and confers resistance to essentially all β-lactams. It has been reported to become common in *Escherichia coli*, *Klebsiella pneumoniae*, *Pseudomonas,* and *Acinetobacter*.^11^ Its emergence in Gram-negative bacteria is particularly alarming. NDM-1 consist of 258 amino acids that form 12 β-sheets and 6 α-helices, arranged in a αβ/βα fold with two zinc ions in the active center (Figure 1). The binding and hydrolysis of a broad variation of β-lactam antibiotics by NDM-1 are promoted by a number of structural features including the flexibility of loop three, which contains moieties offering opportunities for hydrophobic interactions. Thereto, the amino acids of flexible loop 10 may form hydrogen bonds. Furthermore, the 520 Å^2^ surface area of this enzyme is large as compared to other MBLs and is believed to be among the structural features responsible for the wide resistance spectrum of NDM-1.

**Figure 2:**
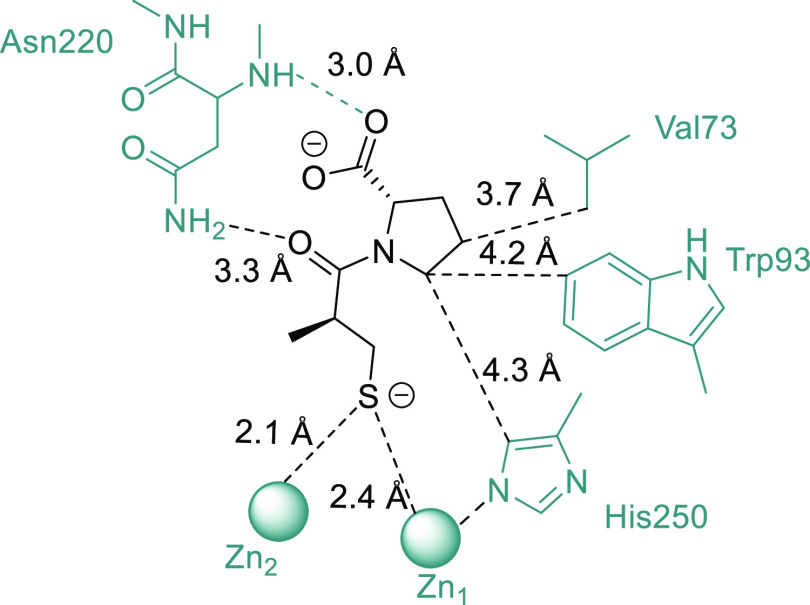
Binding mode of d-captopril in the active site of NDM-1 (PDB: 4exs).

Although over the past decade, a number of potential NDM-1 inhibitors have been disclosed, none have yet reached the clinical practice.^14^ They have so far been designed to mimic existing antibiotics and inhibitors or the transition state of the enzyme–substrate complex.^14^ Compounds with a stronger binding affinity than that of the native substrate may be achieved by mimicking the structure of the substrates in the first transition state of the β-lactam hydrolysis. The three main types of MBL inhibitors described in the literature so far are either (i) sequester, (ii) or coordinate the zinc ion(s) (d-captopril, Figure 2), or (iii) create a covalent bond with the protein, each approach having its advantages and disadvantages. For instance, there are multiple examples of chelating reagents with high inhibitory activity; however, these typically suffer from off-targets effects toward other metalloenzymes.^15^ The design of new inhibitors is promoted by the availability of zinc-coordinating inhibitors in the literature; however, that of covalent binders remains highly challenging. Accordingly, most inhibitors proposed so far bind non-covalently. Phosphonamidates have so far barely been studied for potential MBL inhibition.^16^

## Results and Discussion

**Figure 3:**
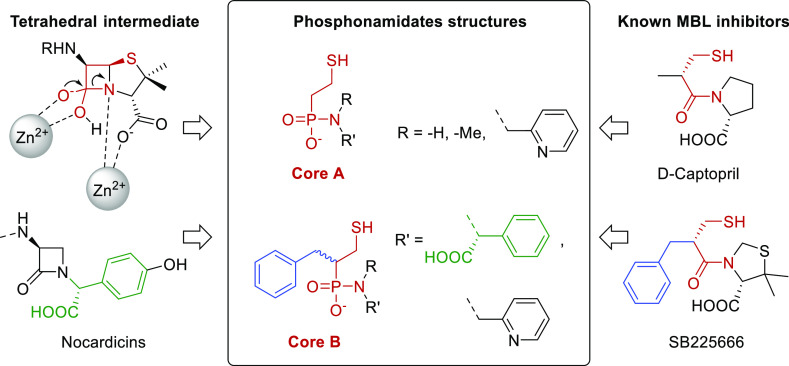
Applied design of phosphonamidate-based MBL inhibitors, with two different core structures, and with the common moieties being highlighted.^17,18^

Our design of new inhibitors was inspired by the catalytic mechanism of NDM-1^10^ and by the structure of known NDM-1 substrates (Figure 3). Accordingly, we anticipated that the incorporation of a phosphonamidate moiety, which possesses a central phosphorus atom that adopts a tetrahedral geometry and thus mimics the transition state of β-lactam hydrolysis, whereas not being hydrolysable, may be advantageous. To increase the binding affinity, we attached this to a 2-mercaptoethyl moiety. The latter is a common feature of many MBL inhibitor scaffolds as it can coordinate with the zinc ions, displacing a bridging water molecule or a hydrogen bond to Asn220. We selected the (2-mercaptoethyl)phosphonamidate (core A) and the (1-mercapto-3-phenylpropan-2-yl)phosphonamidate (core B) moieties as scaffolds. The lipophilic benzyl moiety of the latter was expected to interact with Trp93 of loop L3, which has been reported to facilitate the binding of β-lactam antibiotics.^14^ Coupling a variety of amines to both types of phosphonic monoacids resulted in nine compounds, whose synthesis is outlined below.

### Synthesis

**Scheme 1:**
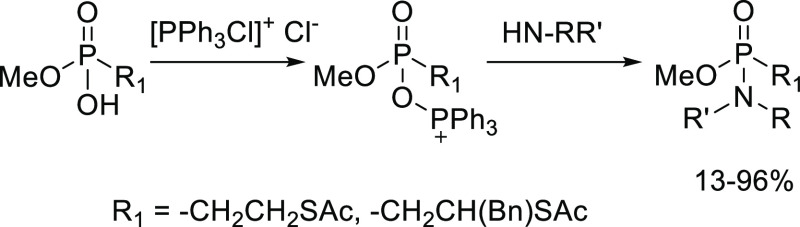
Two Consecutive Synthetic Steps in the Formation of the Phosphonamidates

**Scheme 2:**
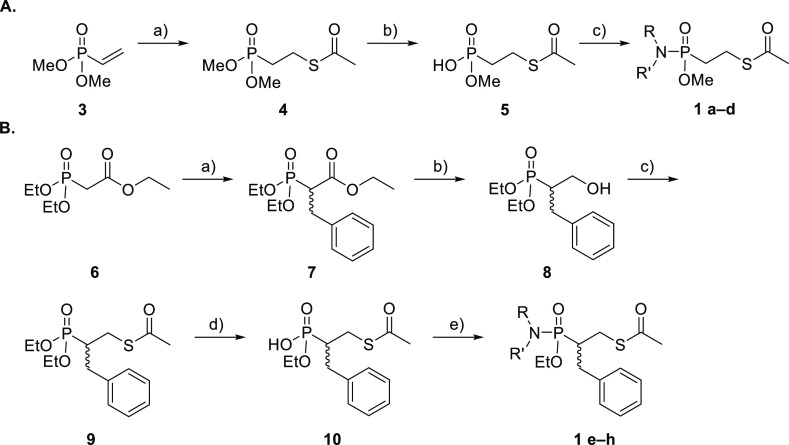
Reagents and conditions: (A) (a) thioacetic acid, CHCl_3_, 60 °C, 7 days, 51%; (b) NaI, acetone, 60 °C, o.n., 68%; (c) selected amine, PPh_3_Cl_2_, Et_3_N, DCM, r.t., Ar, o.n., 13–86%. (B) (a) Benzyl bromide, NaH, dry DME, 0 °C to r.t., o.n., 41%; (b) 2 M LiBH_4_ in THF, −20° C to rt, o.n., 91%; (c) thioacetic acid, DEAD, PS-PPh_3_, dry THF, −5 °C to r.t., on, 59%; (d) LiBr, 2-butanone, 80 °C, o.n., 96%; (e) NH-RR′, PPh_3_Cl_2_, Et_3_N, DCM, r.t., Ar, o.n., 19–96%.

**Scheme 3:**
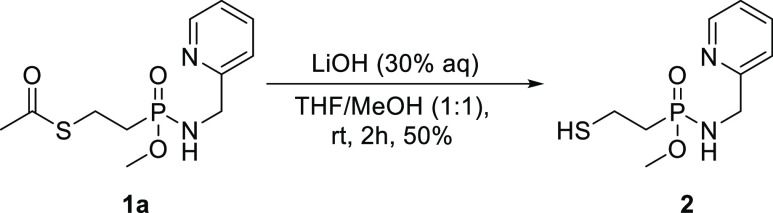
Deacetylation of the Thiol Moiety of **1a**

The key phosphonamidate moiety was obtained by coupling a monophosphonic acid to an amine using dichlorotriphenylphosphorane (PPh_3_Cl_2_) as a coupling agent (Scheme 1). A first series of phosphonamidates (Scheme 2A) was prepared in a three-step synthesis initiated by the condensation of dimethyl vinylphosphonate **3** and thioacetic acid in chloroform, using the sulfa-Michael addition, to form a new C–S bond in moderate yield 51%. Selective monohydrolysis of phosphonic ester **4** was obtained with sodium iodide, yielding monophosphonic acid **5** in 68% yield. Phosphonamidates **1a–d** were subsequently formed in 13–86% yields, following HPLC purification, as outlined in Scheme 1. To obtain phosphonamidates **1e–h**, a five-step synthesis was performed. First, triethyl phosphonoacetate was alkylated using KO*t*Bu or NaH in tetrahydrofuran (THF), dimethylformamide, or 1,2-dimethoxyethane (DME). The highest ratio of the mono- and di-alkylated species (3:1) was obtained in DME with NaH at 0 °C. Next, the ester of **7** was reduced to the corresponding alcohol **8** with LiBH_4_ in 91% yield. The Mitsunobu reaction, using immobilized triphenylphosphine, provided thioester **9** (59%). Analogous to the first synthetic pathway, **1e–h** were obtained in 19–96% yield by hydrolysis of phosphonic ester **9** to monoacid **10** using LiBr (96%), followed by phosphonamidation (Scheme 1). All compounds were prepared as racemic mixtures. In order to evaluate whether protection of the thiol of **1** is important, **1a** was deacetylated with LiOH in THF/MeOH (Scheme 3) resulting in **2** in 87% yield, following HPLC purification.

### MBL Inhibition Assay

The inhibitory activities of **1a–h** and **2** against the purified NDM-1, GIM-1, and VIM-2 enzymes were evaluated (IC_50_ data in Table 1). The inhibitory activity was measured by monitoring nitrocefin hydrolysis for VIM-2 and GIM-1 and that of imipenem for NDM-1^19^ at λ 482 and 300 nm, respectively. Five compounds showed inhibition of at least one of the tested MBL enzymes. Deacetylation of the thioester of **1a**, the most active compound toward GIM-1, to **2** possessing a free thiol, caused unexpected loss of inhibitory activity. This may indicate that unlike the sulfur of captopril, that of **1a** and **2** might not coordinate the zinc ions in the active site. Alternatively, this may be a result of disulfide formation under the conditions of the enzyme assay. The hydrophobic **1f–h** precipitated during the enzyme assay and their inhibitory activities could therefore not be reliably measured. This is not unusual as the binding cleft of MBL inhibitors is hydrophobic, and accordingly, their inhibitors typically have poor aqueous solubility.^20^ The inhibitors may be solubilized by careful dilution of dimethylsulfoxide (DMSO) stock solutions; however, this was not successful for compounds **1f–h**. None of the compounds showed significant MBL inhibitor activity when tested in *E. coli* in combination with meropenem, nor significant cytotoxicity against HeLa cells (for details, see the Experimental Methods section and Section S4 in the Supporting Information).

**Table 1:**
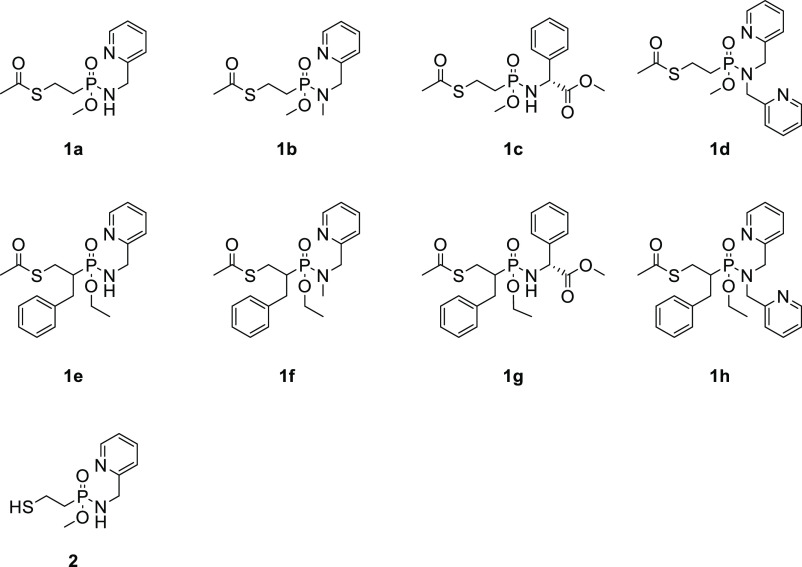
Inhibitory Concentration of Phosphonamidates (**1–2**) (IC_50_) against the Purified Enzymes NDM-1, GIM-1, and VIM-2^a^

	IC_50_ (μM)								
enzyme	**1a**	**1b**	**1c**	**1d**	**2**	**1e**	**1f**	**1g**	**1h**
NDM-1	N.I.	**356**	N.I	**430**	N.I.	N.I.	P.	P.	P.
GIM-1	**86**	**109**	N.I.	N.I.	N.I.	**2700**	P.	P.	P.
VIM-2	N.I.	**1417**	**500**	N.I.	N.I.	N.I.	P.	P.	P.

a IC_50_ = app*K*_i_ + (*E*_t_/2), where appK_i_ is the apparent *K*_i_, and *E*_t_ is the enzyme concentration. Accordingly, when the observed IC_50_ is much greater than the enzyme concentration, it is determined by the apparent *K*_i_ and not by the enzyme concentration.^21^ In the measurements mentioned above, the enzyme concentration has been at least 3 orders of magnitude below the observed IC_50_ values, and hence, the reported IC_50_s are in no mean affected by the NDM-1, GIM-1, and VIM-2 concentrations.

In order to identify the binding site of phosphonamidate monoesters to NDM-1, we optimized the expression of ^15^N- and ^13^C,^15^N-labeled NDM-1, performed the backbone NMR assignment of the protein, and studied the protein–ligand complex by solution NMR spectroscopy. A number of X-ray crystallographic structures of NDM-1^22^ and its complexes with a variety of substrates were available,^23−27^ whereas solution structures remain scarce.^20^ This is most likely due to the expression of isotopically labeled NDM-1 and its stabilization in solution being cumbersome. The conditions necessary for stabilizing a functional enzyme for solution NMR studies along with its backbone resonance assignment (89%) have first been very recently reported.^20^

### Protein Expression

U-[^13^C,^15^N]-labeled NDM-1 with a PelB leader sequence was expressed in 9 × 1 L M9 medium with ^15^N-labeled ammonium chloride and ^13^C-glucose at 18 °C, at 120 rpm in 1 L cultures. At OD_600_ = 1, IPTG was added at a final concentration of 1 mM, and expression was allowed to continue for 19 h before the cells were harvested. After extraction of the periplasmic content by osmotic shock and centrifugation, NDM-1 was purified on a 5 mL HisTrap HP column using ÄKTA Avant systems. Peak fractions were pooled and digested with tobacco etch virus (TEV) protease. After TEV digestion, TEV was removed by reverse IMAC. The flow-through and wash fractions were collected, dialyzed to 20 mM KPO_4_ and 0.1 mM ZnCl_2_, pH 7.0, and concentrated. Aliquots were prepared and snap frozen in liquid nitrogen. The final protein purity was estimated to 95%, using SDS-PAGE analysis, concentrated to 11.7 mg/mL.

### NMR Backbone Resonance Assignment of NDM-1

**Figure 4:**
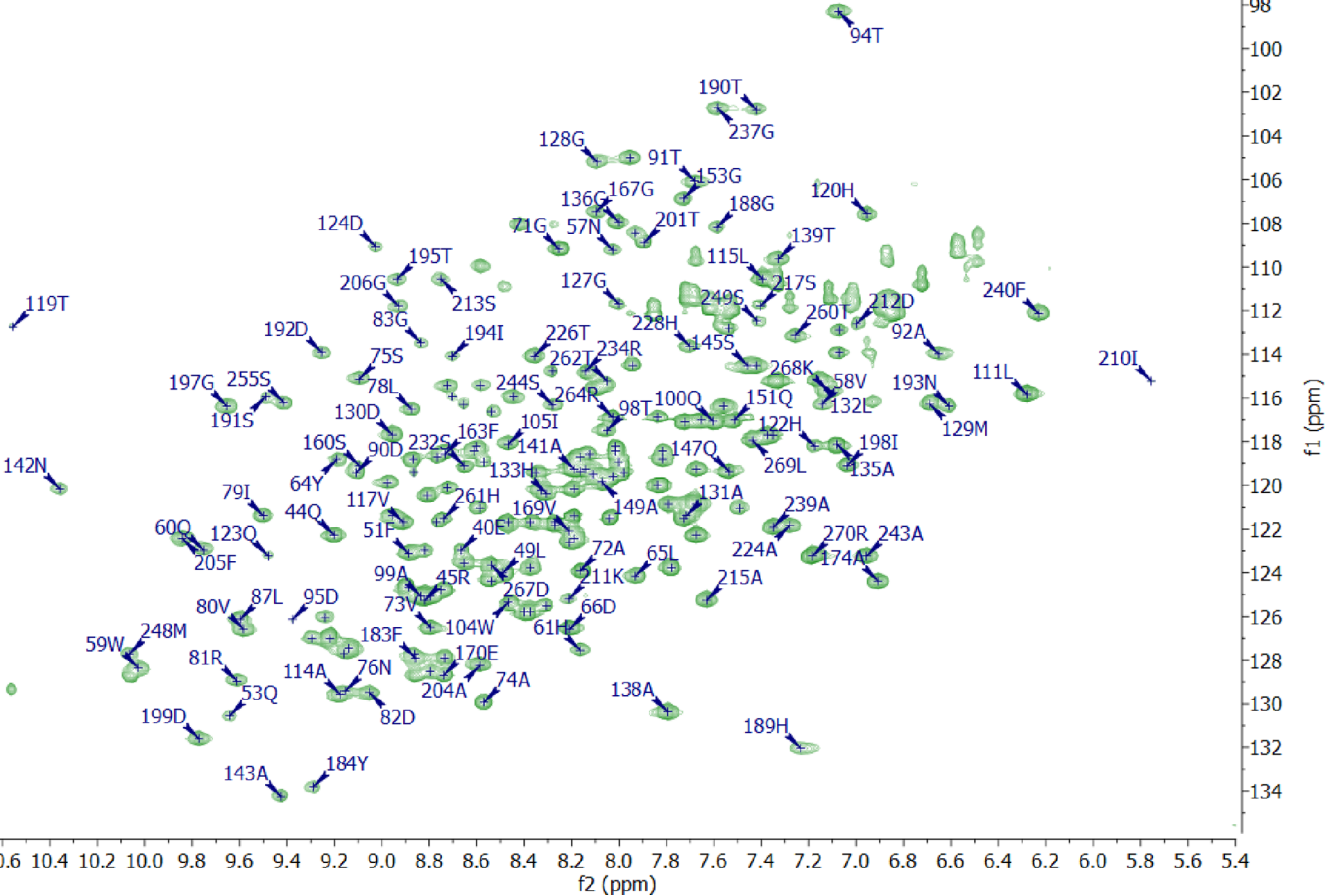
^1^H,^15^N HSQC spectrum (800 MHz, 37 °C) of NDM-1 showing the assignment. Expansion of the overlapping region is shown in Figure S1 in the Supporting Information.

Upon acquisition of ^1^H,^15^N HSQC, HNCO, HNcaCO, HNCA, HNcoCA, HNCACB, and HNcoCACB spectra on ^15^N,^13^C-labeled NDM-1 (0.5 mM) at 800 MHz at 37 °C, 92% chemical shifts of the amides (excluding prolines), 95% of Cα and Cβ, and 94% of CO residues were assigned (Figure 4). Spectra were acquired with targeted acquisition^28^ and 50% non-uniform sampling with the no-repeat shuffle (up to 70%, then shuffle) sampling scheme in an overall time of 10 days. In our hands, NDM-1 decomposed in ∼4 days at 37 °C, presumably due to self-cleavage at the G219-N220 amino acid pair that has previously been reported to cause instability.^29^ Therefore, three samples were used (prepared from the same expression batch) during the data acquisition. We achieved the previously missing assignments^30^ of E40-W59, N57, V58, Q107-L111, N176-F177, S217, I246, V247, and L269. The resonance assignments of G36-M39, G69, K125-M126, A165, G186, G200, G207-C208, K216, L218-L221, H250, and A251 were not possible, most likely due to conformational dynamics-induced line broadening under the conditions of data acquisition.

### NMR Characterization of Phosphonamidate Binding

**Figure 5:**
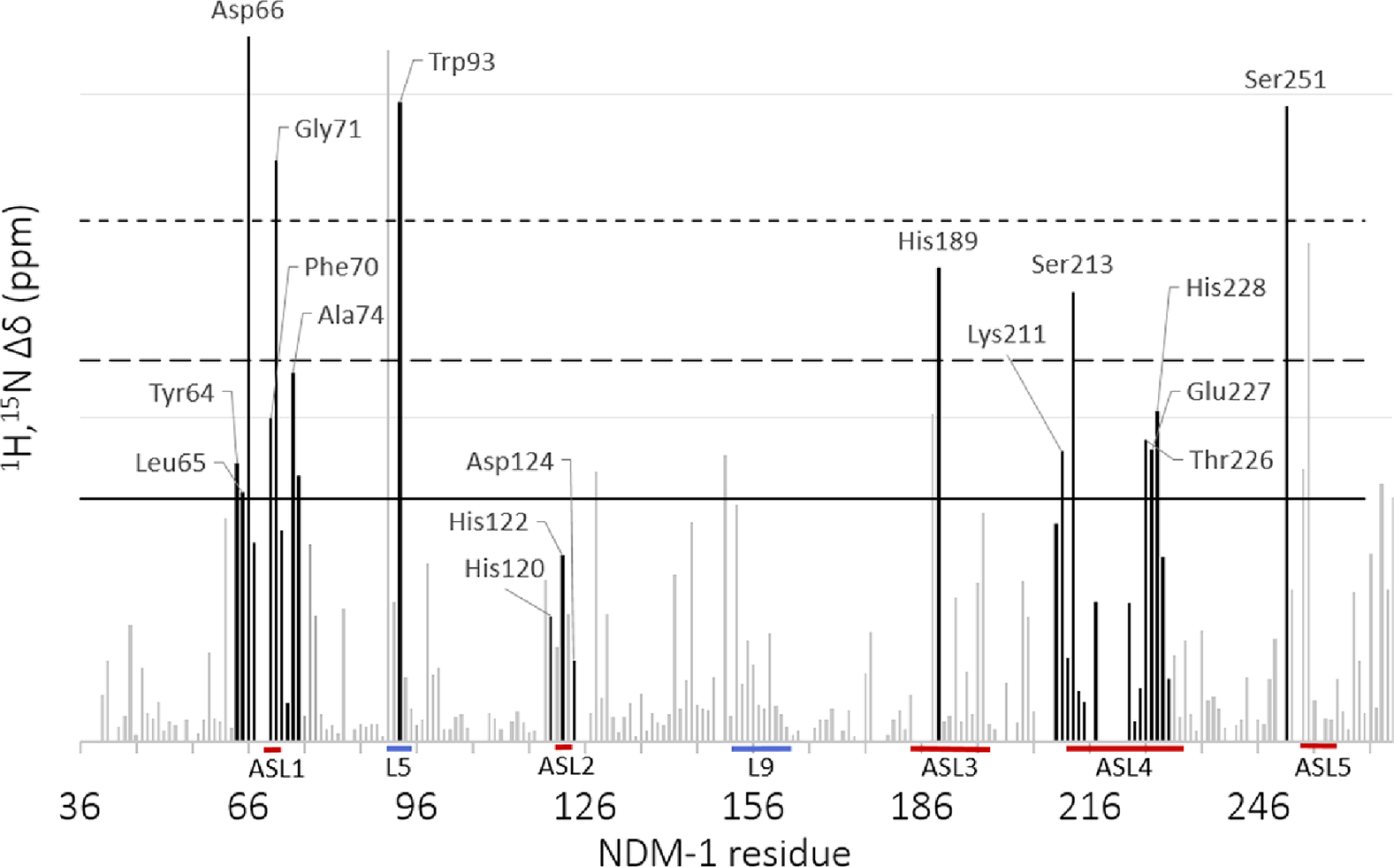
Chemical perturbation of the backbone amides (CSP) of ^15^N-labeled NDM-1 upon addition of 10 equiv of **1d**. Black bars indicate the residues that according to the literature are expected to take part in the interaction with the NDM-1 active site.^32^ Residues above the first horizontal cutoff are greater than the population mean plus the standard deviation (μ + 1σ) and therefore are considered to be significantly influenced by ligand binding. The solid and dashed lines represent the population mean (μ) plus one, two, and three standard deviations (σ), respectively.

**Figure 6:**
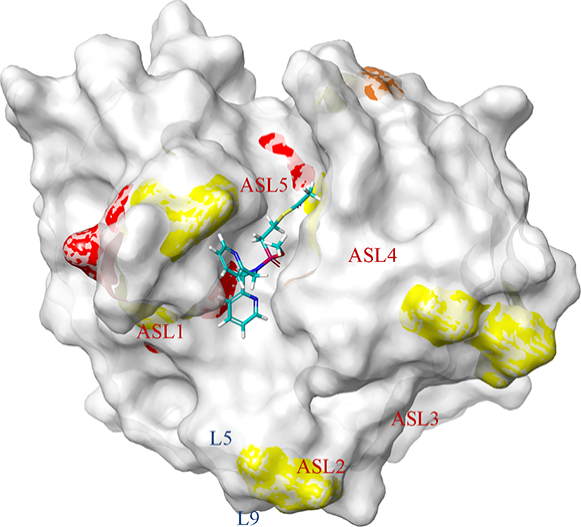
Amino acids of NDM-1 (PDB: 4hl2) that showed Δδ_1H,15N_ greater than the population mean plus the standard deviation (μ + 1σ—yellow, μ + 2σ—orange, and μ + 3σ—red) upon titration with **1d** (L5: Asp89-Trp93, L9: Met154-Gln158, numbered according to ref (20)).

**Figure 7:**
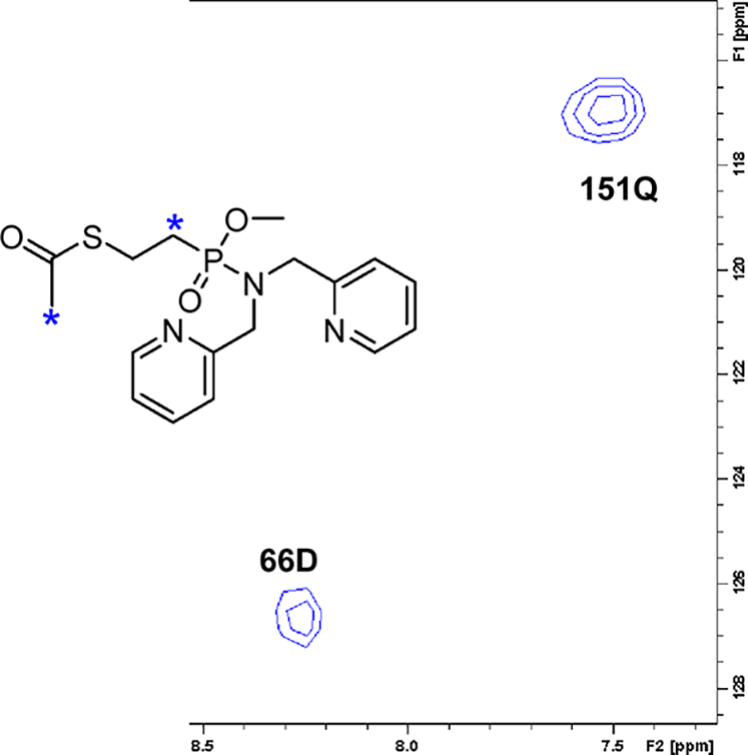
Expansion of the 2D plane of 3D-^1^H,^15^N,^1^H-HSQC-NOESY acquired on NDM-1 (0.25 mM) with addition of **1d** in a 1:8 molar ratio. The F_1_ × F_2_ plane at δ (F_3_, 2.40 ppm) corresponding to the C*H*_*3*_ and P–C*H*_*2*_ signals (*) of **1d** is shown. Cross-peaks available in this plane, assigned to Asp66 (66D) and Gln151 (151Q) of NDM-1, corroborate the binding of **1d** to NDM-1.

As binding-induced protein NMR chemical shift changes reveal the alteration of the local environment in the vicinity of the monitored nuclei, these are commonly acquired for identification of binding clefts. We monitored the weighted chemical shift changes, Δδ_1H,15N_, of uniformly ^15^N-enriched backbone amide functionalities upon successive additions of the 0–2.370 mM ligand using ^1^H,^15^N HSQC experiments (Figure 5). Following literature examples,^31^ chemical shift perturbations (CSPs) were considered to be significant (SSPs) in case the observed Δδ_1H,15N_ was greater than the population mean plus the standard deviation (μ + 1σ). Such chemical shift changes may directly originate from the interaction or might be indirect and thus be the result of binding-induced conformational changes. Based on its activity against NDM-1 (Table 1) and solubility, **1d** was selected for detailed analysis (Figure S2, Supporting Information). Similar to reported NDM-1 binding substances,^20^ it showed limited solubility, and accordingly, aggregation was observed in 20 mM K_3_PO_4_ aqueous buffer solution at pH 7 (Figure S2, Supporting Information).^33^ In order to exclude aggregation of **1d**, we also performed additional ^1^H,^15^N HSQC titration experiments using 5% DMSO or 5% ethanol (EtOH). Some minor chemical shift variations were observed upon the addition of the co-solvents, as expected,^34^ whereas neither protein precipitation nor alteration of the overall structure of NDM-1 was detected (Figures S3 and S4, Supporting Information). The chemical shift changes of 234 backbone amides were monitored. Five of them showed CSPs larger than the population mean plus three standard deviations (μ + 3σ), of which Asp66, Gly71, Trp93, and Ser251 have previously been reported to directly take part in interaction with NDM-1 inhibitors.^32^ A number of additional amino acids in the Zn^2+^-containing active site showed CSPs larger than μ + σ, as shown in Figures 6 and 7. This analysis revealed the interaction site of **1d** with NDM-1. Hence, the amino acids involved in the interaction belong mainly to mobile active site loops L1, L3, and L5, whereas those of active site loop L4 showed Δδ_1H,15N_ < μ + 1σ. The highest CSPs were observed for Asp66 of active site loop L1, Trp93 of L5, and Ser251 of active site loop L5. This observation is in good agreement with the previous reports on the location of the inhibitor binding site of NDM-1.^20^ The chemical shift changes localized to specific amino acids of active site loops L1-5 and loop L5 indicate specific binding and also the lack of larger structural rearrangements of NDM-1 upon **1d** binding.

Additional information about the binding event was obtained from 3D-^15^N-filtered HSQC-NOESY (Figure S6, Supporting Information). In such a filtered NOESY experiment, only nuclear overhauser effects (NOEs) between the uniformly ^15^N-labeled protein and the isotopically unlabeled ligand are detected, whereas intramolecular (protein–protein, ligand–ligand) NOEs, which could complicate the interpretation of the data due to signal overlaps, are filtered out.^35^ Thus, in a filtered NOE experiment, only intermolecular cross-peaks are detected. The 3D-^15^N-filtered HSQC-NOESY experiment allowed the observation of NOE cross-peaks between the S-acetyl CH_3_ resonances of **1d** at 2.42 ppm and the backbone amide of amino acid Asp66, a bridging CH_2_ of **1d** (2.38 ppm) and Gln151 (Figure 7), and between the ortho-aromatic pyridine proton of **1d** (7.38 ppm) and Trp93 of NDM-1. The amino acids Asp66 and Trp93 also exhibited large Δδ_1H,15N_ and hence corroborate the chemical shift titration-based identification of the binding cleft. The observation of the NOE to Gln151 was unexpected and may be due to signal overlap to an unassigned side chain amide or might possibly indicate aggregation, which, however, has not been indicated by any other NMR experiments.

**Figure 8:**
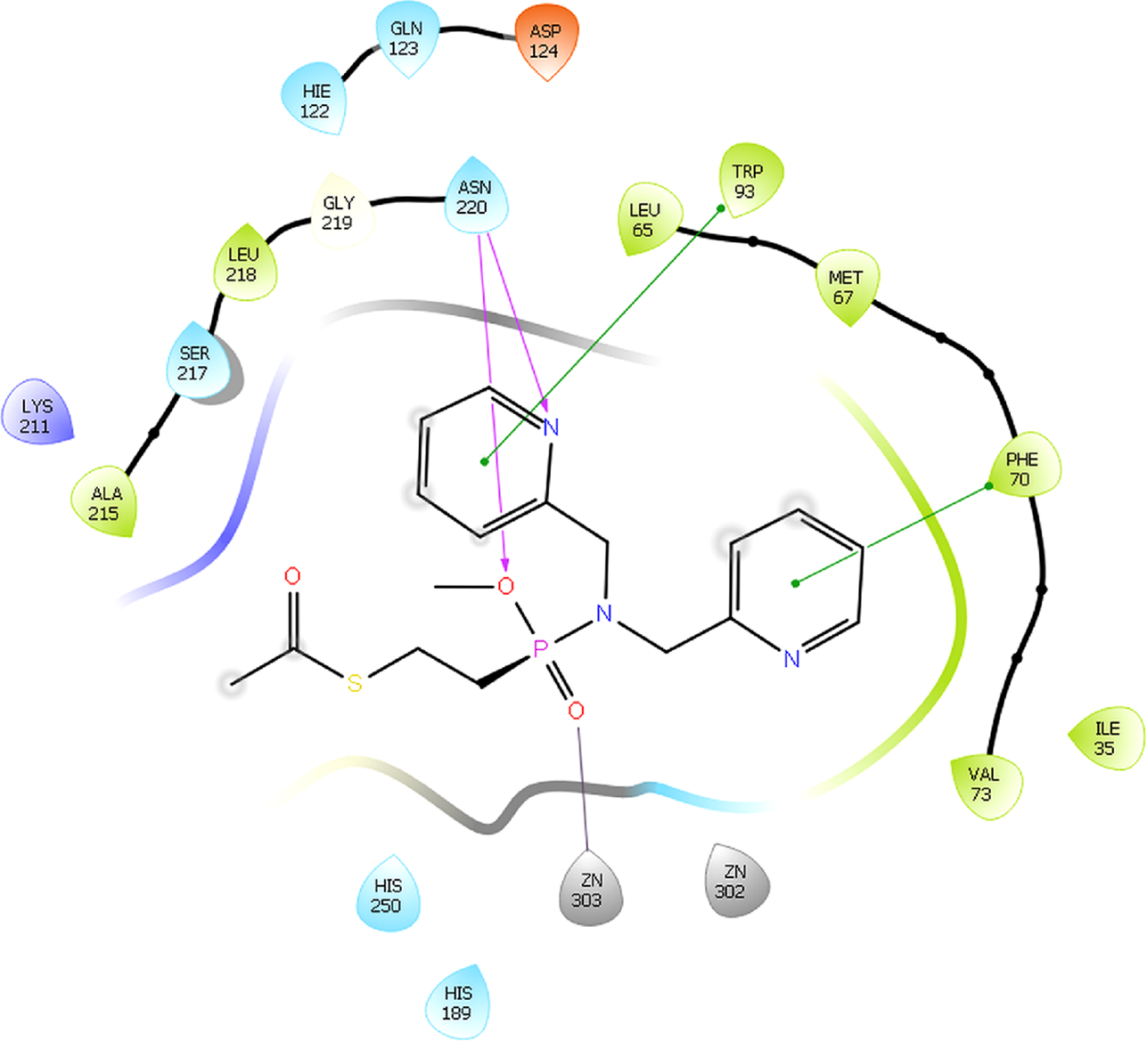
Interactions determined in the docking studies between NDM-1(PDB id: 4hl2) and **1d**.

**Docking** of **1d** to NDM-1 was performed using software Glide (Schrödinger Inc.) with a flexible docking algorithm starting from the PDB structure 4hl2, followed by MM-GBSA rescoring, with the resulting complex being shown in Figure 8. The binding pose was selected based on the observed intermolecular NOEs and the binding-induced NMR chemical shifts. It is in agreement with the previous literature, and hence, the amino acids Phe70, Trp93, and Asn220 that have been proposed to constitute the binding interface of NDM-1 showed binding-induced CSPs in our chemical shift titration experiment. Interaction with Zn^2^ was confirmed by the CSP of the coordinating residue His189. As the NDM-1 substrate binding site is comparably large, hydrophobic, and flexible and the binding of **1d** is weak (430 μM) and is expectably dominated by hydrophobic contacts, **1d** may reorient in the active site without larger energetic penalty.

Hence, whereas the chemical shift titration and NOE cross-peaks unambiguously locate the binding site, the proposed binding pose of **1d** should be seen as a model. Overall, the data indicate that **1d** is located in the substrate binding pocket of NDM-1 close to the Zn^2+^ ions that are expected to play a key role in β-lactam hydrolysis. Importantly, no sign for enzymatic modification of the structure of **1d** during the NMR studies could be observed.

## Conclusions

An efficient synthetic procedure to generate potential phosphonamidate inhibitors of MBLs has been developed. Five of the synthesized compounds showed activity toward at least one MBL in an enzyme assay, with the most active compound having an IC_50_ of 86 μM against GIM-1. Importantly, none of the studied compounds showed significant cytotoxicity against HeLa cells. Two phosphonamidate esters inhibited the clinically most relevant NDM-1, and the binding site of that possessing higher aqueous solubility was identified using solution NMR spectroscopy. A CSP pointed out active site loops 1, 3, and 5 and loop 5 as the binding interface of NDM-1 to phosphonamidate inhibitor **1d**. The location of the binding cleft was further corroborated by 3D ^15^N-filtrated HSQC-NOESY. Using molecular docking, a plausible binding mode of **1d** to NDM-1 was constructed. It indicates that **1d** binds in the hydrophobic substrate binding site of NDM-1, which has previously been proposed for antibiotics and other types of inhibitors, thus close to the catalytically important Zn^2+^ ions. The binding likely is largely driven by hydrophobic interactions. Upon further optimization, phosphonamidates might become a new potent class of the transition state mimicking MBL inhibitors.

## Experimental Methods

### General Methods

Starting materials were purchased from commercial suppliers and were used without further purification. Reactions were monitored by LCMS (Agilent 1100 Series) equipped with an ESI-MS detector (Waters Micromass ZQ 2000) or by TLC-MS (API, Advion Expression). The phosphonamidate series were purified with preparative RP-HPLC (VWR LaPrep P110) with single wavelength detection (254 nm), using an ACE5 C8 column (5 μm, 100 Å, ϕ 21.2 mm L 250 mm) and gradients of CH_3_CN/H_2_O as the mobile phase at a 10 mL/min flow rate. NMR spectra of the synthetic intermediates were recorded on a Varian Unity 400 MHz, Bruker Avance Neo 500 MHz, or a Bruker Avance Neo 600 MHz spectrometer. The Bruker instruments were equipped with TXO and TCI cryogenic probes. The chemical shifts are reported using the residual solvent signal as an indirect reference to TMS. Chemical shift titrations and 3D NOESY were acquired on the Bruker Avance Neo 600 MHz spectrometer, whereas spectra for assignment were obtained on the Bruker Avance HD 800 MHz spectrometer equipped with a 3 mm TCI cryogenic probe. Purity analysis of the final phosphonamidate inhibitors was performed using ^1^H NMR, with the original spectra being shown in the Supporting Information and the original NMR raw data files (FID) available open access at Zenodo (DOI:10.5281/zenodo.4773990).

### Synthetic Procedures

The synthesis of compounds **1a–h** is shown in Scheme 2, with details being given below.

#### 2-(Dimethoxyphosphoryl)ethyl Ethanethioate (**4**)

In a 20 mL microwave vial kept under argon, thioacetic acid (1.92 g, 25.3 mmol, 1.00 equiv) was added to a solution of dimethyl vinylphosphonate (1.72 g, 12.6 mmol, 1.00 equiv) in 10 mL of chloroform. The reaction mixture was stirred at 60 °C for 7 days. The solvent was evaporated, and the residue was purified on silica gel, using DCM/MeOH (98:2). The product was obtained as a yellowish oil (1.52 g, 51%). ^1^H NMR (600 MHz, CDCl_3_, 25 °C): δ 3.76 (d, ^3^*J*_H,P_ = 10.9 Hz, 6H, OC*H*_*3*_), 3.10–3.01 (m, 2H, C*H*_*2*_), 2.33 (s, 3H, C*H*_*3*_), 2.10–2.02 (m, 2H, P–C*H*_*2*_). ^13^C NMR (151 MHz, CDCl_3_, 25 °C): δ 195.4 (*C*=O), 52.7 (d, *J* = 6.5 Hz, O*C*H_3_), 30.7 (d, ^3^*J*_P,C_ = 0.9 Hz, *C*H_3_), 25.6 (d, ^1^*J*_P,C_ = 137.2 Hz, P–*C*H_2_), 22.7 (d, ^2^*J*_*P*,*C*_ = 3.3 Hz, *C*H_2_). ^31^P NMR (162 MHz, CDCl_3_, 25 °C): δ 30.4. HRMS (ESI-Q-TOF) C_6_H_13_O_4_PS *m*/*z*: [M + H]^+^ calcd, 213.0350; found, 213.0326.

#### 2-[Hydroxy(methoxy)phosphoryl]ethyl Ethanethioate (**5**)

*S*-[2-(Dimethoxyphosphoryl)ethyl] ethanethioate (1.36 g, 6.4 mmol, 1 equiv) was dissolved in 30 mL of acetone, and sodium iodide (0.82 g, 5.45 mmol, 0.85 equiv) was added to the mixture. The reaction mixture was stirred at 60 °C for 48 h. The sodium salt of the product (white precipitate) was filtrated, dissolved in 0.1 M HCl, and extracted three times with EtOAc. The organic phases were combined, dried over anhydrous sodium sulfate, filtrated, and evaporated. The product was obtained as a yellowish oil (0.97 g, 68%). ^1^H NMR (400 MHz, CDCl_3_, 25 °C): δ 11.95 (s, 1H, O*H*), 3.76 (d, ^3^*J*_H,P_ = 11.2 Hz, 3H, OC*H*_*3*_), 3.14–3.03 (m, 2H, C*H*_*2*_), 2.33 (s, 3H, C*H*_*3*_), 2.15–2.02 (m, 2H, P–C*H*_*2*_).^13^C NMR (101 MHz, CDCl_3_, 25 °C): δ 195.2 (*C*=O), 52.0 (d, ^3^*J*_P,C_ = 6.8 Hz, O*C*H_3_), 30.7 (*C*H_3_), 26.3 (d, ^1^*J*_P,C_ = 140.4 Hz, P–*C*H_2_), 22.5 (d, ^2^*J*_P,C_ = 2.8 Hz, *C*H_2_). ^31^P NMR (162 MHz, CDCl_3_, 25 °C): δ 31.8. HRMS (ESI-Q-TOF) C_5_H_11_O_4_PS *m*/*z*: [M + H]^+^ calcd, 199.0194; found, 199.0199.

#### Ethyl 2-(Diethoxyphosphoryl)-3-phenylpropanoate (**7**)

NaH (60% in mineral oil, 0.98 g, 24.53 mmol, 1.1 equiv) was suspended in 50 mL of anhydrous DME. The suspension was cooled to 0 °C, and a triethylphosphonoacetate (5.00 g, 22.30 mmol, 1 equiv) solution in anhydrous DME (5 mL) was added dropwise. The reaction was stirred for 1 h. Next, a solution of benzylbromide (3.81 g, 22.30 mmol, 1.0 equiv) in DME (5 mL) was added dropwise. During this time, a white precipitate (NaBr) was formed. The reaction was left to slowly warm to room temperature and stirred for 24 h (until full conversion of the starting material was observed). The solvent was removed in vacuum, and the crude mixture was diluted with EtOAc and washed with water (two times) and brine. The aqueous fraction was re-extracted twice with EtOAc. The combined organic layers were dried over Na_2_SO_4_, filtered, and evaporated. The crude product was purified on silica gel, using hexane/EtOAc 6:4. The product was obtained as a yellow oil (2.90 g, 41%). The dialkylated byproduct was present in certain batches. For characterization, one batch was purified by HPLC. For the rest of the batches, the byproduct was removed after the following step: ^1^H NMR (400 MHz, CDCl_3_, 25 °C): δ 7.29–7.24 (m, 2H, m-*H*_*Ar*_), 7.23–7.17 (m, 3H, o,p-*H*_*Ar*_), 4.17 (dq, ^2^*J*_P,H_ = 8.1, 7.5 Hz, 2H, P–OC*H*_*2*_–CH_3_), 4.17 (dq, ^2^*J*_P,H_ = 7.6, 7.2 Hz, 2H, P–OC*H*_*2*_–CH_3_), 4.10 (dddq, *J* = 6.9, 3.5, 3.5,3,1 Hz, 2H, OC*H*_*2*_–CH_3_), 3.30–3.24 (m, 1H, P–C*H*), 3.24–3.14 (m, 2H, C*H*_*2*_), 1.35 (t, *J* = 7.1 Hz, 3H, P–OCH_2_–C*H*_*3*_), 1.34 (t, *J* = 7.1 Hz, 3H, P–OCH_2_–C*H*_*3*_), 1.13 (t, *J* = 7.1 Hz, 3H, OCH_2_–C*H*_*3*_). ^13^C NMR (101 MHz, CDCl_3_, 25 °C): δ 168.5 (d, ^2^*J*_P,C_ = 4.5 Hz, *C*=O), 138.7 (d, ^3^*J*_P,C_ = 16.1 Hz, ipso-*C*_*Ar*_), 128.7 (o-*C*_*Ar*_), 128.6 (m-*C*_*Ar*_), 126.8 (p-*C*_*Ar*_), 63.0 (d, ^2^*J*_P,C_ = 6.4 Hz, P–O*C*H_2_–CH_3_), 62.9 (d, ^2^*J*_P,C_ = 6.7 Hz, P–O*C*H_2_–CH_3_), 61.5 (O*C*H_2_–*C*H_3_), 47.8 (d, ^1^*J*_P,C_ = 129.2 Hz, P–*C*H), 32.9 (d, ^2^*J*_P,C_ = 4.3 Hz, *C*H_2_), 16.6 (d, ^3^*J*_P,C_ = 6.0 Hz, P–OCH_2_–*C*H_3_), 16.5 (d, ^3^*J*_P,C_ = 5.8 Hz, P–OCH_2_–*C*H_3_), 14.1 (O–CH_2_–*C*H_3_). ^31^P NMR (162 MHz, CDCl_3_, 25 °C): δ 21.8. HRMS (ESI-Q-TOF) C_15_H_23_O_4_PS *m*/*z*: [M + H]^+^ calcd, 331.113; found, 331.1143.

#### Diethyl (1-Hydroxy-3-phenylpropan-2-yl)phosphonate (**8**)

Ethyl 2-(diethoxyphosphoryl)-3-phenylpropanoate (2.58 g, 8.21 mmol) was dissolved in dry THF. A solution of LiBH_4_ in THF (2.0 M, 2.6 mL, 1.5 equiv) was added under a nitrogen atmosphere at −20 °C over 10 min. The cold bath was then removed, and the reaction was stirred under an inert atmosphere at room temperature. After 47 h, the reaction mixture was cooled to −30 °C, and MeOH (15 mL) was added dropwise. The solvents were removed in vacuum, and the resulting yellow solid was taken up in water and extracted with EtOAc and further washed with water. The combined organic layers were dried over Na_2_SO_4_, filtered, and the solvent was removed in vacuum. The crude mixture was purified on silica gel using hexane/EtOAc (1:9). The product was obtained as a colorless oil (2.04 g, 91%). ^1^H NMR (500 MHz, CDCl, 25 °C): δ 7.30–7.24 (m, 2H, m-*H*_*Ar*_), 7.22–7.18 (m, 3H, o,p-*H*_*Ar*_), 4.15 (ddd, *J* = 14.9, 7.4, 3.2 Hz, 2H, P–OC*H*_*2*_–CH_3_), 4.12–4.07 (m, 2H, P–OC*H*_*2*_–CH_3_), 3.76 (ddd, *J* = 22.6, 11.6, 3.4 Hz, 1H, C*H*_*2*_–OH), 3.65 (ddd, *J* = 24.8, 11.6, 6.5 Hz, 1H, C*H*_*2*_^′^–OH), 3.02 (ddd, *J* = 14.5, 10.3, 4.5 Hz, 1H, C*H*_*2*_), 2.77 (ddd, *J* = 14.1, 10.4, 10.4 Hz, 1H, C*H*_*2*_^′^), 2.70 (s, 1H, O*H*), 2.21 (ddddd, *J* = 17.2, 12.7, 6.5, 4.1, 3.6 Hz, 1H, P–C*H*), 1.34 (t, *J* = 7.1 Hz, 3H, P–OCH_2_–C*H*_*3*_), 1.30 (t, *J* = 7.0 Hz, 3H, P–OCH_2_–C*H*_*3*_). ^13^C NMR (126 MHz, CDCl_3_, 25 °C): δ 138.8 (d, ^3^*J*_P,C_ = 14.7 Hz, ipso-*C*_*Ar*_), 129.1 (o-*C*_*Ar*_), 128.6 (m-*C*_*Ar*_), 126.6 (p-*C*_*Ar*_), 62.3 (d, ^2^*J*_P,C_ = 6.9 Hz, P–O*C*H_2_–CH_3_), 62.1 (d, ^2^*J*_P,C_ = 6.7 Hz, P–O*C*H_2_–CH_3_), 59.8 (d, ^2^*J*_P,C_ = 5.6 Hz, *C*H_2_–OH), 41.2 (d, ^1^*J*_P,C_ = 136.2 Hz, P–*C*H), 31.4 (d, ^2^*J*_P,C_ = 2.7 Hz, *C*H_2_), 16.6 (d, ^3^*J*_P,C_ = 8.2 Hz, P–OCH_2_–*C*H_3_), 16.5 (d, ^3^*J*_P,C_ = 8.7 Hz, P–OCH_2_–*C*H_3_). ^31^P NMR (162 MHz, CDCl_3_, 25 °C): δ 32.0. HRMS (ESI-Q-TOF) C_13_H_19_O_4_PS *m*/*z*: [M + H]^+^ calcd, 303.0820; found, 303.0820.

#### (*R*/*S*)-*S*-[2-(Diethoxyphosphoryl)-3-phenylpropyl] Ethanethioate (**9**)

Pre-dried polystyrene-supported PS-triphenylphosphine (6.91 g, 16.16 mmol, 2.20 equiv) was suspended in 50 mL of dry THF and was cooled to −5 °C. DEAD (40% in toluene, 7.36 mL, 16.16 mmol, 2.20 equiv) was added dropwise to the mixture. After 30 min, a solution of diethyl (1-hydroxy-3-phenylpropan-2-yl)phosphonate (2.00 g, 7.35 mmol, 1.00 equiv) in THF (5 mL) was added dropwise, followed by thioacetic acid (1.23 g, 16.16 mmol, 2.20 equiv). The reaction mixture was stirred over 2 h at −5 °C and then for 20 h at room temperature. The solid-supported PPh_3_ was filtered off, and the evaporated crude mixture was purified on silica gel using hexane/EtOAc (3:7). The product was obtained as colorless oil (1.43 g, 59%). ^1^H NMR (500 MHz, CDCl_3_, 25 °C): δ 7.31–7.26 (m, 2H, m-*H*_*Ar*_), 7.24–7.17 (m, 3H, o,p-*H*_*Ar*_), 4.10–4.00 (m, 4H, P–OC*H*_*2*_–CH_3_), 3.28 (ddd, *J* = 16.8, 13.9, 5.2 Hz, 1H, S–C*H*_*2*_), 3.11 (ddd, *J* = 14.2, 6.0 Hz, 1H, C*H*_*2*_), 3.01 (ddd, *J* = 13.3, 7.6 Hz, 1H, S–C*H*_*2*_^′^), 2.83 (ddd, *J* = 14.9, 7.8 Hz, 1H, C*H*_*2*_^′^), 2.39–2.31 (m, 1H, P–C*H*), 2.30 (s, 3H, C*H*_*3*_), 1.28 (t, *J* = 7.1 Hz, 3H, P–OCH_2_–C*H*_*3*_), 1.26 (t, *J* = 7.1 Hz, 3H, P–OCH_2_–C*H*_*3*_).^13^C NMR (101 MHz, CDCl_3_, 25 °C): δ 195.2 (*C*=O), 138.8 (d, *J* = 10.1 Hz, ipso-*C*_*Ar*_), 129.4 (o-*C*_*Ar*_), 128.5 (m-*C*_*Ar*_), 126.7 (p-*C*_*Ar*_), 62.1 (d, ^2^*J*_P,C_ = 2.9 Hz, P–OC*H*_*2*_–CH_3_), 62.0 (d, ^2^*J*_P,C_ = 2.9 Hz, P–OC*H*_*2*_–CH_3_), 38.6 (d, ^1^*J*_P,C_ = 138.8 Hz, P–*C*H), 34.2 (d, ^2^*J*_P,C_ = 2.9 Hz, *C*H_2_), 30.6 (CH_3_), 28.1 (d, ^2^*J*_P,C_ = 2.1 Hz, S–*C*H_2_), 16.5 (d, ^3^*J*_P,C_ = 6.0 Hz, P–OCH_2_–*C*H_3_), 16.5 (d, ^3^*J*_P,C_ = 6.0 Hz, P–OCH_2_–*C*H_3_).^31^P NMR (162 MHz, CDCl_3_, 25 °C): δ 29.5. HRMS (ESI-Q-TOF) C_15_H_23_O_4_PS *m*/*z*: [M + H]^+^ calcd, 331.1133; found, 331.1143.

#### (*R*/*S*)-{2-[Ethoxy(hydroxy)phosphoryl]-3-phenylpropyl} Ethanethioate (**10**)

*S*-[2-(Diethoxyphosphoryl)-3-phenylpropyl] ethanethioate (500 mg, 1.51 mmol, 1.00 equiv) was dissolved in butan-2-one (8 mL) in a 20 mL microwave vial. LiBr (184 mg, 2.12 mmol, 1.40 equiv) was added, and the reaction mixture was refluxed for 30 h. The lithium salt of the product was filtrated off as a white precipitate and was washed with butan-2-one. The salt was dissolved in 0.1 M HCl and extracted with 3× EtOAc. The combined organic layers were dried over Na_2_SO_4_, filtrated, and evaporated. The product was obtained as a colorless oil (440 mg, 96%). ^1^H NMR (400 MHz, CDCl_3_, 25 °C): δ 7.80 (br s, 1H, O*H*), 7.34–7.28 (m, 2H, m-*H*_*Ar*_), 7.27–7.18 (m, 3H, o,p-*H*_*Ar*_), 4.17–3.98 (m, 2H, P–OC*H*_*2*_–CH_3_), 3.24 (ddd, *J* = 19.4, 13.7, 5.7 Hz, 1H, S–C*H*_*2*_), 3.15 (ddd, *J* = 13.9, 13.2, 5.6 Hz, 1H, C*H*_*2*_), 3.08 (ddd, *J* = 20.9, 14.0, 6.6 Hz, 1H, S–C*H*_*2*_^′^), 2.80 (ddd, *J* = 14.0, 8.7 Hz, 1H, C*H*_*2*_^′^), 2.38 (ddddd, *J* = 20.6, 8.5, 5.9, 5.9, 5.9 Hz, 1H, P–C*H*), 2.28 (s, 3H, C*H*_*3*_), 1.29 (t, *J* = 7.1 Hz, 3H, P–OCH_2_–C*H*_*3*_). ^13^C NMR (101 MHz, CDCl_3_, 25 °C): δ 195.1 (*C*=O), 138.6 (d, ^3^*J*_P,C_ = 11.8 Hz, ipso-*C*_*Ar*_), 129.3 (o-*C*_*Ar*_), 128.6 (m-*C*_*Ar*_), 126.8 (p-*C*_*Ar*_), 61.8 (d, ^2^*J*_P,C_ = 7.2 Hz, P–O*C*H_2_–CH_3_), 38.3 (d, ^1^*J*_P,C_ = 141.9 Hz, P–*C*H), 34.0 (d, ^2^*J*_P,C_ = 2.5 Hz, *C*H_2_), 30.6 (*C*H_3_), 27.7 (d, ^2^*J*_P,C_ = 2.2 Hz, S–*C*H_2_), 16.4 (d, ^3^*J*_P,C_ = 6.4 Hz, P–OCH_2_–*C*H_3_). ^31^P NMR (162 MHz, CDCl_3_, 25 °C): δ 32.9. HRMS (ESI-Q-TOF) C_13_H_19_O_4_PS *m*/*z*: [M + H]^+^ calcd, 303.0820; found, 303.0825.

### General Procedure for Phosphonamidation

The phosphonate monoester (core structure A or B) (1 equiv) was dissolved in dry DCM (10 mL), and PPh_3_Cl_2_ (1.5 equiv, 40% weight percentage, the substance is converted to O=PPh_3_ over time in the presence of water) was added, followed by dry trimethylamine (1.50 equiv). The mixture was stirred for 20 min at room temperature before it was added dropwise (speed 0.5 mL/min) to a solution of the amine (1.50 equiv) and Et_3_N (1.50 equiv) in 5 mL of dry DCM. The reaction mixture was stirred o/n at room temperature. After completion, monitored by LCMS, the solvent was evaporated, and the reaction mixture was purified using preparative HPLC (ACE 5 column, C8, 5 μm, 100 Å, ϕ 21.2 cm, L 25 cm) with MeCN/H_2_O using gradient elution (5% MeCN at 5 mL/min for 5 min, followed by 5–95% MeCN at 10 mL/min for 40 min).

#### (*R*/*S*)-(2-(Methoxy((pyridin-2-ylmethyl)amino)phosphoryl)ethyl) Ethanethioate (**1a**)

^1^H NMR (500 MHz, CDCl_3_, 25 °C): δ 8.55 (ddd, *J* = 5.0, 1.7, 0.8 Hz, 1H, m-*H*_*Ar6*_), 7.67 (ddd, *J* = 7.7, 7.6, 1.8 Hz, 1H, m-*H*_*Ar4*_), 7.30–7.27 (m, 1H, o-*H*_*Ar3*_), 7.23–7.17 (m, 1H, p-*H*_*Ar5*_), 4.35–4.20 (m, 2H, C*H*_*2*_), 3.78–3.70 (m, 1H, N*H*), 3.65 (d, *J* = 11.1 Hz, 3H, P–OC*H*_*3*_), 3.16–3.02 (m, 2H, S–C*H*_*2*_), 2.31 (s, 3H, C*H*_*3*_), 2.15–2.00 (m, 2H, P–C*H*_*2*_). ^13^C NMR (101 MHz, CDCl_3_, 25 °C): δ 195.6 (*C*=O), 157.9 (d, *J* = 5.8 Hz, ipso-*C*_*Ar2*_), 149.4 (m-*C*_*Ar6*_), 136.9 (m-*C*_*Ar4*_), 122.5 (p-*C*_*Ar5*_), 121.6 (o-*C*_*Ar3*_), 50.7 (d, *J* = 6.9 Hz, P–O*C*H_3_), 45.8 (*C*H_2_), 30.7 (*C*H_3_), 27.9 (d, ^1^*J*_P,C_ = 126.8 Hz, P–*C*H_2_), 22.9 (d, ^2^*J*_P,C_ = 2.1 Hz, S–*C*H_2_). ^31^P NMR (162 MHz, CDCl_3_, 25 °C): δ 32.6. HRMS (ESI-Q-TOF) C_11_H_17_N_2_O_3_PS *m*/*z*: [M + H]^+^ calcd, 289.0776; found, 289.0753.

#### (*R*/*S*)-(2-(Methoxy(methyl(pyridin-2-ylmethyl)amino)phosphoryl)ethyl) Ethanethioate (**1b**)

^1^H NMR (500 MHz, CDCl_3_, 25 °C): δ 8.56 (ddd, *J* = 4.9, 1.6, 0.9 Hz, 1H, m-*H*_*Ar6*_), 7.70 (ddd, *J* = 7.7, 7.7, 1.8 Hz, 1H, m-*H*_*Ar4*_), 7.41 (m, 1H, o-*H*_*Ar3*_), 7.20 (ddd, *J* = 7.5, 4.9, 1.2 Hz, 1H, p-*H*_*Ar5*_), 4.37 (d, *J* = 9.0 Hz, 2H, C*H*_*2*_), 3.64 (d, *J* = 11.1 Hz, 3H, P–OC*H*_*3*_), 3.12 (dt, *J* = 10.3, 8.1 Hz, 2H, S–C*H*_*2*_), 2.65 (d, *J* = 9.0 Hz, 3H, N–C*H*_*3*_), 2.33 (s, 3H, C*H*_*3*_), 2.12 (m, 2H, P–C*H*_*2*_). ^13^C NMR (126 MHz, CDCl_3_, 25 °C): δ 195.6 (*C*=O), 158.3 (d, ^3^*J*_P,C_ = 3.5 Hz, ipso-*C*_*Ar2*_), 149.3 (m-*C*_*Ar6*_), 137.0 (m-*C*_*Ar4*_), 122.5 (p-*C*_*Ar5*_), 122.4 (o-*C*_*Ar3*_), 54.1 (d, ^2^*J*_P,C_ = 4.0 Hz, *C*H_2_), 50.5 (d, ^2^*J*_P,C_ = 7.0 Hz, P–O*C*H_3_), 33.4 (d, *J* = 4.6 Hz, N–*C*H_3_), 30.7 (*C*H_3_), 26.7 (d, ^1^*J*_P,C_ = 127.6 Hz, P–*C*H_2_), 22.8 (d, ^2^*J*_P,C_ = 1.9 Hz, S–*C*H_2_). ^31^P NMR (162 MHz, CDCl_3_, 25 °C): δ 34.2. HRMS (ESI-Q-TOF) C_12_H_19_N_2_O_3_PS *m*/*z*: [M + H]^+^ calcd, 303.0932; found, 303.0909.

#### Methyl (*R*)-2-(((*R*/*S*)-(2-(Acetylthio)ethyl) (methoxy)phosphoryl)amino)-2-phenylacetate (Mixture of Two Diastereoisomers) (**1c**)

^1^H NMR (500 MHz, CDCl_3_, 25 °C): δ 7.40–7.29 (m, 10H, *H*_*Ar*_), 5.03 (dd, *J* = 9.5, 4.6 Hz, 1H, N–C*H*), 5.01 (dd, *J* = 9.3, 4.7 Hz, 1H, N–C*H*), 3.89 (t, *J* = 9.9 Hz, 1H, N*H*), 3.81 (t, *J* = 10.0 Hz, 1H, N*H*), 3.73 (s, 3H, OC*H*_*3*_), 3.72 (s, 3H, OC*H*_*3*_), 3.52 (d, *J* = 4.7 Hz, 3H, P–OC*H*_*3*_), 3.50 (d, ^3^*J*_P,H_ = 4.7 Hz, 3H, P–OC*H*_*3*_), 3.07–2.99 (m, 2H, S–C*H*_*2*_), 2.99–2.90 (m, 2H, S–C*H*_*2*_), 2.31 (s, 3H, C*H*_*3*_), 2.30 (s, 3H, C*H*_*3*_), 2.05–1.94 (m, 2H, P–C*H*_*2*_), 1.94–1.87 (m, 2H, P–C*H*_*2*_). ^13^C NMR (126 MHz, CDCl_3_, 25 °C): δ 195.54 (2 × *C*=O), 172.55 (d, ^3^*J*_P,C_ = 10.3 Hz, ester *C*=O), 172.53 (d, *J* = 10.4 Hz, ester, *C*=O), 138.60 (d, ^3^*J*_P,C_ = 3.2 Hz, ipso-*C*_*Ar*_), 138.57 (d, ^3^*J*_P,C_ = 3.1 Hz, ipso-*C*_*Ar*_), 129.16 (*C*_*Ar*_), 129.12 (*C*_*Ar*_), 128.63 (*C*_*Ar*_), 127.10 (*C*_*Ar*_), 127.08 (*C*_*Ar*_), 57.64 (d, ^2^*J*_P,C_ = 1.7 Hz, N–*C*H), 57.59 (d, ^2^*J*_P,C_ = 2.4 Hz, N–*C*H), 53.09 (O*C*H_3_), 53.04 (O*C*H_3_), 50.90 (d, ^2^*J*_P,C_ = 7.0 Hz, P–O*C*H_3_), 50.74 (d, ^2^*J*_P,C_ = 7.0 Hz, P–O*C*H_3_), 30.66 (2 × *C*H_3_), 28.45 (d, ^1^*J*_P,C_ = 127.5 Hz, P–*C*H_2_), 28.34 (d, ^1^*J*_P,C_ = 128.6 Hz, P–*C*H_2_), 22.62 (d, ^2^*J*_P,C_ = 4.4 Hz, S–*C*H_2_), 22.60 (d, ^2^*J*_P,C_ = 3.8 Hz, S–*C*H_2_). ^31^P NMR (162 MHz, CDCl_3_, 25 °C): δ 30.7, 31.0. HRMS (ESI-Q-TOF) C_17_H_22_N_3_O_3_PS *m*/*z*: [M + H]^+^ calcd, 346.0878; found, 346.0885.

#### (*R*/*S*)-(2-((Bis(pyridin-2-ylmethyl)amino) (methoxy)phosphoryl)ethyl) Ethanethioate (**1d**)

^1^H NMR (500 MHz, CDCl_3_, 25 °C): δ 8.53 (ddd, *J* = 4.9, 1.8, 0.9 Hz, m-*H*_*Ar*_), 7.61 (ddd, *J* = 7.6, 7.6, 1.4 Hz, 2H, m-*H*_*Ar4*_), 7.29 (m, 2H, o-*H*_*Ar3*_), 7.15 (ddd, *J* = 7.5, 4.8, 1.1 Hz, 2H, p-*H*_*Ar5*_), 4.31 (d, *J* = 9.9 Hz, 4H, C*H*_*2*_), 3.67 (d, *J* = 11.1 Hz, 3H, P–OC*H*_*3*_), 3.17 (dt, *J* = 5.0, 0.9 Hz, S–C*H*_*2*_), 2.31 (s, 3H, C*H*_*3*_), 2.36–2.12 (m, 3H, P–C*H*_*2*_). ^13^C NMR (126 MHz, CDCl_3_, 25 °C): δ 195.5 (*C*=O), 157.9 (d, *J* = 2.3 Hz, ipso-*C*_*Ar2*_), 149.6 (m-*C*_*Ar6*_), 136.6 (m-*C*_*Ar4*_), 122.8 (o-*C*_*Ar3*_), 122.4 (p-*C*_*Ar5*_), 50.9 (d, ^2^*J*_P,C_ = 7.1 Hz, P–O*C*H_3_), 50.6 (d, ^2^*J*_P,C_ = 4.2 Hz, *C*H_2_), 30.7 (*C*H_3_), 27.9 (d, ^1^*J*_P,C_ = 128.0 Hz, P–*C*H_2_), 23.0 (d, *J* = 2.2 Hz, S–*C*H_2_). ^31^P NMR (162 MHz, CDCl_3_, 25 °C): δ 34.7. HRMS (ESI-Q-TOF) C_17_H_22_N_3_O_3_PS *m*/*z*: [M + H]^+^ calcd, 380.1198; found, 380.1209.

#### *S*-((*R*/*S*)-2-((*R*/*S*)-(2-(Ethoxy((pyridine-2-ylmethyl)amino)phosphoryl)-3-phenylpropyl) Ethanethioate (Mixture of Two Diastereoisomers) (**1e**)

^1^H NMR (500 MHz, DMSO-*d*_6_, 25 °C): δ 8.51–8.47 (m, 2H, 2 × m-*H*_*Ar6*_), 7.80–7.76 (m, 2H, 2 × m-*H*_*Ar4*_), 7.47–7.43 (m, 2H, 2 × o-*H*_*Ar3*_), 7.29–7.23 (m, 6H, 2 × p-*H*_*Ar5*_, 4 × m-*H*_*Ar*_), 7.21–7.18 (m, 2H, 2 × p-*H*_*Ar*_), 7.18–7.14 (m, 4H, 2 × o-*H*_*Ar*_), 5.30 (q, *J* = 7.3 Hz, 1H, N*H*), 5.27 (q, *J* = 7.2 Hz, 1H, N*H*), 4.17–4.09 (m, 2H, P–OC*H*_*2*_–CH_3_), 4.09–4.03 (m, 2H, P–OC*H*_*2*_–CH_3_), 3.97–3.88 (m, 2H, N–C*H*_*2*_), 3.87–3.79 (m, 2H, N–C*H*_*2*_), 3.14 (ddd, *J* = 17.9, 8.8, 4.8 Hz, 2H, S–C*H*_*2*_), 3.09 (ddd, *J* = 18.4, 9.0, 5.2 Hz, 2H, S–C*H*_*2*_), 3.06–2.96 (m, 2H, C*H*_*2*_), 2.95–2.86 (m, 2H, N–C*H*_*2*_^′^), 2.68–2.58 (m, 2H, C*H*_*2*_^′^), 2.34–2.25 (m, 2H, 2 × P–C*H*), 2.25 (s, 3H, C*H*_*3*_), 2.24 (s, 3H, C*H*_*3*_), 1.15 (t, *J* = 7.1 Hz, 3H, P–OCH_2_–C*H*_*3*_), 1.13 (t, *J* = 7.0 Hz, 3H, P–CH_2_–C*H*_*3*_). ^13^C NMR (126 MHz, DMSO-*d*_6_, 25 °C): δ 195.00 (*C*=O), 194.96 (*C*=O), 160.18 (d, ^3^*J*_P,C_ = 4.62 Hz, 2 × ipso-*C*_*Ar2*_), 148.69 (m-*C*_*Ar6*_), 148.68 (m-*C*_*Ar6*_), 139.39 (d, ^3^*J*_P,C_ = 11.0 Hz, ipso-*C*_*Ar*_), 139.20 (d, ^3^*J*_P,C_ = 12.0 Hz, ipso-*C*_*Ar*_), 136.69 (2 × m-*C*_*Ar4*_), 128.94 (o-*C*_*Ar*_), 128.93 (o-*C*_*Ar*_), 128.22 (m-*C*_*Ar*_), 128.20 (m-*C*_*Ar*_), 126.19 (p-*C*_*Ar*_), 126.15 (p-*C*_*Ar*_), 122.04 (p-*C*_*Ar5*_), 121.20 (o-*C*_*Ar3*_), 121.17 (o-*C*_*Ar3*_), 59.13 (d, ^2^*J*_P,C_ = 7.3 Hz, N–*C*H_2_), 59.08 (d, ^2^*J*_P,C_ = 6.5 Hz, N–*C*H_2_), 45.78 (d, ^2^*J*_P,C_ = 10.4 Hz, *C*H_2_), 38.78 (d, ^1^*J*_P,C_ = 127.0 Hz, P–*C*H), 38.57 (d, ^1^*J*_P,C_ = 127.5 Hz, P–*C*H), 33.61 (d, ^2^*J*_P,C_ = 2.5 Hz, *C*H_2_), 33.31 (d, ^2^*J*_P,C_ = 1.2 Hz, *C*H_2_), 30.39 (*C*H_3_), 27.61 (S–*C*H_2_), 27.48 (d, *J* = 1.6 Hz, *S*–*C*H_2_), 16.19 (d, ^3^*J*_P,C_ = 6.5 Hz, P–OCH_2_–*C*H_3_), 16.17 (d, ^3^*J*_P,C_ = 6.0 Hz, P–OCH_2_–*C*H_3_). ^31^P NMR (162 MHz, DMSO-*d*_6_, 25 °C): δ 33.28, 33.26. HRMS (ESI-Q-TOF) C_20_H_27_N_2_O_3_PS *m*/*z*: [M + H]^+^ calcd, 407.1558; found, 407.1519.

#### *S*-((*R*/*S*)-2-((*R*/*S*)-Ethoxy(methyl(pyridin-2-ylmethyl)amino)phosphoryl)-3-phenylpropyl) Ethanethioate (Mixture of Two Diastereoisomers) (**1f**)

^1^H NMR (500 MHz, DMSO-*d*_6_, 25 °C): δ 8.52 (ddd, *J* = 4.8, 1.7, 0.9 Hz, 1H, m-*H*_*Ar6*_), 8.49 (ddd, *J* = 5.0, 1.8, 1.0 Hz, 1H, m-*H*_*Ar6*_), 7.79 (ddd, *J* = 7.7, 7.5, 1.9 Hz, 1H, m-*H*_*Ar4*_), 7.77 (ddd, *J* = 7.7, 7.6, 1.9 Hz, 1H, m-*H*_*Ar4*_), 7.39 (ddd, *J* = 7.7, 2.1, 1.1 Hz, 1H, o-*H*_*Ar3*_), 7.30–7.25 (m, 6H, o-*H*_*Ar3*_, p-*H*_*Ar5*_), 7.25–7.18 (m, 6H), 4.35 (dd, *J* = 15.4, 8.0 Hz, 1H, P–OC*H*_*2*_–CH_3_), 4.21 (dd, *J* = 15.4, 8.2 Hz, 1H, P–OC*H*_*2*_–CH_3_), 4.11 (dd, *J* = 15.4, 9.3 Hz, 1H, P–OC*H*_*2*_–CH_3_), 4.02–3.90 (m, 2H, N–C*H*_*2*_), 3.90–3.79 (m, 2H, N–C*H*_*2*_), 3.69 (dd, *J* = 15.4, 9.5 Hz, 1H, P–C*H*_*2*_–CH_3_), 3.20 (ddd, *J* = 17.0, 13.7, 4.6 Hz, 1H, C*H*_*2*_), 3.12 (ddd, *J* = 18.4, 13.5, 5.0 Hz, 1H, S– C*H*_*2*_), 3.04–2.97 (m, 1H, C*H*_*2*_), 2.97–2.91 (m, 1H, S– C*H*_*2*_), 2.91–2.83 (m, 2H, C*H*_*2*_), 2.78–2.67 (m, 2H, S– C*H*_*2*_), 2.55 (d, *J* = 8.6 Hz, 3H, N–C*H*_*3*_), 2.54–2.51 (m, 2H, P–C*H*), 2.34 (d, *J* = 8.6 Hz, 3H, N–C*H*_*3*_), 2.29 (s, 3H, C*H*_*3*_), 2.29 (s, 3H, C*H*_*3*_), 1.19 (t, *J* = 7.1 Hz, 3H, P–OCH_2_–C*H*_*3*_), 1.10 (t, *J* = 7.1 Hz, 3H, P–OCH_2_–C*H*_*3*_). ^13^C NMR (126 MHz, DMSO-*d*_6_, 25 °C): δ 194.99 (*C*=O), 194.95 (*C*=O), 158.18 (d, ^3^*J*_P,C_ = 3.3 Hz, ipso-*C*_*Ar2*_), 158.15 (d, ^3^*J*_P,C_ = 2.7 Hz, ipso-*C*_*Ar2*_), 149.12 (m-*C*_*Ar6*_), 149.07 (m-*C*_*Ar6*_), 139.18 (d, ^3^*J*_P,C_ = 10.3 Hz, ipso-*C*_*Ar*_), 138.89 (d, ^3^*J*_P,C_ = 10.3 Hz, ipso-*C*_*Ar*_), 136.80 (m-*C*_*Ar4*_), 136.78 (m-*C*_*Ar4*_), 129.02 (o-*C*_*Ar*_), 128.89 (o-*C*_*Ar*_), 128.28 (m-*C*_*Ar*_), 128.16 (m-*C*_*Ar*_), 126.30 (p-*C*_*Ar*_), 126.17 (p-*C*_*Ar*_), 122.34 (p-*C*_*Ar5*_), 122.32 (p-*C*_*Ar5*_), 122.04 (o-*C*_*Ar3*_), 122.01 (o-*C*_*Ar3*_), 59.13 (d, ^2^*J*_P,C_ = 7.0 Hz, N–*C*H_2_), 59.02 (d, ^2^*J*_P,C_ = 7.0 Hz, N–*C*H_2_), 53.44 (d, ^2^*J*_P,C_ = 3.9 Hz, P–O*C*H_2_–CH_3_), 53.10 (d, ^2^*J*_P,C_ = 4.1 Hz, P–O*C*H_2_–CH_3_), 37.13 (d, ^1^*J*_P,C_ = 129.2 Hz, P–*C*H), 37.04 (d, ^1^*J*_P,C_ = 129.2 Hz, P–*C*H), 33.92 (d, ^2^*J*_P,C_ = 2.9 Hz, *C*H_2_), 33.19 (d, ^2^*J*_P,C_ = 4.3 Hz, *C*H_2_), 33.15 (d, ^2^*J*_P,C_ = 3.8 Hz, N–*C*H_3_), 32.84 (d, ^2^*J*_P,C_ = 4.2 Hz, N–*C*H_3_), 30.43 (*C*H_3_), 28.12 (d, ^2^*J*_P,C_ = 2.7 Hz, S–*C*H_2_), 27.27 (S–*C*H_2_), 16.07 (d, ^3^*J*_P,C_ = 6.2 Hz, P–OCH_2_–*C*H_3_), 15.93 (d, ^3^*J*_P,C_ = 6.3 Hz, P–OCH_2_–*C*H_3_). ^31^P NMR (162 MHz, DMSO-*d*_6_, 25 °C): δ 34.49, 34.18 HRMS (ESI-Q-TOF) C_17_H_22_N_3_O_3_PS *m*/*z*: [M + H]^+^ calcd, 393.1401; found, 393.1432.

#### Methyl (*R*)-2-(((*R*/*S*)-((*R*/*S*)-1-(Acetylthio)-3-phenylpropan-2-yl) (ethoxy)phosphoryl)amino)-2-phenylacetate (Mixture of Four Diastereoisomers) (Some of the Signals in ^1^H NMR and ^13^C NMR are Overlapping for Diastereoisomers) (**1g**)

^1^H NMR (500 MHz, DMSO-*d*_6_, 25 °C): δ 7.47–7.39 (m, 8H, *H*_*Ar*_), 7.39–7.35 (m, 7H, *H*_*Ar*_), 7.35–7.25 (m, 7H, *H*_*Ar*_), 7.25–7.13 (m, 12H, *H*_*Ar*_), 7.10–7.04 (m, 4H, *m*-*H*_*Ar*_), 7.02–6.98 (m, 2H, m-*H*_*Ar2*_), 5.92 (dd, *J* = 12.3, 10.7 Hz, 1H, N*H*), 5.85 (dd, *J* = 12.3, 10.6 Hz, 1H, N*H*), 5.82 (dd, *J* = 13.1, 10.5 Hz, 1H, N*H*), 5.79 (dd, *J* = 12.0, 11.8 Hz, 1H, N*H*) 5.07–4.97 (m, 4H, N–C*H*), 3.96–3.90 (m, 2H, P–OC*H*_*2*_–CH_3_), 3.90–3.83 (m, 2H, P–OC*H*_*2*_–CH_3_), 3.79–3.71 (m, 2H, P–OC*H*_*2*_–CH_3_), 3.71–3.64 (m, 2H, P–OC*H*_*2*_–CH_3_), 3.63 (s, 3H, OC*H*_*3*_), 3.63 (s, 3H, OC*H*_*3*_), 3.62 (s, 3H, OC*H*_*3*_), 3.62 (s, 3H, OC*H*_*3*_), 3.13–2.96 (m, 6H, 2 × C*H*_*2*,_ S–C*H*_*2*_), 2.96–2.87 (m, 4H, C*H*_*2*,_ S–C*H*_*2*_), 2.85–2.75 (m, 2H, C*H*_*2*_), 2.62–2.54 (m, 2H, S–C*H*_*2*_), 2.47–2.40 (m, 2H, S–C*H*_*2*_), 2.34–2.25 (m, 2H, 2 × P–C*H*), 2.24 (s, 3H, C*H*_*3*_), 2.24 (s, 3H, C*H*_*3*_), 2.20 (s, 3H, C*H*_*3*_), 2.19 (s, 3H, C*H*_*3*_), 2.16–2.08 (m, 2H, 2 × P–C*H*), 1.17 (t, *J* = 7.1 Hz, 3H, P–OCH_2_–C*H*_*3*_), 1.15 (t, *J* = 7.1 Hz, 3H, P–OCH_2_–C*H*_*3*_), 1.08 (t, *J* = 7.1 Hz, 3H, P–OCH_2_–C*H*_*3*_), 1.05 (t, *J* = 7.1 Hz, 3H, P–OCH_2_–C*H*_*3*_). ^13^C NMR (126 MHz, DMSO-*d*_6_, 25 °C): δ 194.97 (*C*=O), 194.92 (*C*=O), 194.89 (*C*=O), 194.86 (*C*=O), 172.58 (d, ^3^*J*_P,C_ = 8.9 Hz, ester *C*=O), 172.56 (d, ^3^*J*_P,C_ = 11.7 Hz, ester *C*=O), 172.46 (d, *J* = 15.4 Hz, ester *C*=O), 172.43 (d, ^3^*J*_P,C_ = 12.1 Hz, ester *C*=O), 139.35 (d, ^3^*J*_P,C_ = 11.9 Hz, ipso-*C*_*Ar*_), 139.28 (d, ^3^*J*_P,C_ = 11.5 Hz, ipso-*C*_*Ar*_), 139.19 (d, ^3^*J*_P,C_ = 10.9 Hz, ipso-*C*_*Ar*_), 139.16 (d, ^3^*J*_P,C_ = 11.3 Hz, ipso-*C*_*Ar*_), 139.10 (d, ^3^*J*_P,C_ = 13.7 Hz, ipso-*C*_*Ar2*_), 139.06 (d, ^3^*J*_P,C_ = 13.6 Hz, ipso-*C*_*Ar2*_), 138.96 (d, ^3^*J*_P,C_ = 12.1 Hz, ipso-*C*_*Ar2*_), 138.92 (d, ^3^*J*_P,C_ = 12.5 Hz, ipso-*C*_*Ar2*_), 128.92 (m-*C*_*Ar*_), 128.91 (m-*C*_*Ar*_), 128.87 (m-*C*_*Ar*_), 128.79 (m-*C*_*Ar*_), 128.59 (2 × m-*C*_*Ar2*_), 128.55 (2 × m-*C*_*Ar2*_), 128.26 (o-*C*_*Ar*_), 128.22 (o-*C*_*Ar*_), 128.20 (o-*C*_*Ar*_), 128.18 (o-*C*_*Ar*_), 127.90 (p-*C*_*Ar2*_), 127.89 (p-*C*_*Ar2*_), 127.87 (p-*C*_*Ar2*_), 127.83 (p-*C*_*Ar2*_), 127.24 (o-*C*_*Ar2*_), 127.19 (2 × o-*C*_*Ar2*_), 127.13 (o-*C*_*Ar2*_), 126.23 (p-*C*_*Ar*_), 126.21 (p-*C*_*Ar*_), 126.20 (p-*C*_*Ar*_), 126.15 (p-*C*_*Ar*_), 59.45 (d, ^2^*J*_P,C_ = 6.8 Hz, P–O*C*H_2_–CH_3_), 59.42 (d, ^2^*J*_P,C_ = 7.0 Hz, P–O*C*H_2_–CH_3_), 59.23 (d, ^2^*J*_P,C_ = 7.1 Hz, P–O*C*H_2_–CH_3_), 59.16 (d, ^2^*J*_P,C_ = 6.9 Hz, P–O*C*H_2_–CH_3_), 57.38 (d, ^2^*J*_P,C_ = 8.0 Hz, N–*C*H), 57.37 (d, ^2^*J*_P,C_ = 7.8 Hz, N–*C*H), 57.31 (d, ^2^*J*_P,C_ = 6.4 Hz, N–*C*H), 57.29 (d, ^2^*J*_P,C_ = 6.4 Hz, N–*C*H), 52.30 (O*C*H_3_), 52.29 (O*C*H_3_), 52.28 (O*C*H_3_), 52.26 (O*C*H_3_), 39.01 (d, ^1^*J*_P,C_ = 130.1 Hz, P–*C*H), 38.79 (d, ^1^*J*_P,C_ = 129.5 Hz, P–*C*H), 38.75 (d, ^1^*J*_P,C_ = 130.1 Hz, P–*C*H), 38.54 (d, ^1^*J*_P,C_ = 128.9 Hz, P–*C*H), 33.32 (d, ^2^*J*_P,C_ = 2.6 Hz, *C*H_2_), 33.27 (d, ^2^*J*_P,C_ = 2.5 Hz, *C*H_2_), 32.96 (d, ^2^*J*_P,C_ = 0.9 Hz, *C*H_2_), 32.90 (d, ^2^*J*_P,C_ = 1.0 Hz, *C*H_2_), 30.38 (2 × *C*H_3_), 30.33 (2 × *C*H_3_), 27.40 (S–*C*H_2_), 26.98 (S–*C*H_2_), 26.96 (S–*C*H_2_), 26.94, (S–*C*H_2_) 16.13 (d, ^2^*J*_P,C_ = 6.2 Hz, P–CH_2_–*C*H_3_), 16.10 (d, ^2^*J*_P,C_ = 6.4 Hz, P–CH_2_–*C*H_3_), 16.04 (d, ^2^*J*_P,C_ = 6.3 Hz, P–CH_2_–*C*H_3_), 16.01 (d, ^2^*J*_P,C_ = 6.3 Hz, P–CH_2_–*C*H_3_). ^31^P NMR (162 MHz, DMSO-*d*_6_, 25 °C): δ 32.14, 32.13, 32.12, 32.04. HRMS (ESI-Q-TOF) C_22_H_28_NO_5_PS *m*/*z*: [M + H]^+^ calcd, 450.1504, found, 450.1491.

#### *S*-((*R*/*S*)-2-((*R*/*S*)-(Bis(pyridin-2-ylmethyl)amino)(ethoxy)phosphoryl)-3-phenylpropyl) Ethanethioate (Mixture of Two Diastereoisomers) (Some of the Signals in ^1^H NMR are Overlapping for Diastereoisomers) (**1h**)

^1^H NMR (500 MHz, CDCl_3_, 25 °C): δ 8.51–8.46 (m, 4H, m-*H*_*AFr6*_), 7.71–7.63 (m, 4H, m-*H*_*Ar4*_), 7.49–7.45 (m, 2H, o-*H*_*Ar3*_), 7.46–7.41 (m, 2H, o-*H*_*Ar3*_), 7.32–7.27 (m, 4H, o-*H*_*Ar*_), 7.25–7.15 (m, 10H, m-*H*_*Ar*_, p-*H*_*Ar*_, p-*H*_*Ar5*_), 4.51 (dd, *J* = 15.5, 8.7 Hz, 2H, N–C*H*_*2*_^′^), 4.44 (dd, *J* = 15.5, 8.6 Hz, 2H, N–C*H*_*2*_), 4.34 (m, 2H, N–C*H*_*2*_^′^), 4.22 (m, 2H, N–C*H*_*2*_), 4.18 (m, 1H, P–OC*H*_*2*_–CH_3_), 4.09 (m, 1H, P–OC*H*_*2*_–CH_3_), 4.00–3.87 (m, 2H, P–OC*H*_*2*_–CH_3_), 3.34–3.15 (m, 5H, 2 × C*H*_*2*_, 3 × S– C*H*_*2*_), 3.08 (ddd, *J* = 13.6, 10.7, 7.6 Hz, 1H, S–C*H*_*2*_), 2.91 (ddd, *J* = 14.5, 8.0 Hz, 1H, C*H*_*2*_), 2.84 (m, 1H, C*H*_*2*_), 2.81 (m, 1H, P–C*H*), 2.75 (m, 1H, P–C*H*), 2.27 (s, 6H, 2 × C*H*_*3*_), 1.27 (t, *J* = 7.1 Hz, 3H, P–OCH_2_–C*H*_*3*_), 1.14 (t, *J* = 7.1 Hz, 3H, P–OCH_2_–C*H*_*3*_). ^13^C NMR (126 MHz, CDCl_3_, 25 °C): δ 195.48 (*C*=O), 195.41 (*C*=O), 157.65 (d, ^3^*J*_P,C_ = 11.6 Hz, ipso-*C*_*Ar2*_), 148.37 (m-*C*_*Ar6*_), 148.18 (m-*C*_*Ar6*_), 139.53 (d, ^3^*J*_P,C_ = 10.4 Hz, ipso-*C*_*Ar*_), 138.97 (d, *J* = 14.2 Hz, ipso-*C*_*Ar*_), 137.63 (m-*C*_*Ar4*_), 137.62 (m-*C*_*Ar4*_), 129.35 (m-*C*_*Ar*_), 129.32 (m-*C*_*Ar*_), 128.58 (o-*C*_*Ar*_), 128.42 (o-*C*_*Ar*_), 126.68 (p-*C*_*Ar*_), 126.46 (p-*C*_*Ar*_), 124.12 (o-*C*_*Ar3*_), 124.09 (o-*C*_*Ar3*_), 122.72 (p-*C*_*Ar5*_), 122.73 (p-*C*_*Ar5*_), 60.51 (d, ^2^*J*_P,C_ = 7.3 Hz, P–O*C*H_2_–CH_3_), 60.31 (d, ^2^*J*_P,C_ = 7.2 Hz, P–O*C*H_2_–CH_3_), 51.12 (d, *J* = 4.1 Hz, N–*C*H_2_), 50.88 (d, ^2^*J*_P,C_ = 4.1 Hz, N–*C*H_2_), 39.07 (d, ^1^*J*_P,C_ = 129.2 Hz, P–*C*H), 38.92 (d, ^1^*J*_P,C_ = 130.2 Hz, P–*C*H), 34.56 (d, ^2^*J*_P,C_ = 3.0 Hz, *C*H_2_), 33.39 (*C*H_2_), 30.66 (*C*H_3_), 30.63 (*C*H_3_), 28.12 (d, ^2^*J*_P,C_ = 3.6 Hz, S–*C*H_2_), 28.03 (S–*C*H_2_), 16.27 (d, ^3^*J*_P,C_ = 7.0 Hz, P–OCH_2_–*C*H_3_), 16.10 (d, *J* = 7.2 Hz, P–OCH_2_–*C*H_3_). ^31^P NMR (162 MHz, CDCl_3_, 25 °C): δ 35.36, 35.16. HRMS (ESI-Q-TOF) C_25_H_30_N_3_O_3_PS *m*/*z*: [M + H]^+^ calcd, 483.1745, found, 484.1824.

### NDM-1 Backbone Assignment

Protein NMR spectra were recorded on a Bruker 800 MHz spectrometer at 310 K using a 3 mm TCI cryogenic probe. NMR samples (0.5 mM ^15^N,^13^C-labeled NDM-1) were prepared in 20 mM K_3_PO_4_ in 90% H_2_O/10% D_2_O at pH 7.0. For sequential backbone assignments, 2D ^1^H,^15^N HSQC, and the 3D experiments HNCA (hncagpwg3d),^36^ HN(CO)CA (hncocagpwg3d),^36^ HNCACB (hncacbgpwg3d),^37^ HN(CO)CACB (hncocacbgpwg3d),^38^ HNCO (hncogpwg3d),^36^ and HN(CA)CO (hncacogpwg3d)^39^ were acquired. Data obtained with NUS were processed using qMDD software,^40^ further processed with NMRPipe,^41^ and analyzed with software CcpNMR Analysis v 2.4.1.^42,43^ The NMR data have been deposited into the Biological Magnetic Resonance Bank with BMRB ID 50945.

### ^1^H–^15^N HSQC Titration Experiments

For the titrations, two ^15^N-labeled NDM-1 (0.25 mM) batches were prepared. Ligands (25 mM) **1b** and **1d** were prepared in the same buffer as the protein (20 mM KPO_4_, 0.1 mM ZnCl_2_, pH 7.0). ^1^H,^15^N HSQC spectra were acquired with 128 × 1024 complex points (F_1_ × F_2_) and spectral width of 9090 × 2740 Hz on ^15^N-labeled NDM-1 and with every titration step up to a 1:10 ratio between protein/ligand. All experiments were recorded on a Bruker 600 MHz spectrometer at 310 K equipped with a 5 mm TCI cryogenic probe. The NMR data were processed on MestReNova software with the Mnova binding plugin. The weighted average CSPs for the backbone amides were calculated from the observed chemical shift differences in the proton and nitrogen dimensions using the equation (chemical shift scaling factors: *F*_H_ = 1, *F*_N_ = 0.156): CSP = Δδ_1H,15N_ = √((1/*F*_H_ × Δδ(^1^H))^2^ + ((1/*F*_N_ × Δδ(^15^N))^2^).

### 3D ^15^N-Filtrated HSQC-NOESY

A mixture of NDM-1 (0.25 mM) and ligand **1d** in a 1:8 ratio was prepared in the buffer 20 mM KPO_4_ and 0.1 mM ZnCl_2_ at pH 7.0. 3D ^15^N-filtrated HSQC-NOESY spectra were acquired with 64 × 128 × 4096 complex points (F1 × F2 × F3) and spectral widths of 2740 × 9090 × 9090 Hz. Spectra were recorded on a Bruker 600 MHz spectrometer at 310 K equipped with a 5 mm TCI cryogenic probe. The NMR data were processed using software Topspin.

### Dose Rate Inhibition Studies for IC_50_ Determination

The inhibitory activity (IC_50_) of **1a–h** was studied against the MBL enzymes NDM-1, GIM-1, and VIM-2. The buffer used for the studies contained 50 mM HEPES at pH 7.2, 10 μM ZnSO_4_, 2.5% DMSO, and 0.4 mg/mL bovine serum albumin, used as a prevention of protein unfolding and loss of activity. The enzyme concentration of NDM-1 was 10 nM, of GIM-1 1 nM, and of VIM-2 100 pM. The inhibitors were dissolved in 100% DMSO, and a twofold dilution series was made with a final 2.5% DMSO in the assay. The highest inhibitor concentration was 800 μM. The reporter substrate for VIM-2 and GIM-1 was nitrocefin, while for NDM-1 imipenem, and their absorbance (concentration) was followed at the wavelengths 482 and 300 nm, respectively. L-Captopril and EDTA-Na were used as positive and water and DMSO as negative controls. Measurements were read for 30 min at 298 K.

### Cytotoxicity Assay

The cytotoxicity of phosphonamidate monoesters was evaluated against HeLa cells (ATCC-CCL-2), which were maintained in Dulbecco’s modified Eagle medium (Thermo Fisher Scientific) supplemented with 10% (v/v) fetal bovine serum (Thermo Fisher Scientific), penicillin (100 units/mL), and streptomycin (100 μg/mL, both from Sigma) at 37 °C, 5% CO_2_. Cells were seeded in 96-well plates (Corning, Merck) at 20 × 10^3^ cells/well and incubated for 24 h (37 °C, 5% CO_2_). Stock solutions of the compounds were prepared at 150 mM in DMSO (Sigma), and the cells were exposed to a serial dilution and incubated for 24 h. Subsequently, the cells were washed twice with culture medium, and the PrestoBlue reagent (Thermo Fischer Scientific) was directly added to the cells for cell viability determination (1:10 dilution in culture medium). Following recommended incubation times (manufacturer’s instructions), fluorescence was measured using a Spark plate reader (Tecan, Austria) at Ex/Em 560/590 nm and corrected for background fluorescence by including control wells containing only cell culture media (no cells). Data analysis was performed using GraphPad Prism 9.0.0 (GraphPad Software, USA). At least two independent experiments were performed.

### Minimum Inhibitory Concentration Studies

The in vitro susceptibility of meropenem in combination with the different compounds was evaluated against *E. coli* expressing NDM-1 in a broth microdilution assay. A 96-well plate was prepared with the compounds in a twofold dilution series (final concentrations 1024–0.5 mg/L) in Muller Hinton II broth (MHB-II) in combination with 2, 4, or 8 mg/L meropenem. EDTA (100 μM) was used in combination with meropenem as control for inhibition of NDM-1. Three–five colonies of *E. coli* ATCC 25922 NDM-1 were suspended in 0.90% saline solution and adjusted to the 0.5 McFarland turbidity standard. The bacterial suspension was diluted in MHB-II and adjusted to a final inoculum of 5 × 105 CFU/mL. Plates were incubated at 37 °C, and a minimum inhibitory concentration (MIC mg/L) was read after 20 h..

## Supporting Information Available

Dose–response graphs for enzyme assay, ^1^H–^15^N HSQC, 2D ^15^N-filtered NOESY for NDM-1, and **1d**, sequence of pET27b(+) used for the expression, and NMR spectra of synthesized compounds. (PDF)

## Abbreviation

NDM: New Dehli metallo-β-lactamase
