# Supplementary material for: Metallo-β-Lactamase Inhibitor Phosphonamidate Monoesters
Source: ACS Omega. 2022 Jan 25;7(5):4550–62. doi: 10.1021/acsomega.1c06527 (PMC8830069; doi:10.1021/acsomega.1c06527)
Supplement: Supplementary file 1 — ao1c06527_si_001.pdf [file ao1c06527_si_001.pdf]

Supporting Information

Metallo-β-lactamase inhibitor phosphonamidate monoesters

Katarzyna Palica,[†] Manuela Vorácová,[†] Susann Skagseth,[‡] Anna Andersson Rasmussen,[†] Lisa Allander,[±] Madlen Hubert,[|] Linus Sandegren,[±] Hanna-Kirsti Schrøder Leiros,[‡] Hanna Andersson,[*,†] and Máté Erdélyi[*,†]

[†] Department of Chemistry - BMC, Organic Chemistry; Uppsala University, Husargatan 3, 752 37 Uppsala, Sweden

[‡] The Norwegian Structural Biology Centre (NorStruct), Department of Chemistry, Faculty of Science and Technology, UiT The Arctic University of Norway, N-9037 Tromsø, Norway

[±] Department of Medical Biochemistry and Microbiology – BMC, Uppsala University, Husargatan 3, 752 37 Uppsala, Sweden

[|] Department of Pharmacy – BMC, Uppsala University, Husargatan 3, 752 37 Uppsala, Sweden

Table of contents

1. Dose-response graphs 3
3. MIC studies for NDM-1 6
4. Cytotoxicity assay 7
5. NMR assignment of NDM-1, chemical shift titration and NOE 8
6. The protein sequence of NDM-1 15
7. NMR spectra of the synthesized compounds 19

The protein NMR data has been deposited to the Biological Magnetic Resonance Bank with BMRB ID 50945. The original NMR FIDs for all compounds are available free of charge on Zenodo as DOI:10.5281/zenodo.4773990.

1. Dose-response graphs

Table S1. The dose-response graphs.

**Inhibitors**	**NDM-1**	**GIM-1**	**VIM-2**
**1a**	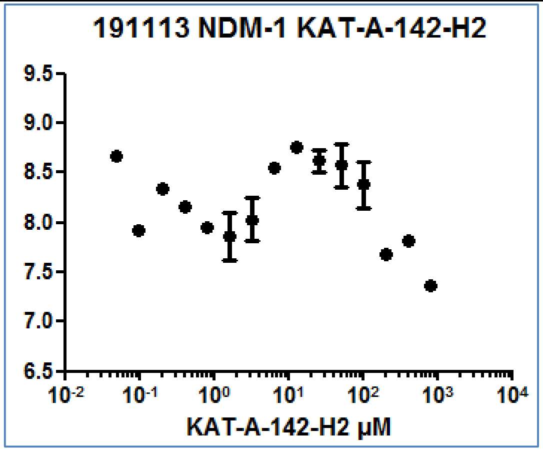	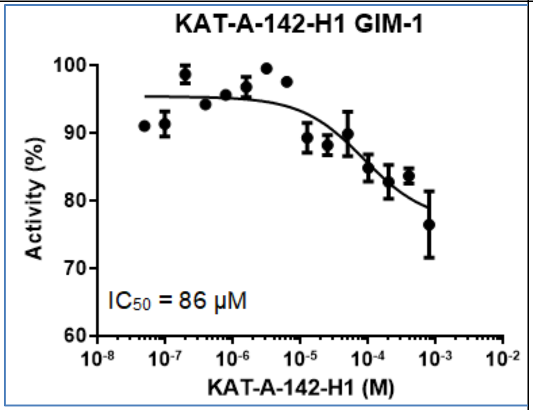	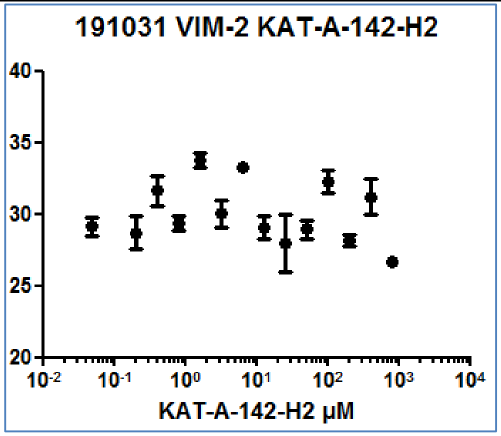
**1b**	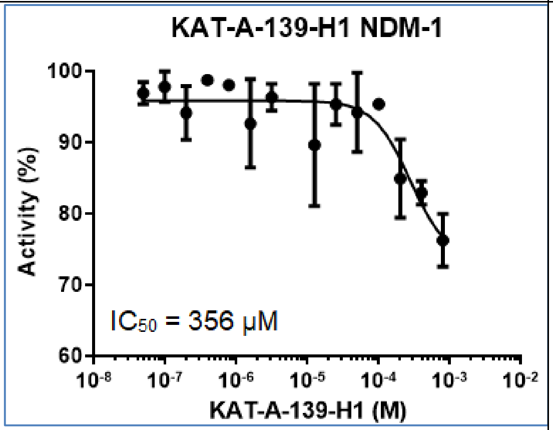	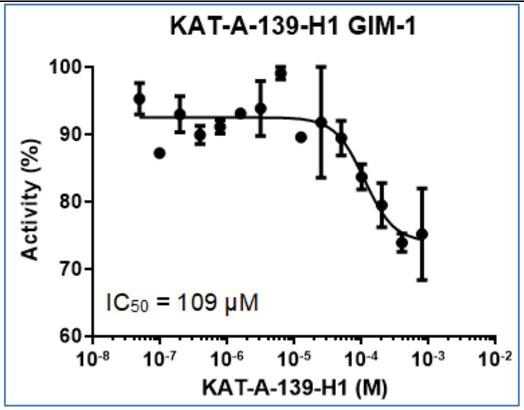	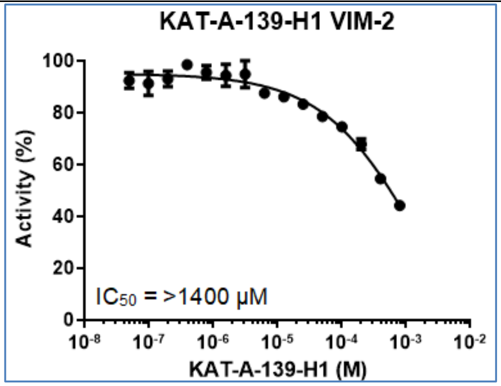

**1c**	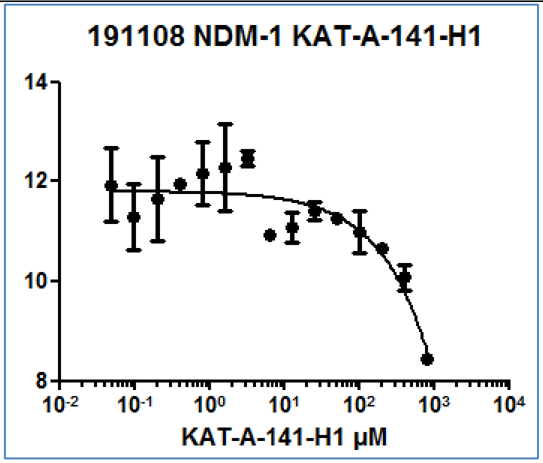191108 NDM-1 KAT-A-141-H1<br>KAT-A-141-H1 µM	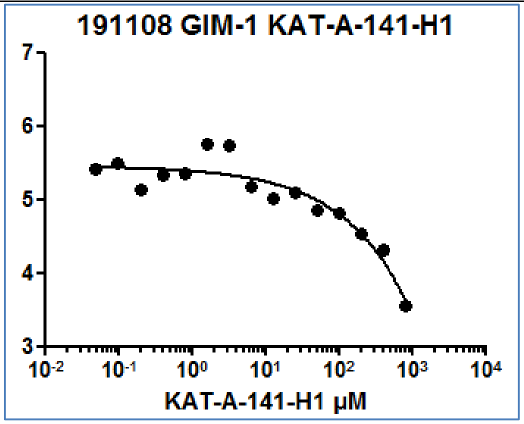191108 GIM-1 KAT-A-141-H1<br>KAT-A-141-H1 µM	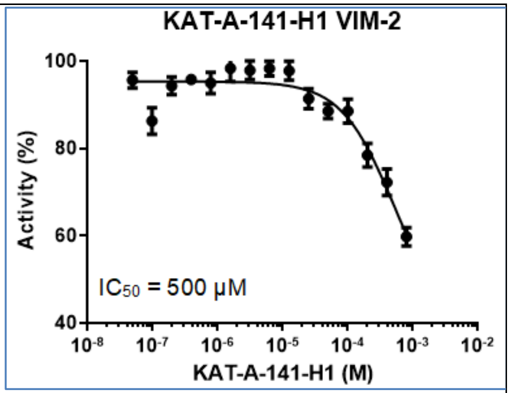KAT-A-141-H1 VIM-2<br>Activity (%)<br>IC50 = 500 µM<br>KAT-A-141-H1 (M)
**1d**	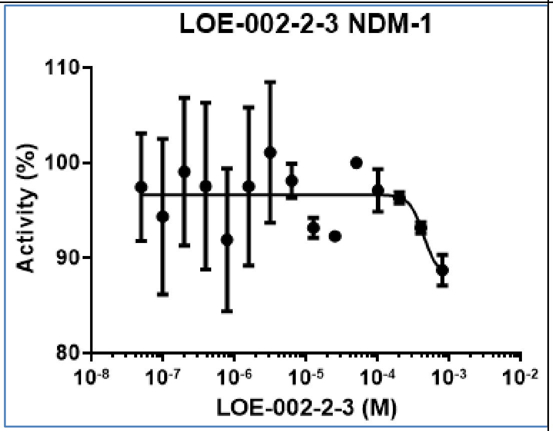LOE-002-2-3 NDM-1<br>Activity (%)<br>LOE-002-2-3 (M)	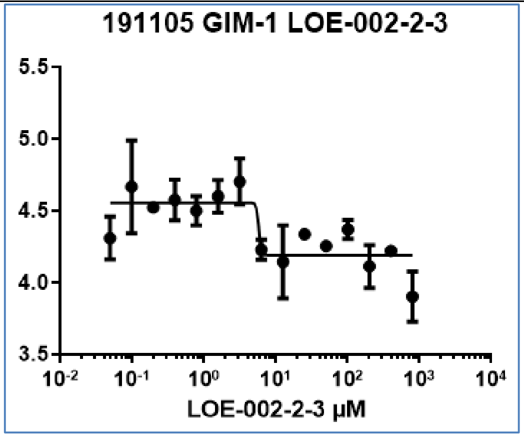191105 GIM-1 LOE-002-2-3<br>LOE-002-2-3 µM	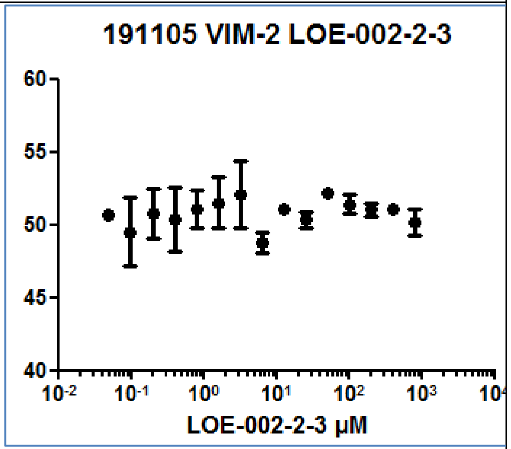191105 VIM-2 LOE-002-2-3<br>LOE-002-2-3 µM

2

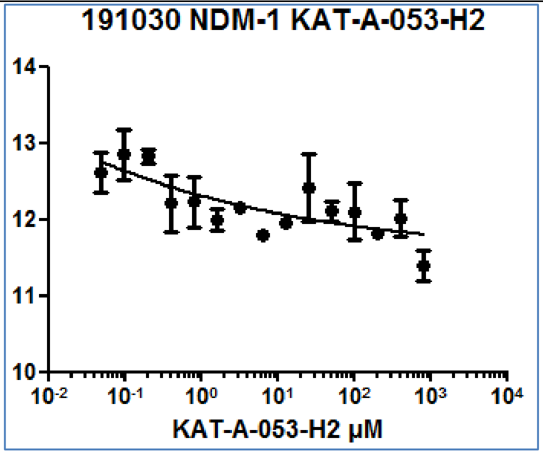
191030 NDM-1 KAT-A-053-H2
14
13
12
11
10
10^{-2} 10^{-1} 10^{0} 10^{1} 10^{2} 10^{3} 10^{4}
KAT-A-053-H2 µM

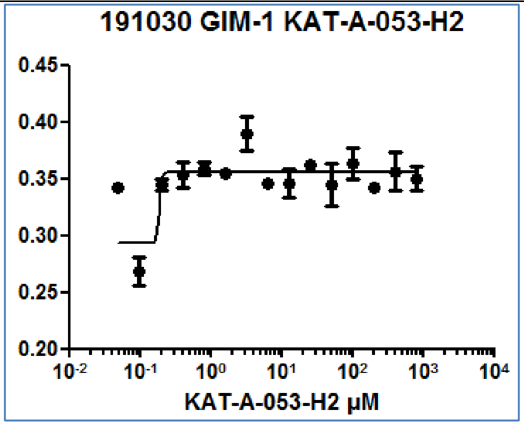
191030 GIM-1 KAT-A-053-H2
0.45
0.40
0.35
0.30
0.25
0.20
10^{-2} 10^{-1} 10^{0} 10^{1} 10^{2} 10^{3} 10^{4}
KAT-A-053-H2 µM

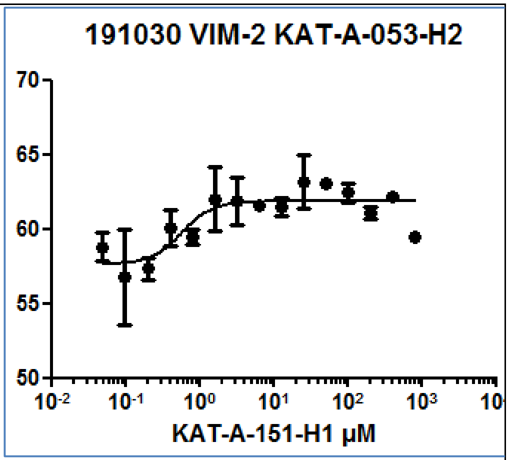
191030 VIM-2 KAT-A-053-H2
70
65
60
55
50
10^{-2} 10^{-1} 10^{0} 10^{1} 10^{2} 10^{3}
KAT-A-151-H1 µM

1e

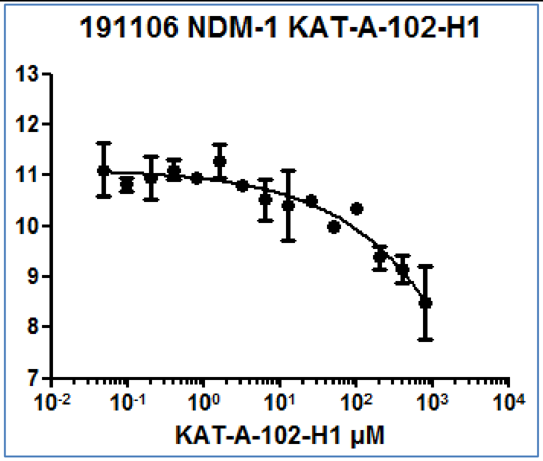
191106 NDM-1 KAT-A-102-H1
13
12
11
10
9
8
7
10^{-2} 10^{-1} 10^{0} 10^{1} 10^{2} 10^{3} 10^{4}
KAT-A-102-H1 µM

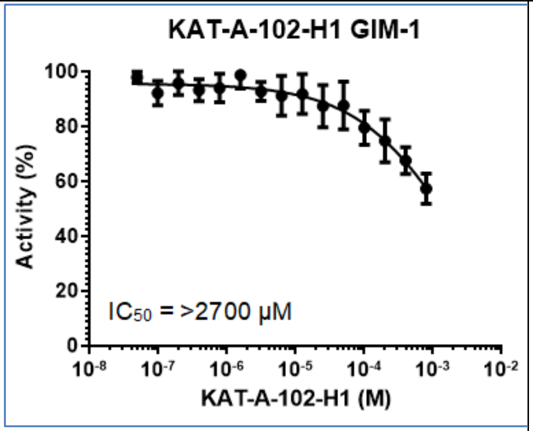
KAT-A-102-H1 GIM-1
Activity (%)
100
80
60
40
20
0
IC_{50} = >2700 µM
10^{-8} 10^{-7} 10^{-6} 10^{-5} 10^{-4} 10^{-3} 10^{-2}
KAT-A-102-H1 (M)

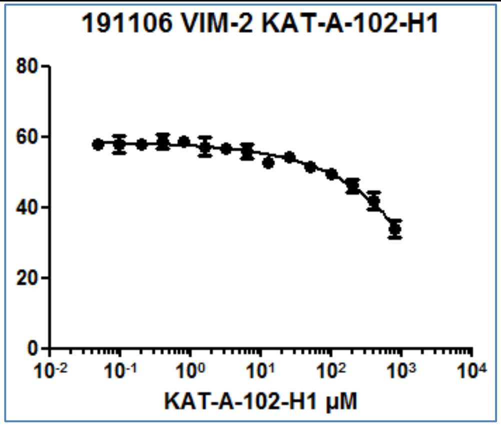
191106 VIM-2 KAT-A-102-H1
80
60
40
20
0
10^{-2} 10^{-1} 10^{0} 10^{1} 10^{2} 10^{3} 10^{4}
KAT-A-102-H1 µM

3. MIC studies for NDM-1

Table S2. Susceptibility assay with E. coli ATCC 25922 NDM-1

**Compound**	Minimum inhibitory concentration (mg/L)			
	Meropenem:compound ratio			Meropenem
No compound				32
EDTA 100 μM				< 2
**1c**	8:1024	4:1024	2:1024	
**1b**	8:1024	4:1024	2:1024	
**1d**	8:1024	4:1024	> 2:1024	
**1a**	8:1024	4:1024	2:1024	
**1h**	8:1024	4:1024	2:1024	

*__1e__, **1g** and **1f** could not be dissolved in MIC assay.

4. Cytotoxicity assay

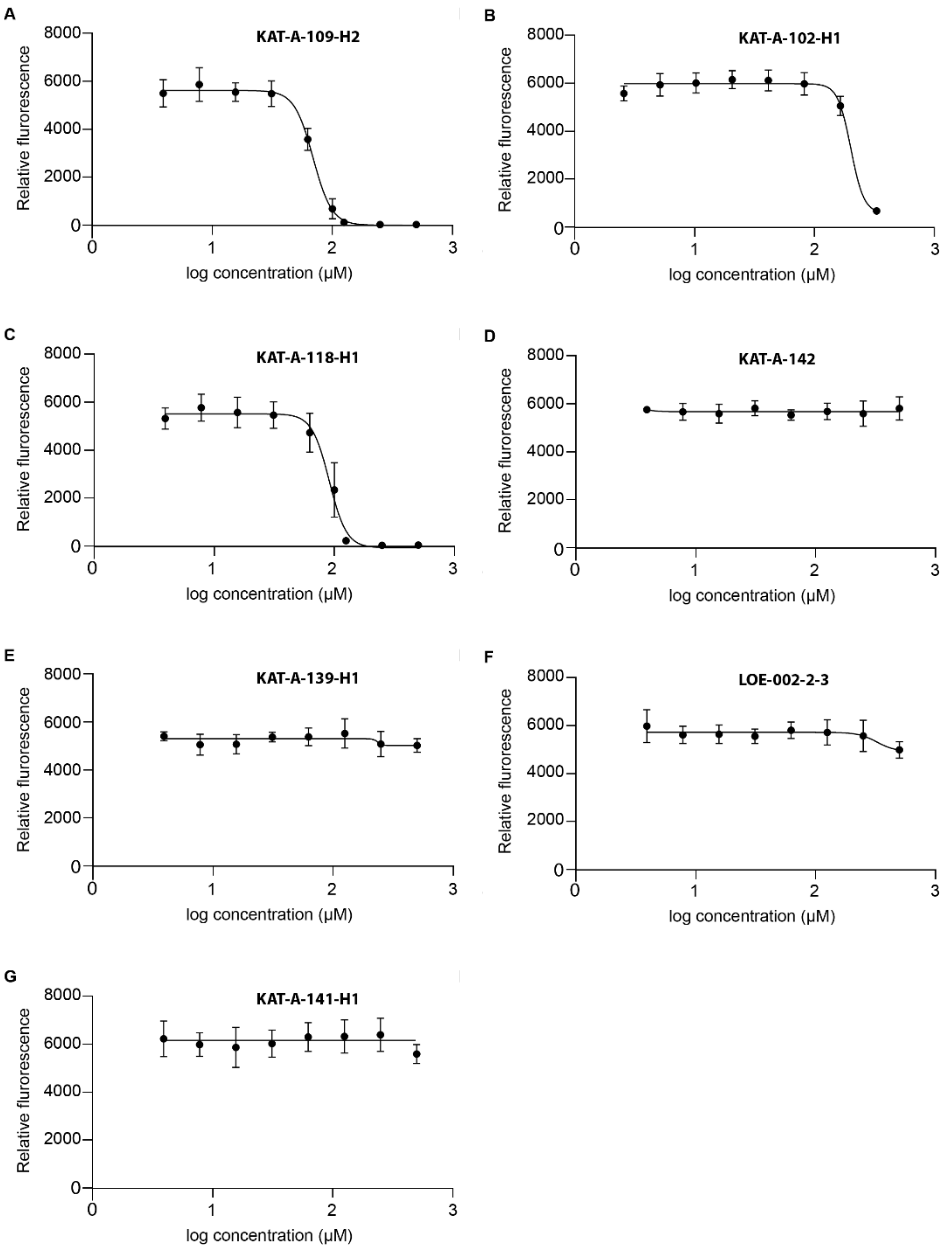

Figure S1. Cytotoxicity dose-response curves of phosphonamidate monoesters as determined in HeLa cells. The cells were treated with the compounds at the indicated concentrations, and cell viability was measured after 24 hours incubation. The following IC_{50} values were determined to be (A) 69.2 µM (SD = 1.0) for KAT-A-109-H2, (B) 201.4 µM (SD = 4.4) for KAT-A-102-H1 and (C) 90.7 µM (SD = 1.0) for KAT-A-118-H1. All remaining compounds (D-H) had IC_{50} values above 500 µM. Data is shown as mean ± SD of at least two independent experiments.

*A **1h**, B **1e**, C **1f**, D **1a**, E **1b**, F **1d**, G **1c**.

5. NMR assignment of NDM-1, chemical shift titration and NOE

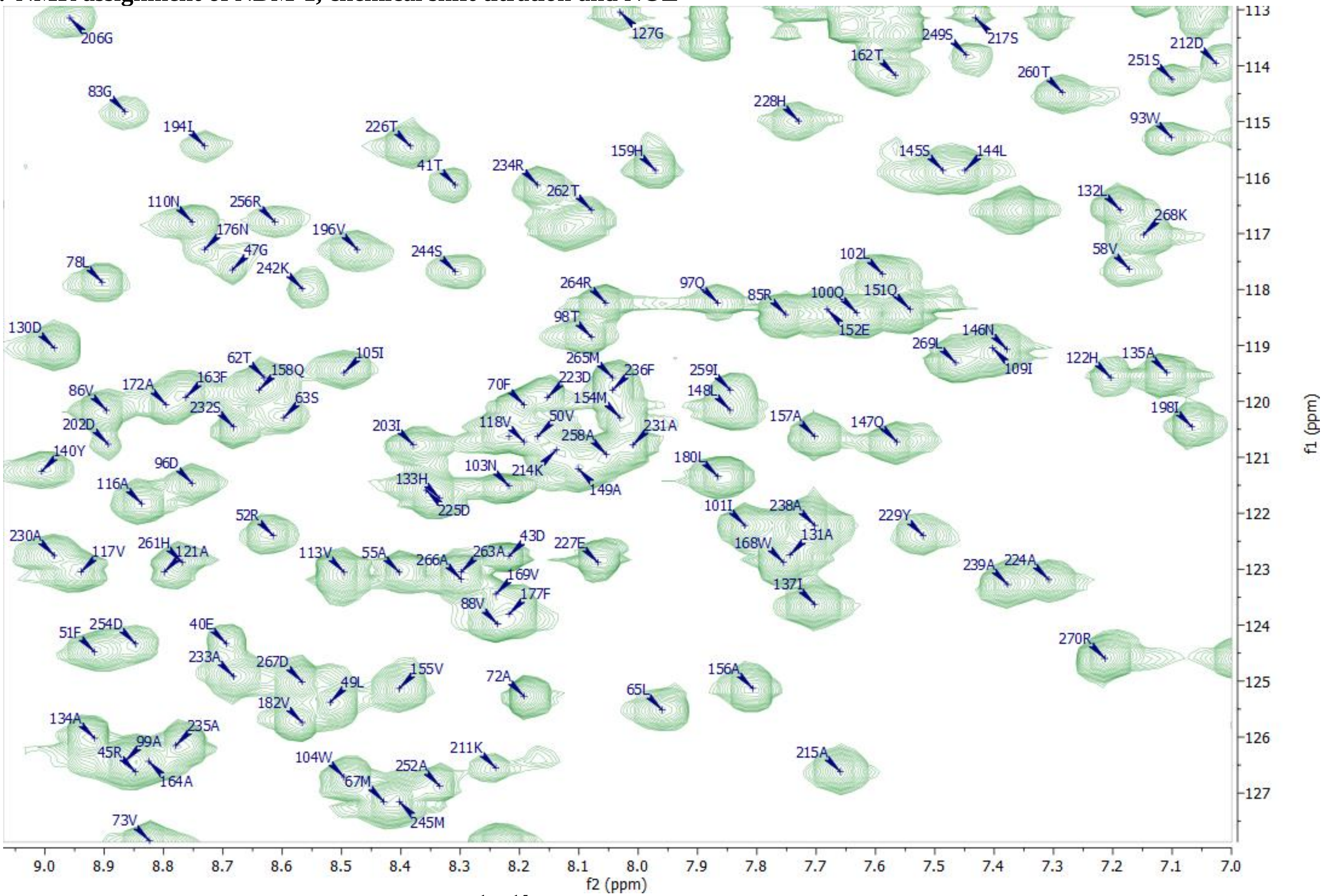

Figure S2. Expansion of the overlapping area of $^{1}H,^{15}N$ HSQC with assignment of chemical shift of NDM-1

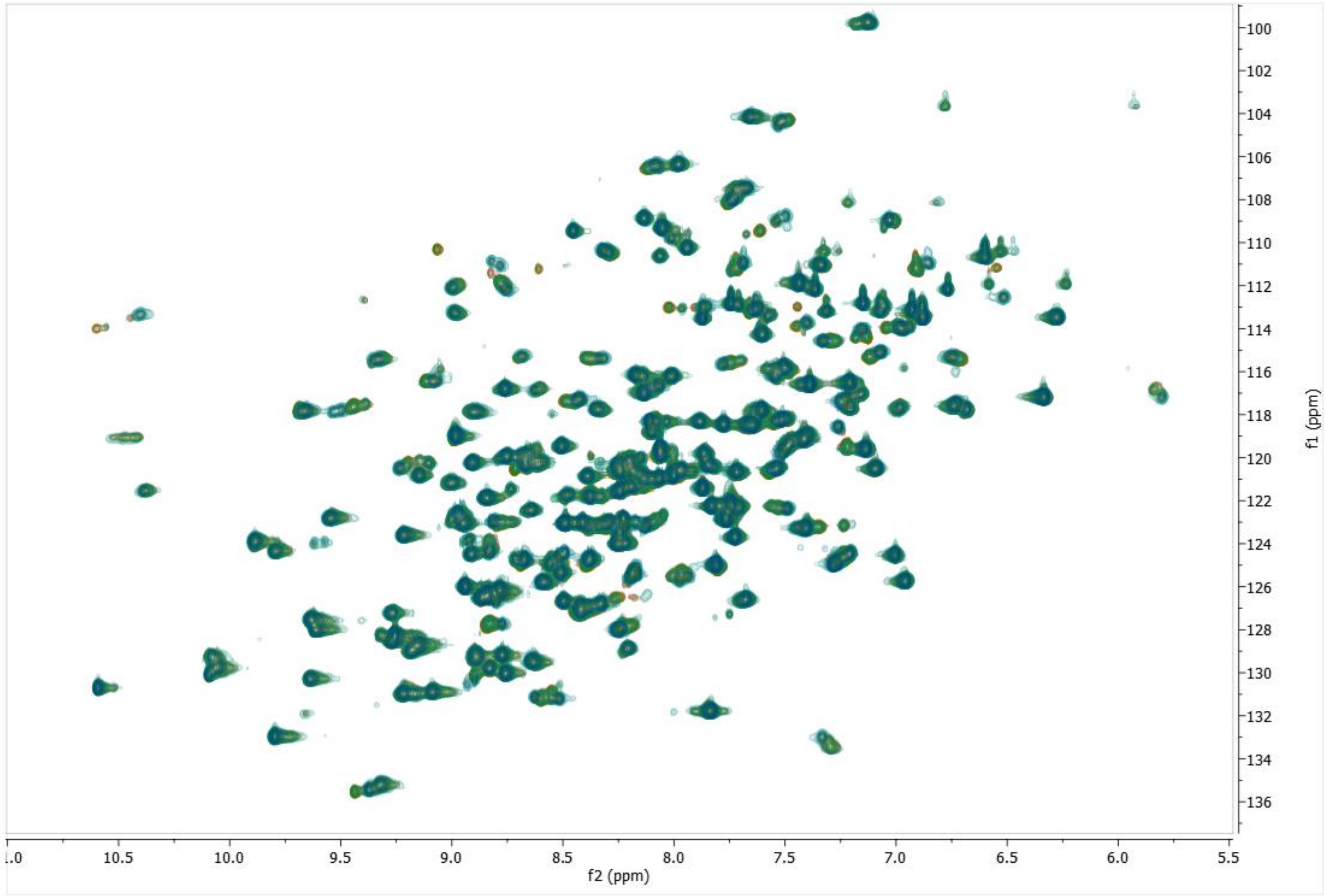

Figure S3. Superposition of the full ^{1}H,^{15}N HSQC spectrum of free NDM-1 (titration step 0 in red), and NDM-1 bound to **1d** (last step 10 eq blue)

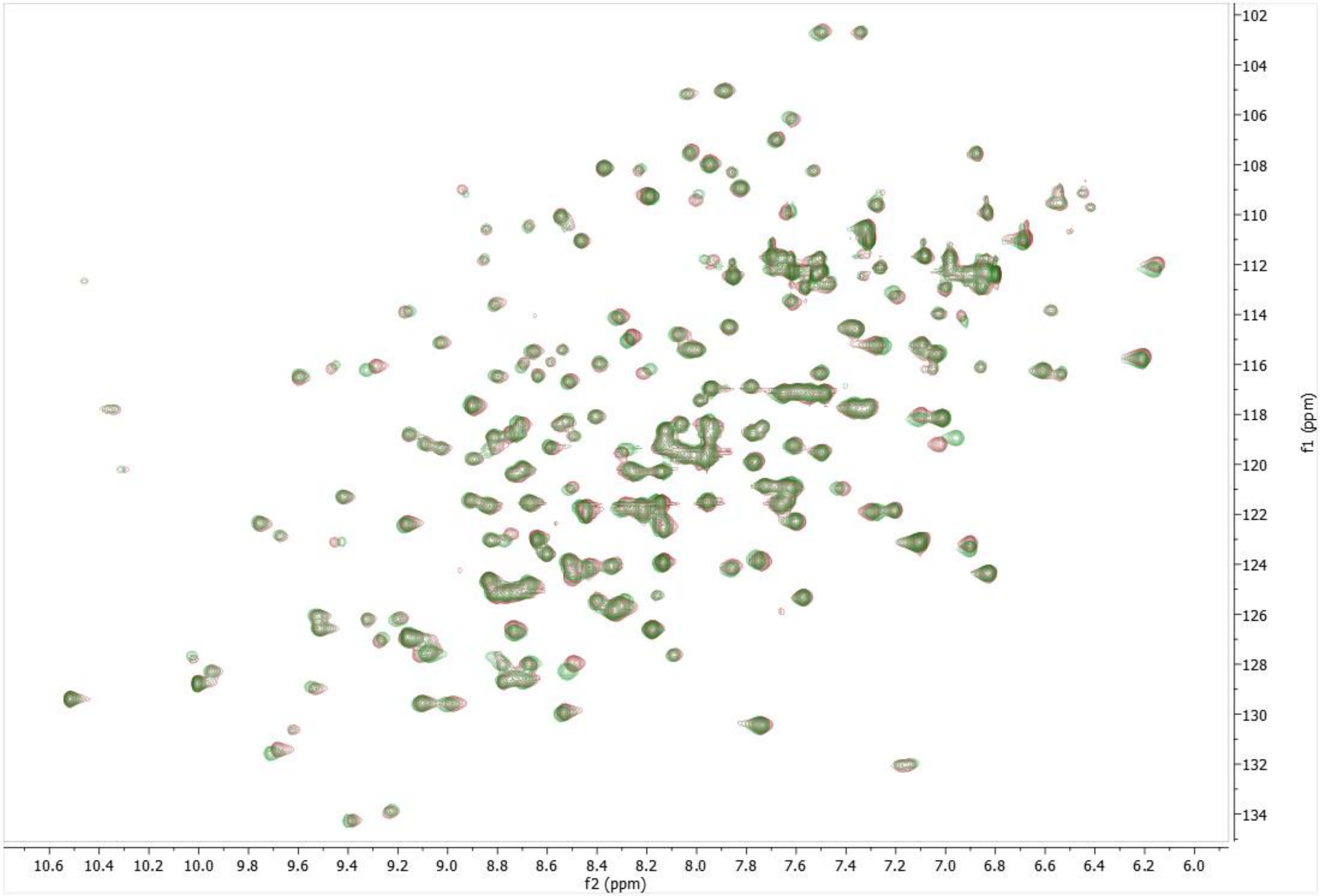

Figure S4. $^{1}H,^{15}N$ HSQC of NDM-1 (green) with 5% EtOH (red).

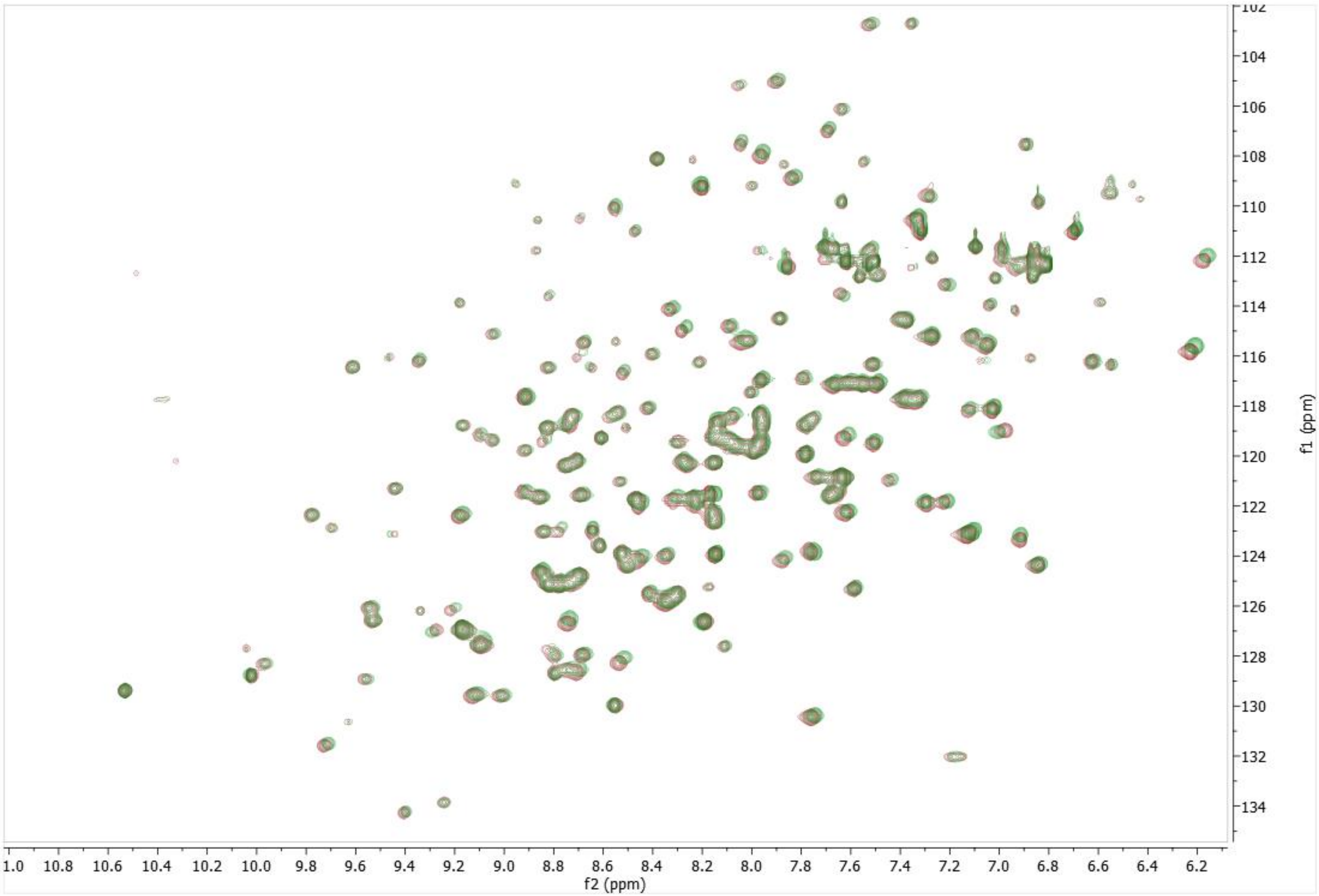

Figure S5. ^{1}H-^{15}N HSQC of NDM-1 (red) with 5% DMSO (green).

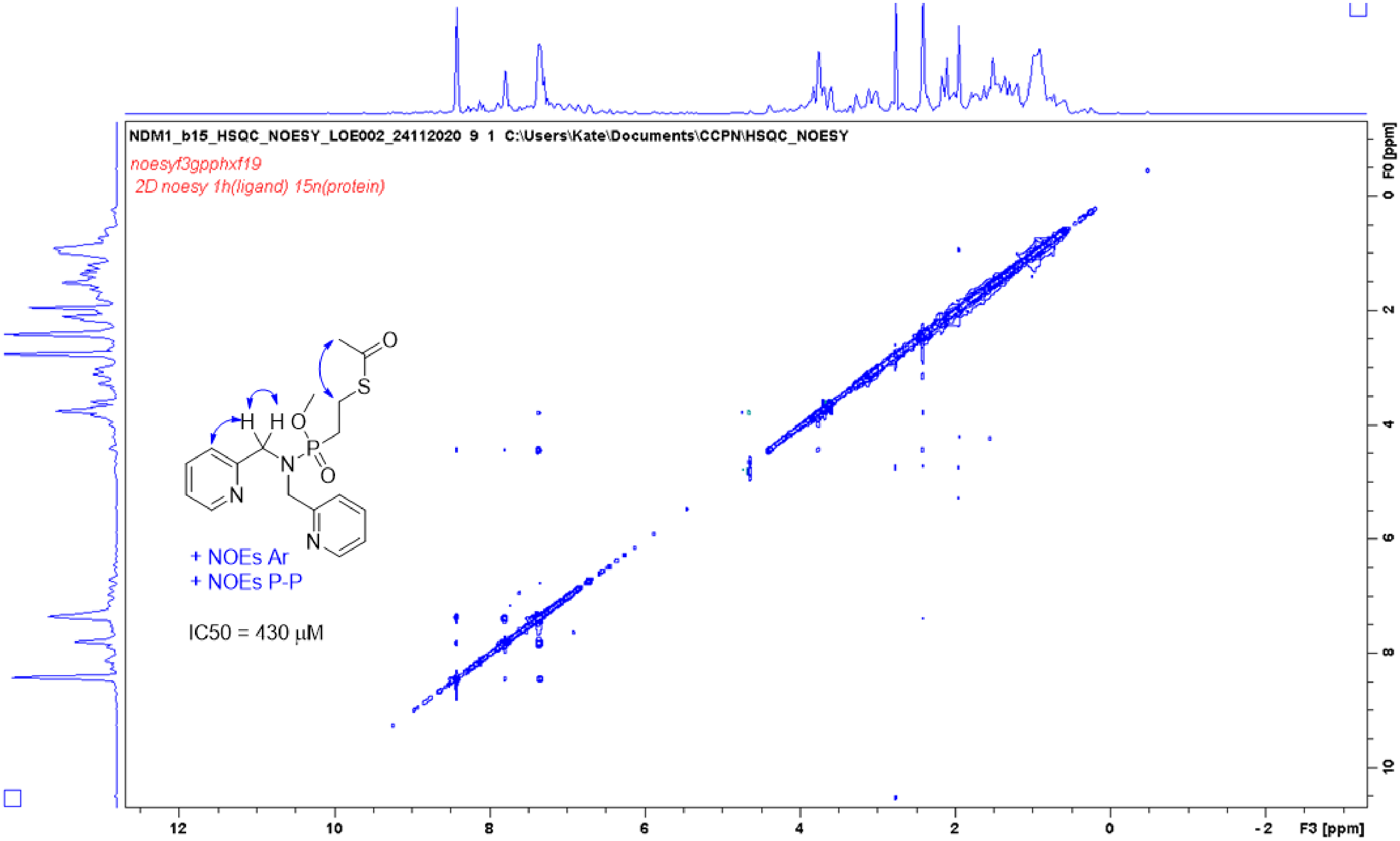

Figure S6. 2D ^{15}N-filtered NOESY for NDM-1 with **1d** (8 eq).

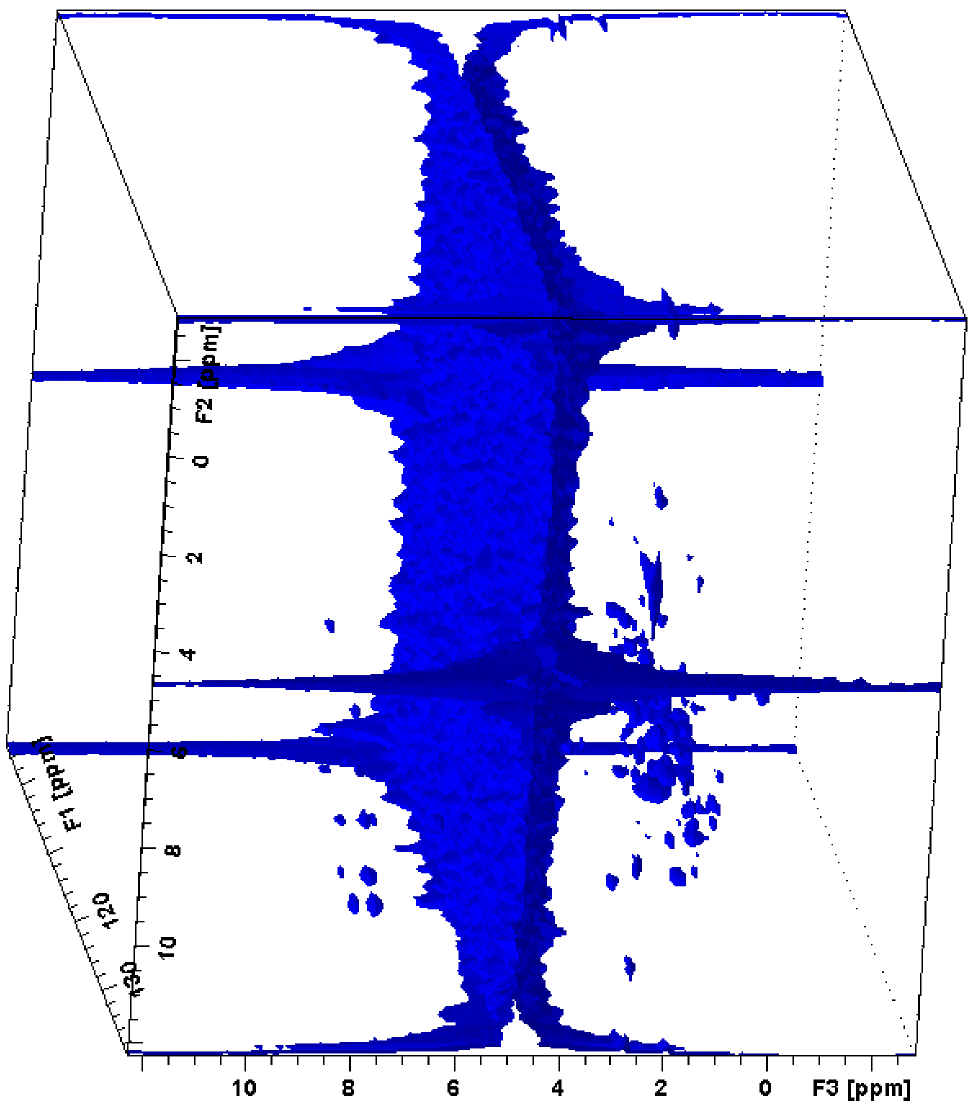

Figure S7. 3D ^{15}N-filtred HSQC-NOESY in 3D cube mode for NDM-1 and **1d** (8eq). Dimensions: F1 = chemical shift of ^{15}N protein; F2 = chemical shift of ^{1}H protein, F3 = chemical shift of ^{1}H ligand.

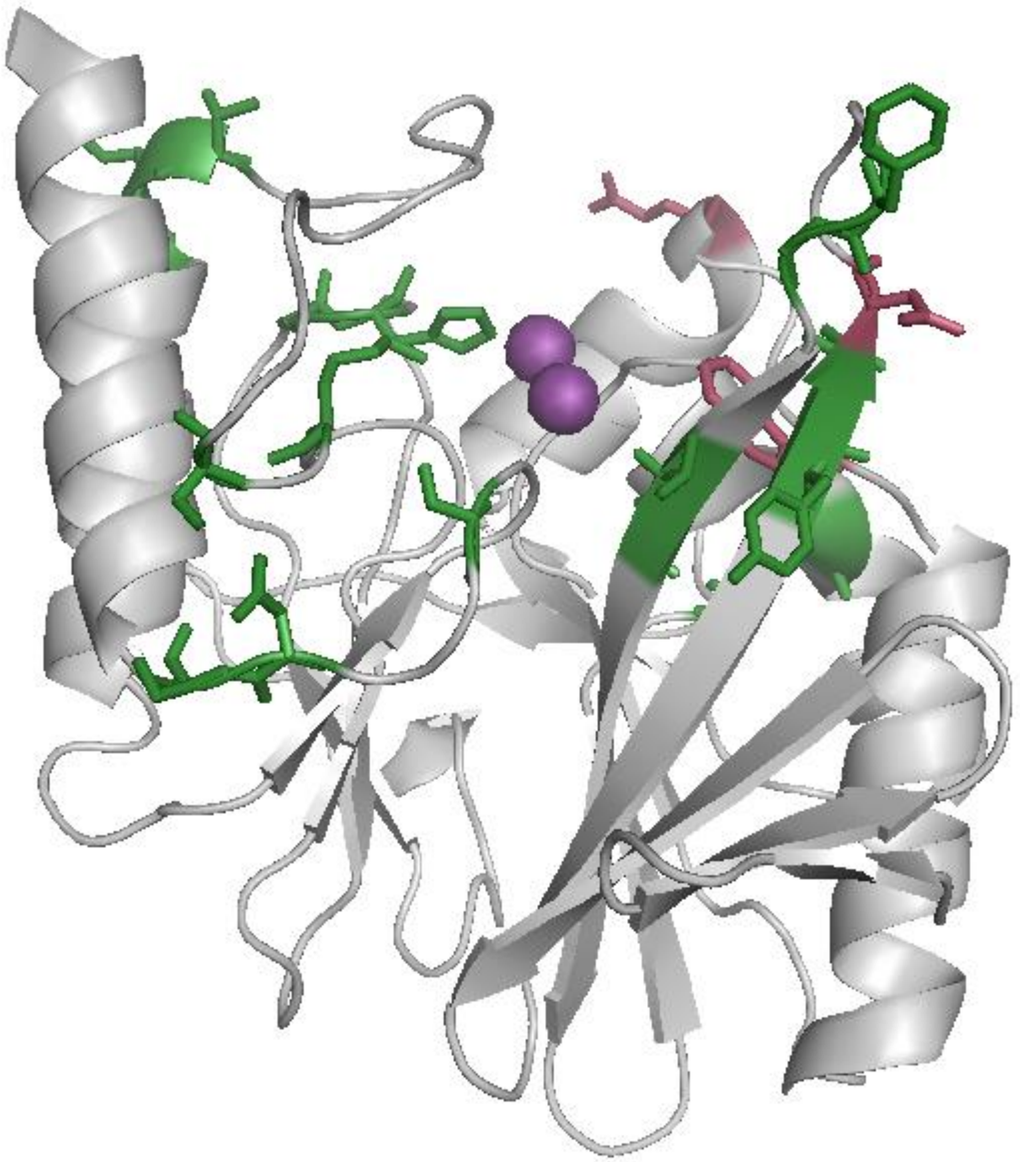

Figure S8. Highlighted green and pink amino acid residues on the NDM-1 (*PDB:4exs*) secondary structure with SSP corresponding to weighted average (^{1}H,^{15}N $\Delta\delta$) greater than the population mean plus standard deviation (μ+1σ) . Pink colored residues are highlighted due to additional NOE correlations detected in the 3D ^{15}N-filtered HSQC-NOESY.

6. **The protein sequence of NDM-1**

Figure S9. Sequence of pET27b(+)_His-NDM-1

LacI

LacI promoter

T7 promoter

Lac operator

Ribosome binding site

PelB sequence

NDM-1 gene

TEV site encoding bases

His-tag

TGGCGAATGGGACGCGCCCTGTAGCGGCGCATTAAGCGCGGCGGGTGTGGTGGTTA
CGCGCAGCGTGACCGCTACACTTGCCAGCGCCCTAGCGCCCGCTCCTTTCGCTTTCTT
CCCTTCCTTTCTCGCCACGTTCGCCGGCTTTCCCCGTCAAGCTCTAAATCGGGGGCTC
CCTTTAGGGTTCCGATTTAGTGCTTTACGGCACCTCGACCCCAAAAAACTTGATTAG
GGTGATGGTTCACGTAGTGGGCCATCGCCCTGATAGACGGTTTTTCGCCCTTTGACG
TTGGAGTCCACGTTCTTTAATAGTGGACTCTTGTTCCAAACTGGAACAACACTCAAC
CCTATCTCGGTCTATTCTTTTGATTTATAAGGGATTTTGCCGATTTCGGCCTATTGGTT
AAAAAATGAGCTGATTTAACAAAAATTTAACGCGAATTTTAACAAAATATTAACGTT
TACAATTTCAGGTGGCACTTTTCGGGGAAATGTGCGCGGAACCCCTATTTGTTTATTT
TTCTAAATACATTCAAATATGTATCCGCTCATGAATTAATTCTTAGAAAAACTCATC
GAGCATCAAATGAAACTGCAATTTATTCATATCAGGATTATCAATACCATATTTTTG
AAAAAGCCGTTTCTGTAATGAAGGAGAAAACTCACCGAGGCAGTTCCATAGGATGG
CAAGATCCTGGTATCGGTCTGCGATTCCGACTCGTCCAACATCAATACAACCTATTA
ATTTCCCCTCGTCAAAAATAAGGTTATCAAGTGAGAAATCACCATGAGTGACGACTG
AATCCGGTGAGAATGGCAAAAGTTTATGCATTTCTTTCCAGACTTGTTCAACAGGCC
AGCCATTACGCTCGTCATCAAAATCACTCGCATCAACCAAACCGTTATTCATTCGTG
ATTGCGCCTGAGCGAGACGAAATACGCGATCGCTGTTAAAAGGACAATTACAAACA
GGAATCGAATGCAACCGGCGCAGGAACACTGCCAGCGCATCAACAATATTTTCACC
TGAATCAGGATATTCTTCTAATACCTGGAATGCTGTTTTCCCGGGGATCGCAGTGGT
GAGTAACCATGCATCATCAGGAGTACGGATAAAATGCTTGATGGTCGGAAGAGGCA
TAAATTCCGTCAGCCAGTTTAGTCTGACCATCTCATCTGTAACATCATTGGCAACGCT
ACCTTTGCCATGTTTCAGAAACAACTCTGGCGCATCGGGCTTCCCATACAATCGATA
GATTGTCGCACCTGATTGCCCGACATTATCGCGAGCCCATTTATACCCATATAAATC
AGCATCCATGTTGGAATTTAATCGCGGCCTAGAGCAAGACGTTTCCCGTTGAATATG
GCTCATAACACCCCTTGTATTACTGTTTATGTAAGCAGACAGTTTTATTGTTCATGAC

CAAAATCCCTTAACGTGAGTTTTCGTTCCACTGAGCGTCAGACCCCGTAGAAAAGAT
CAAAGGATCTTCTTGAGATCCTTTTTTTCTGCGCGTAATCTGCTGCTTGCAAACAAAA
AAACCACCGCTACCAGCGGTGGTTTGTTTGCCGGATCAAGAGCTACCAACTCTTTTT
CCGAAGGTAACTGGCTTCAGCAGAGCGCAGATACCAAATACTGTCCTTCTAGTGTAG
CCGTAGTTAGGCCACCACTTCAAGAACTCTGTAGCACCGCCTACATACCTCGCTCTG
CTAATCCTGTTACCAGTGGCTGCTGCCAGTGGCGATAAGTCGTGTCTTACCGGGTTG
GACTCAAGACGATAGTTACCGGATAAGGCGCAGCGGTCGGGCTGAACGGGGGGTTC
GTGCACACAGCCCAGCTTGGAGCGAACGACCTACACCGAACTGAGATACCTACAGC
GTGAGCTATGAGAAAGCGCCACGCTTCCCGAAGGGAGAAAGGCGGACAGGTATCCG
GTAAGCGGCAGGGTCGGAACAGGAGAGCGCACGAGGGAGCTTCCAGGGGGAAACG
CCTGGTATCTTTATAGTCCTGTCGGGTTTCGCCACCTCTGACTTGAGCGTCGATTTTT
GTGATGCTCGTCAGGGGGGCGGAGCCTATGGAAAAACGCCAGCAACGCGGCCTTTT
TACGGTTCCTGGCCTTTTGCTGGCCTTTTGCTCACATGTTCTTTCCTGCGTTATCCCCT
GATTCTGTGGATAACCGTATTACCGCCTTTGAGTGAGCTGATACCGCTCGCCGCAGC
CGAACGACCGAGCGCAGCGAGTCAGTGAGCGAGGAAGCGGAAGAGCGCCTGATGC
GGTATTTTCTCCTTACGCATCTGTGCGGTATTTCACACCGCATATATGGTGCACTCTC
AGTACAATCTGCTCTGATGCCGCATAGTTAAGCCAGTATACACTCCGCTATCGCTAC
GTGACTGGGTCATGGCTGCGCCCCGACACCCGCCAACACCCGCTGACGCGCCCTGA
CGGGCTTGTCTGCTCCCGGCATCCGCTTACAGACAAGCTGTGACCGTCTCCGGGAGC
TGCATGTGTCAGAGGTTTTCACCGTCATCACCGAAACGCGCGAGGCAGCTGCGGTAA
AGCTCATCAGCGTGGTCGTGAAGCGATTCACAGATGTCTGCCTGTTCATCCGCGTCC
AGCTCGTTGAGTTTCTCCAGAAGCGTTAATGTCTGGCTTCTGATAAAGCGGGCCATG
TTAAGGGCGGTTTTTTCCTGTTTGGTCACTGATGCCTCCGTGTAAGGGGGATTTCTGT
TCATGGGGGTAATGATACCGATGAAACGAGAGAGGATGCTCACGATACGGGTTACT
GATGATGAACATGCCCGGTTACTGGAACGTTGTGAGGGTAAACAACTGGCGGTATG
GATGCGGCGGGACCAGAGAAAAATCACTCAGGGTCAATGCCAGCGCTTCGTTAATA
CAGATGTAGGTGTTCCACAGGGTAGCCAGCAGCATCCTGCGATGCAGATCCGGAAC
ATAATGGTGCAGGGCGCTGACTTCCGCGTTTCCAGACTTTACGAAACACGGAAACCG
AAGACCATTCATGTTGTTGCTCAGGTCGCAGACGTTTTGCAGCAGCAGTCGCTTCAC
GTTCGCTCGCGTATCGGTGATTCATTCTGCTAACCAGTAAGGCAACCCCGCCAGCCT
AGCCGGGTCCTCAACGACAGGAGCACGATCATGCGCACCCGTGGGGCCGCCATGCC
GGCGATAATGGCCTGCTTCTCGCCGAAACGTTTGGTGGCGGGACCAGTGACGAAGG
CTTGAGCGAGGGCGTGCAAGATTCCGAATACCGCAAGCGACAGGCCGATCATCGTC
GCGCTCCAGCGAAAGCGGTCCTCGCCGAAAATGACCCAGAGCGCTGCCGGCACCTG
TCCTACGAGTTGCATGATAAAGAAGACAGTCATAAGTGCGGCGACGATAGTCATGC
CCCGCGCCCACCGGAAGGAGCTGACTGGGTTGAAGGCTCTCAAGGGCATCGGTCGA
GATCCCGGTGCCTAATGAGTGAGCTAACTTACATTAATTGCGTTGCGCTCACTGCCC
GCTTTCCAGTCGGGAAACCTGTCGTGCCAGCTGCATTAATGAATCGGCCAACGCGCG
GGGAGAGGCGGTTTGCGTATTGGGCGCCAGGGTGGTTTTTCTTTTCACCAGTGAGAC
GGGCAACAGCTGATTGCCCTTCACCGCCTGGCCCTGAGAGAGTTGCAGCAAGCGGT
CCACGCTGGTTTGCCCCAGCAGGCGAAAATCCTGTTTGATGGTGGTTAACGGCGGGA
TATAACATGAGCTGTCTTCGGTATCGTCGTATCCCACTACCGAGATATCCGCACCAA
CGCGCAGCCCGGACTCGGTAATGGCGCGCATTGCGCCCAGCGCCATCTGATCGTTGG
CAACCAGCATCGCAGTGGGAACGATGCCCTCATTCAGCATTTGCATGGTTTGTTGAA
AACCGGACATGGCACTCCAGTCGCCTTCCCGTTCCGCTATCGGCTGAATTTGATTGC
GAGTGAGATATTTATGCCAGCCAGCCAGACGCAGACGCGCCGAGACAGAACTTAAT

GGGCCCGCTAACAGCGCGATTTGCTGGTGACCCAATGCGACCAGATGCTCCACGCC
CAGTCGCGTACCGTCTTCATGGGAGAAAATAATACTGTTGATGGGTGTCTGGTCAGA
GACATCAAGAAATAACGCCGGAACATTAGTGCAGGCAGCTTCCACAGCAATGGCAT
CCTGGTCATCCAGCGGATAGTTAATGATCAGCCCACTGACGCGTTGCGCGAGAAGAT
TGTGCACCGCCGCTTTACAGGCTTCGACGCCGCTTCGTTCTACCATCGACACCACCA
CGCTGGCACCCAGTTGATCGGCGCGAGATTTAATCGCCGCGACAATTTGCGACGGC
GCGTGCAGGGCCAGACTGGAGGTGGCAACGCCAATCAGCAACGACTGTTTGCCCGC
CAGTTGTTGTGCCACGCGGTTGGGAATGTAATTCAGCTCCGCCATCGCCGCTTCCAC
TTTTTCCCGCGTTTTCGCAGAAACGTGGCTGGCCTGGTTCACCACGCGGGAAACGGT
CTGATAAGAGACACCGGCATACTCTGCGACATCGTATAACGTTACTGGTTTCACATT
CACCACCCTGAATTGACTCTCTTCCGGGCGCTATCATGCCATACCGCGAAAGGTTTT
GCGCCATTCGATGGTGTCCGGGATCTCGACGCTCTCCCTTATGCGACTCCTGCATTA
GGAAGCAGCCCAGTAGTAGGTTGAGGCCGTTGAGCACCGCCGCCGCAAGGAATGGT
GCATGCAAGGAGATGGCGCCCAACAGTCCCCCGGCCACGGGGCCTGCCACCATACC
CACGCCGAAACAAGCGCTCATGAGCCCGAAGTGGCGAGCCCGATCTTCCCCATCGG
TGATGTCGGCGATATAGGCGCCAGCAACCGCACCTGTGGCGCCGGTGATGCCGGCC
ACGATGCGTCCGGCGTAGAGGATCGAGATCTCGATCCCGCGAAATTAATACGACTC
ACTATAGGGGAATTGTGAGCGGATAACAATTCCCCTCTAGAAATAATTTTGTTTAAC
TTTAAGAAGGAGATATACATATGAAATACCTGCTGCCGACCGCTGCTGCTGGTCTGC
TGCTCCTCGCTGCCCAGCCGGCGATGGCCATGgctgcacaccatcatcatcatcattcttctggtgtagatctgg
gtaccgagaacctgtacttccaaggCCAGCAAATGGAAACTGGCGACCAACGGTTTGGCGATCTGG
TTTTCCGCCAGCTCGCACCGAATGTCTGGCAGCACACTTCCTATCTCGACATGCCGG
GTTTCGGGGCAGTCGCTTCCAACGGTTTGATCGTCAGGGATGGCGGCCGCGTGCTGG
TGGTCGATACCGCCTGGACCGATGACCAGACCGCCCAGATCCTCAACTGGATCAAG
CAGGAGATCAACCTGCCGGTCGCGCTGGCGGTGGTGACTCACGCGCATCAGGACAA
GATGGGCGGTATGGACGCGCTGCATGCGGCGGGGATTGCGACTTATGCCAATGCGT
TGTCGAACCAGCTTGCCCCGCAAGAGGGGATGGTTGCGGCGCAACACAGCCTGACT
TTCGCCGCCAATGGCTGGGTCGAACCAGCAACCGCGCCCAACTTTGGCCCGCTCAAG
GTATTTTACCCCGGCCCCGGCCACACCAGTGACAATATCACCGTTGGGATCGACGGC
ACCGACATCGCTTTTGGTGGCTGCCTGATCAAGGACAGCAAGGCCAAGTCGCTCGG
CAATCTCGGTGATGCCGACACTGAGCACTACGCCGCGTCAGCGCGCGCGTTTGGTGC
GGCGTTCCCCAAGGCCAGCATGATCGTGATGAGCCATTCCGCCCCCGATAGCCGCGC
CGCAATCACTCATACGGCCCGCATGGCCGACAAGCTGCGCTAATGAGGATCCGAAT
TCGAGCTCCGTCGACAAGCTTGCGGCCGCACTCGAGATCAAACGGGCTAGCCAGCC
AGAACTCGCCCCGGAAGACCCCGAGGATGTCGAGCACCACCACCACCACCACTGAG
ATCCGGCTGCTAACAAAGCCCGAAAGGAAGCTGAGTTGGCTGCTGCCACCGCTGAG
CAATAACTAGCATAACCCCTTGGGGCCTCTAAACGGGTCTTGAGGGGTTTTTTGCTG
AAAGGAGGAACTATATCCGGAT

Figure S10. Protein sequence of the produced NDM-1 after TEV cleavage:

GQQMETGDQRFGDLVFRQLAPNVWQHTSYLDMPGFGAVASNGLIVRDGGRVLVVDTA
WTDDQTAQILNWIKQEINLPVALAVVTHAHQDKMGGMDALHAAGIATYANALSNQLA
PQEGMVAAQHSLTFAANGWVEPATAPNFGPLKVFYPGPGHTSDNITVGIDGTDIAFGGC
LIKDSKAKSLGNLGDADTEHYAASARAFGAAFPKASMIVMSHSAPDSRAAITHTARMA
DKLR

7. NMR spectra of the synthesized compounds

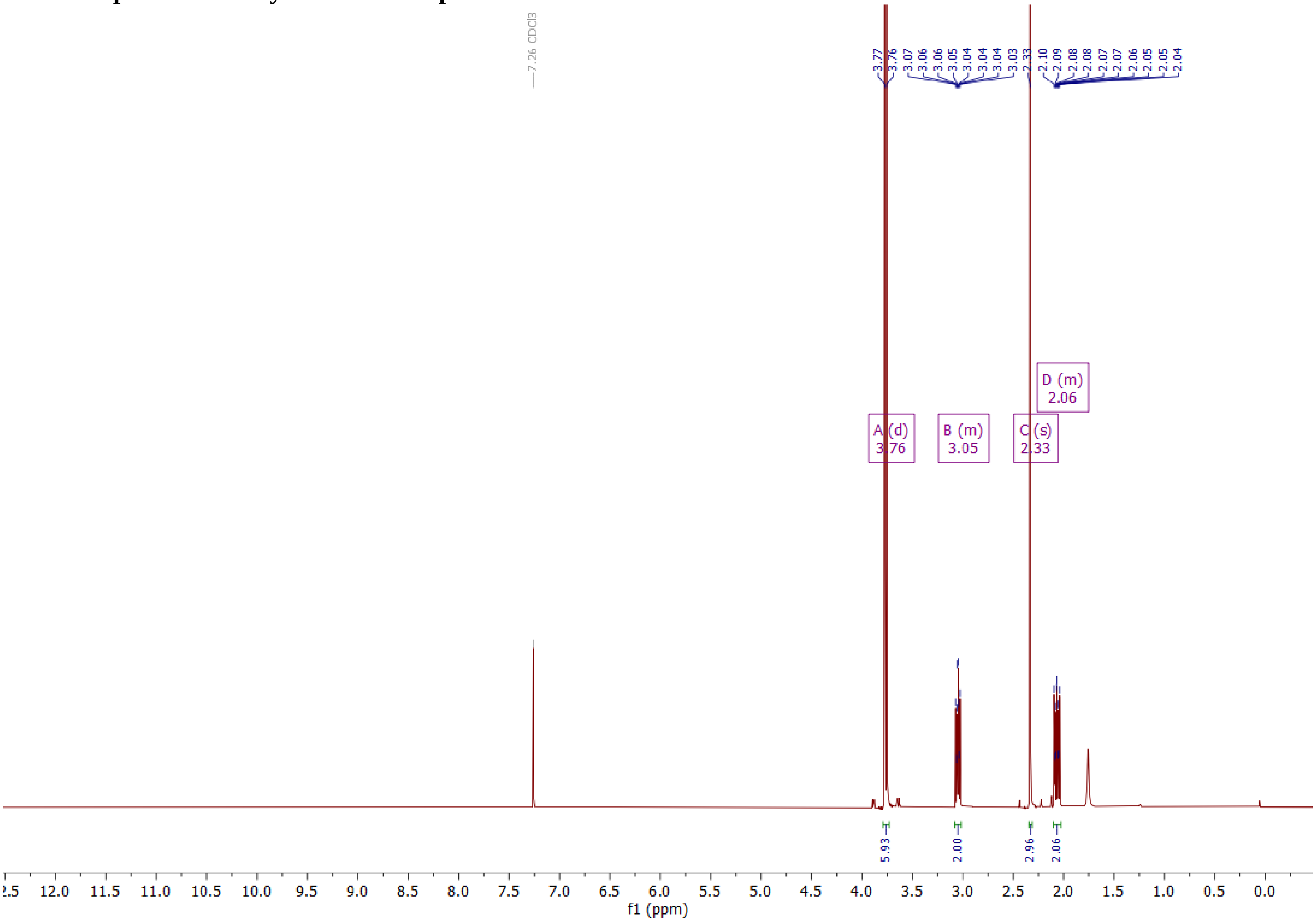

Figure S11. ^{1}H NMR spectrum of **4**.

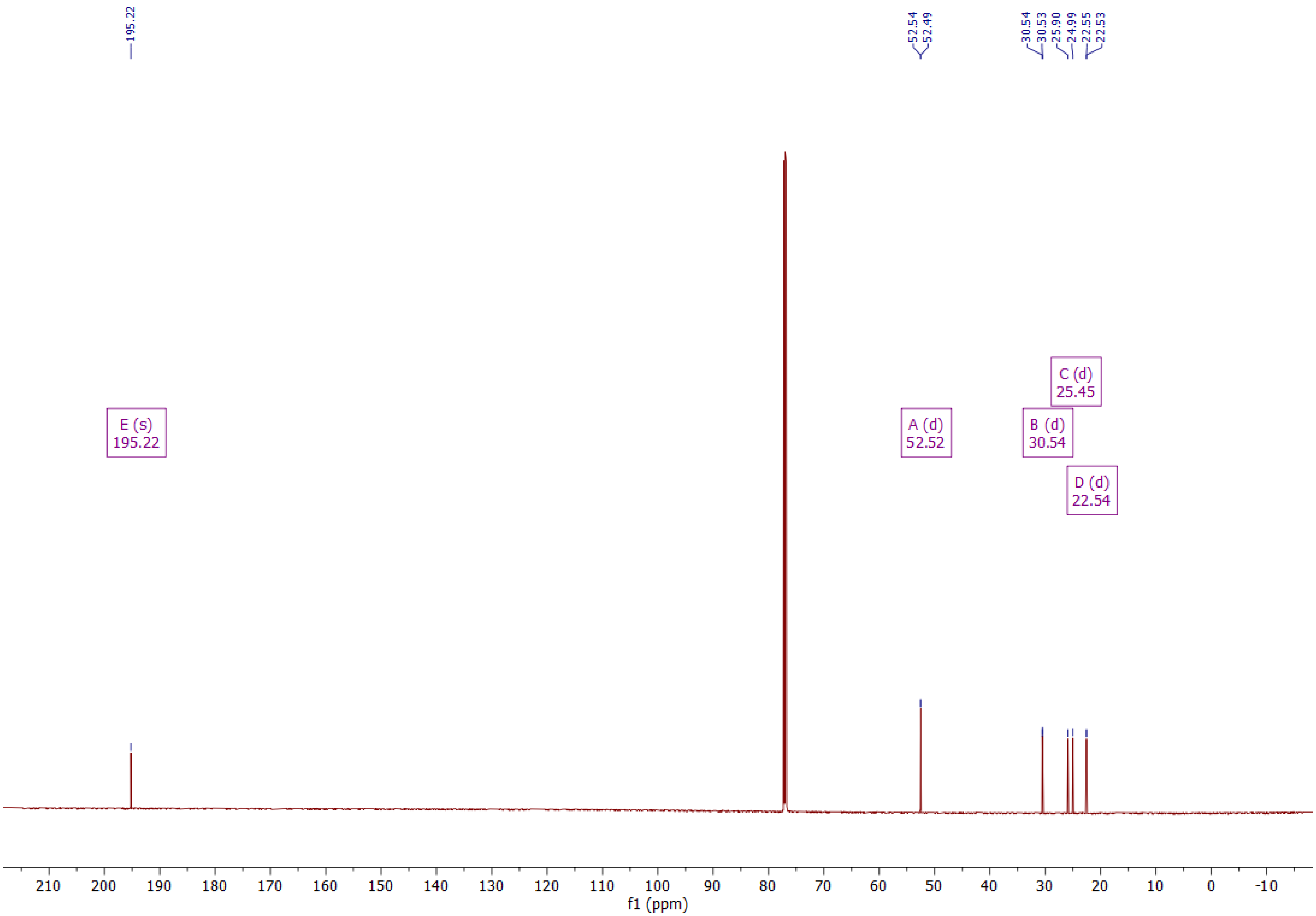

Figure S12. ^{13}C NMR spectrum of **4**.

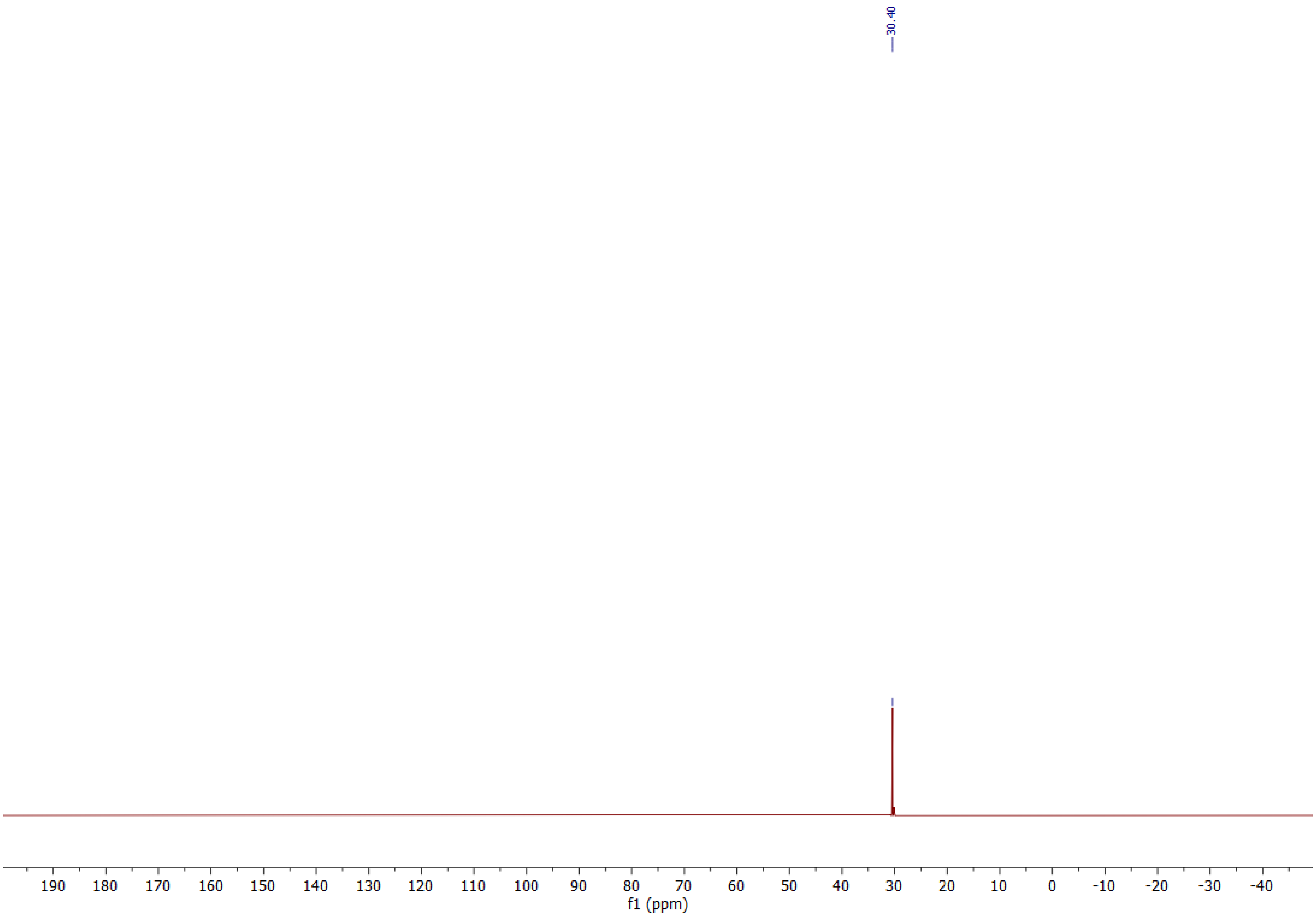

Figure S13. ^{31}P NMR spectrum of **4**.

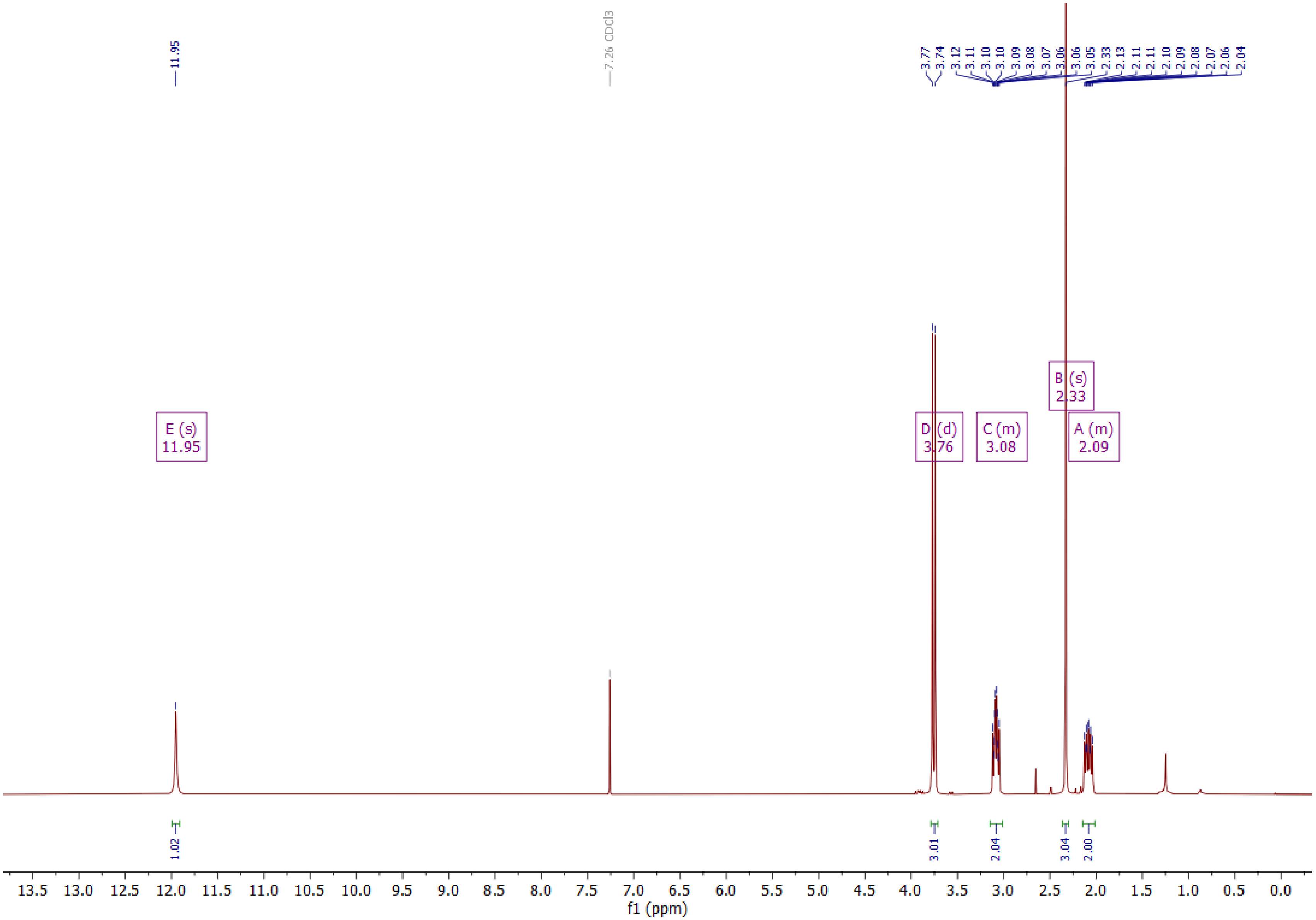

Figure S14. ^{1}H NMR spectrum of **5**.

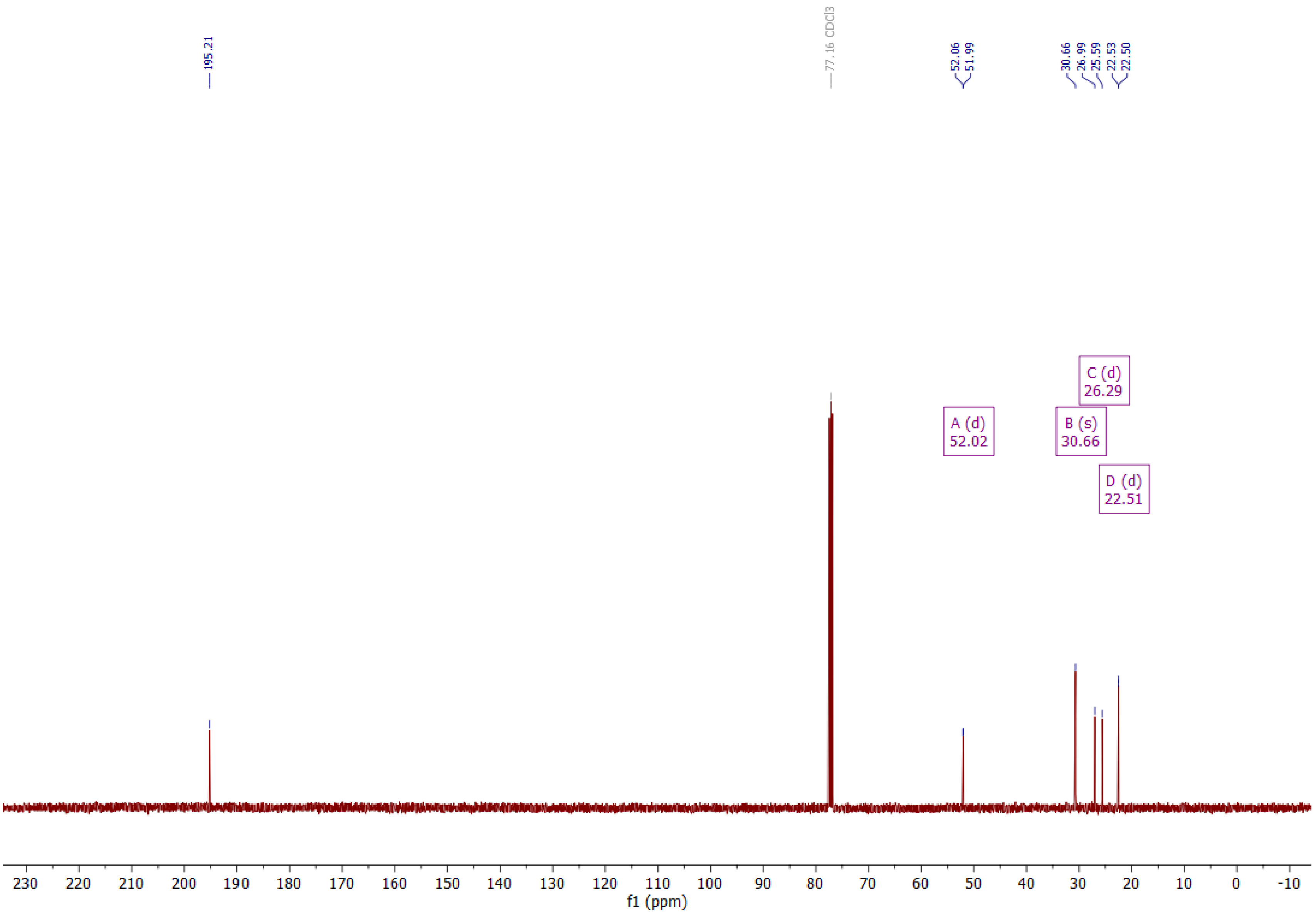

Figure S15. ^{13}C NMR spectrum of **5**.

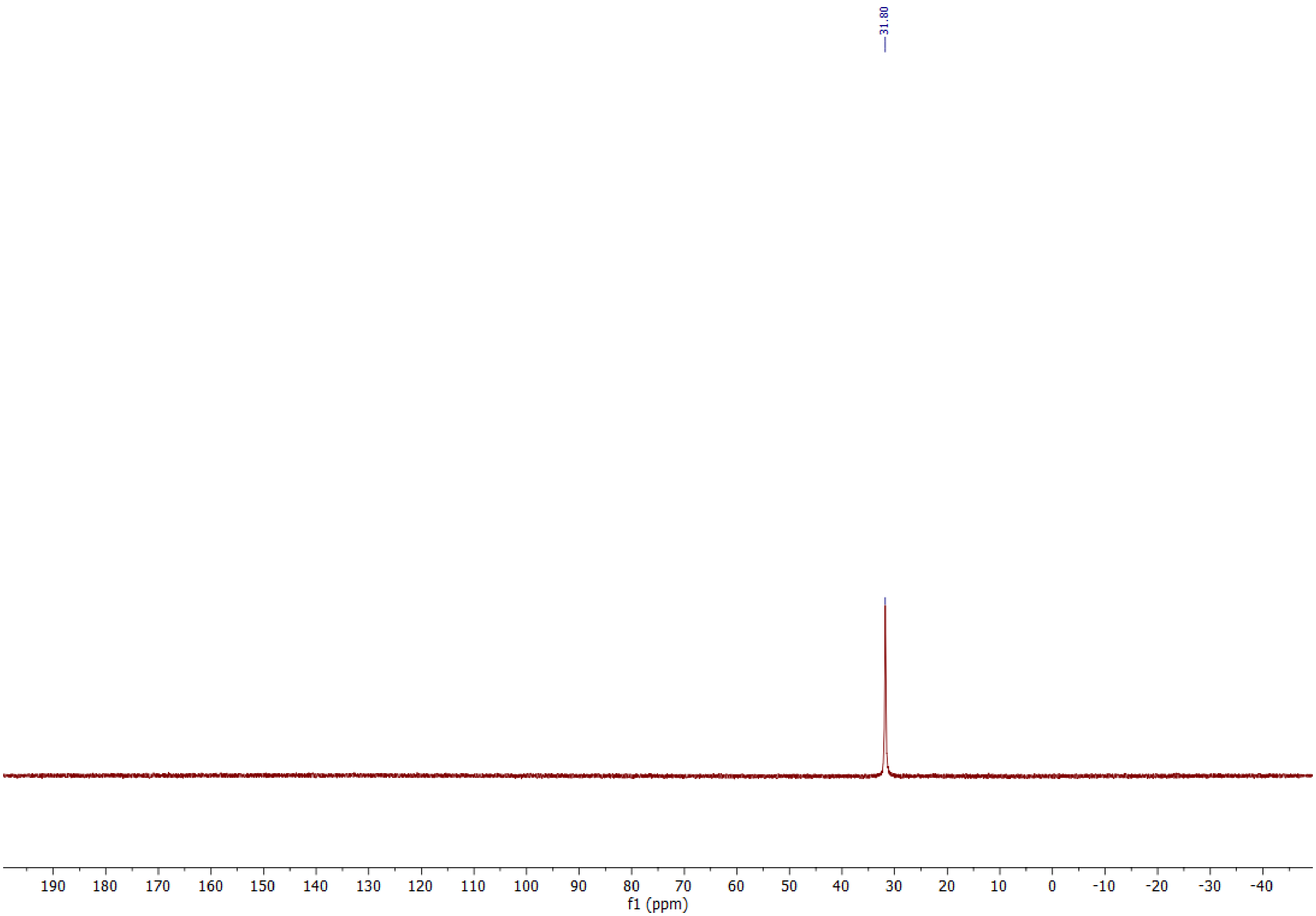

Figure S16. ^{31}P NMR spectrum of **5**.

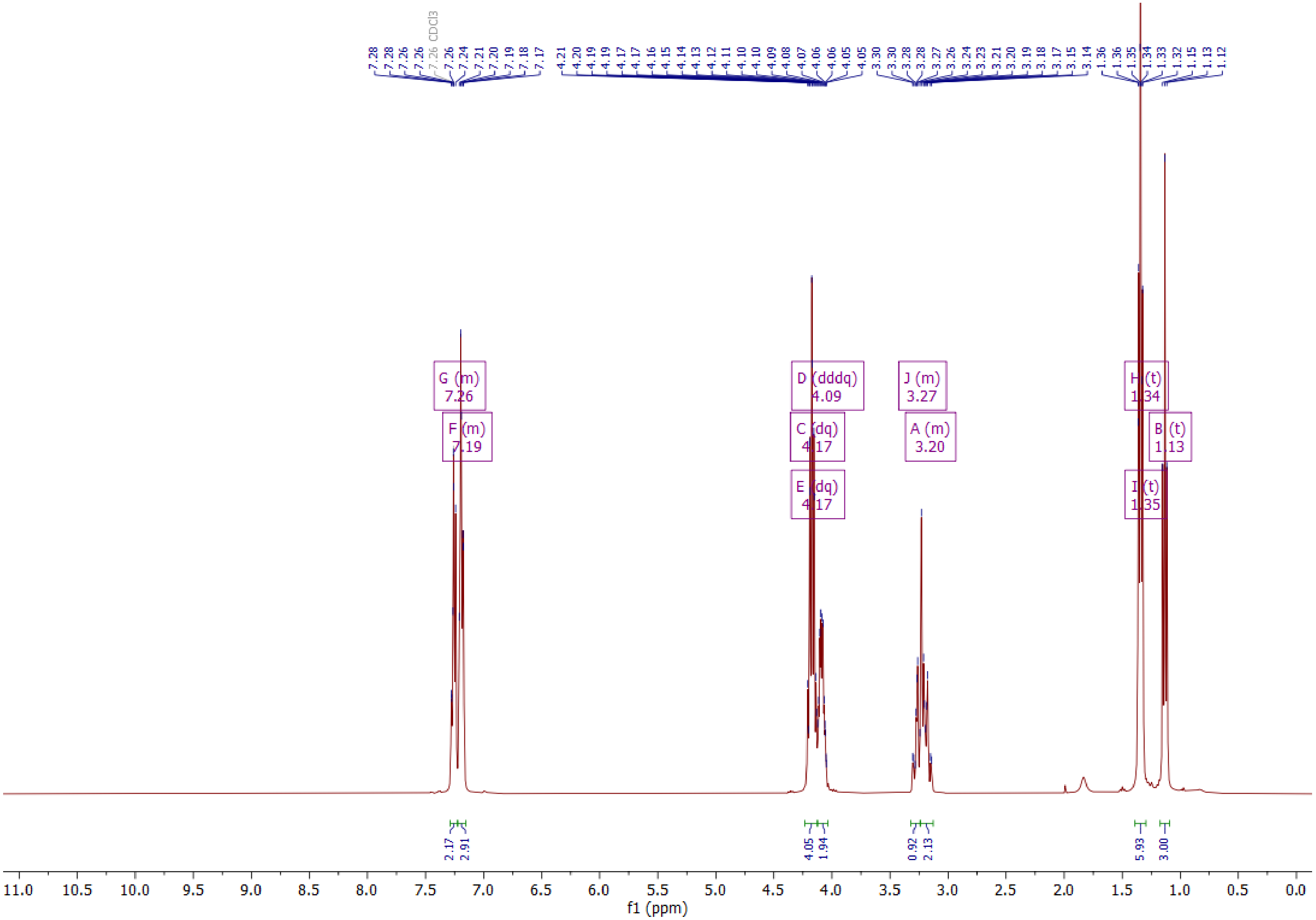

Figure S17. ^{1}H NMR spectrum of **7**.

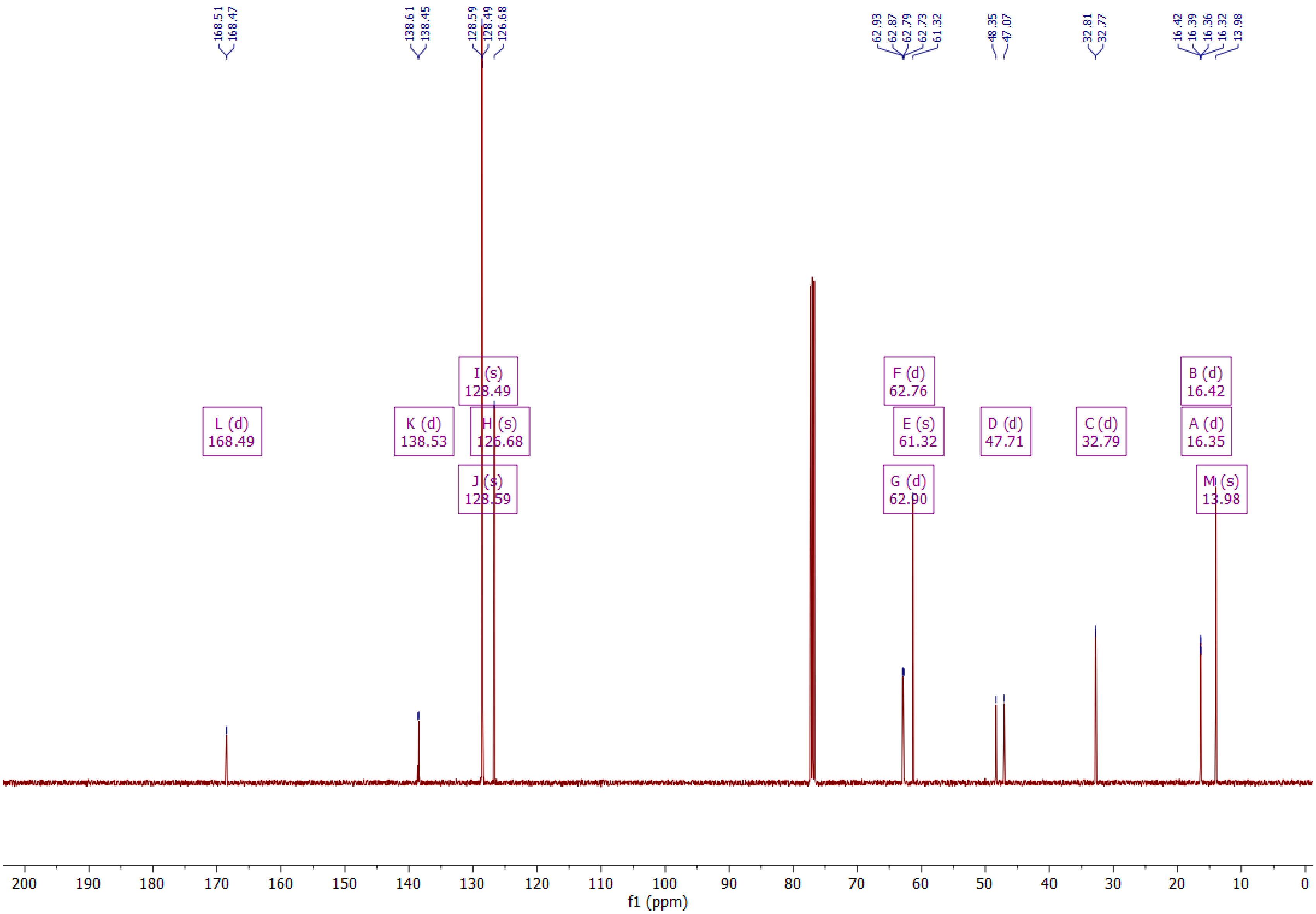

Figure S18. ^{13}C NMR spectrum of **7**.

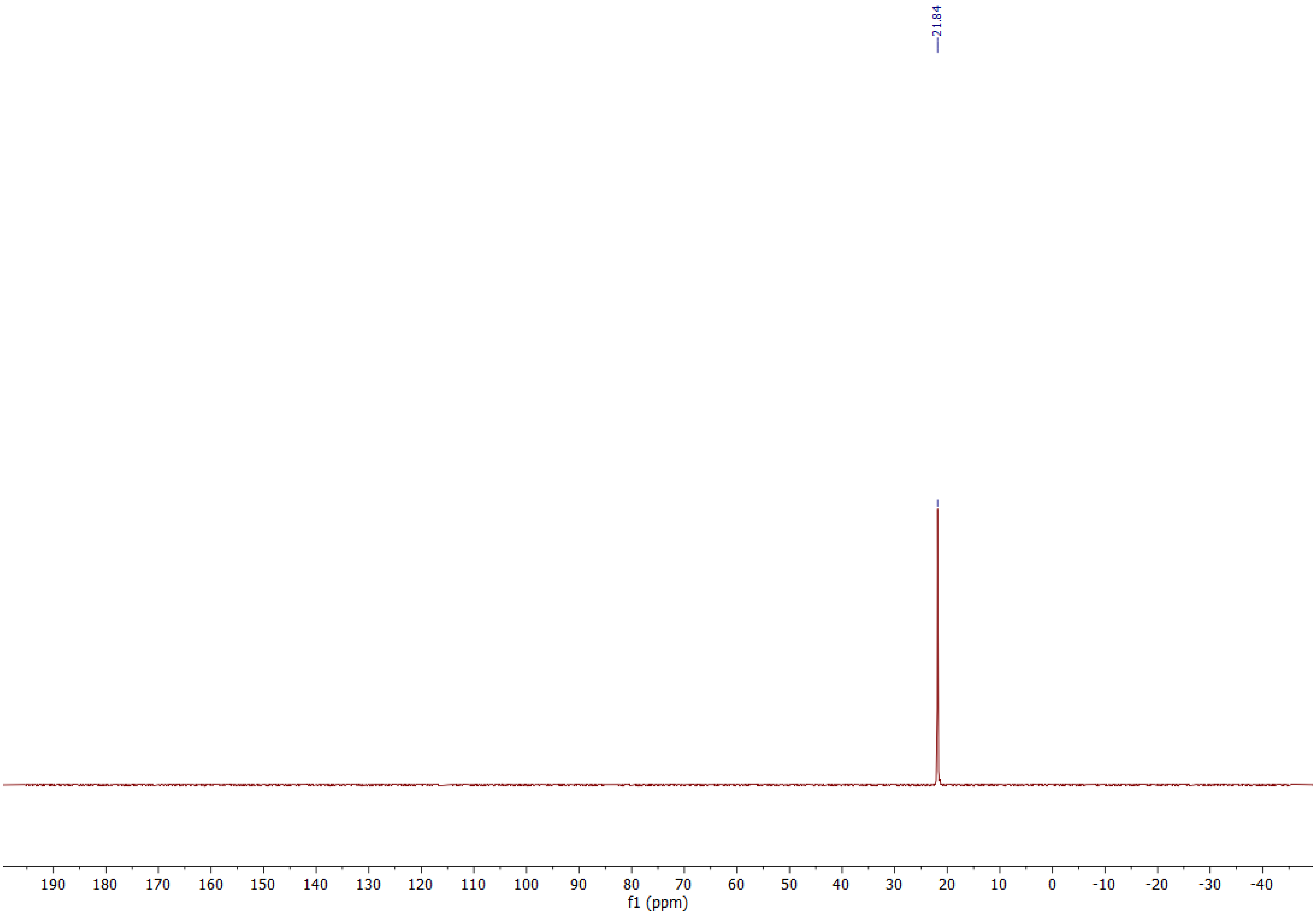

Figure S19. ^{31}P NMR spectrum of **7**.

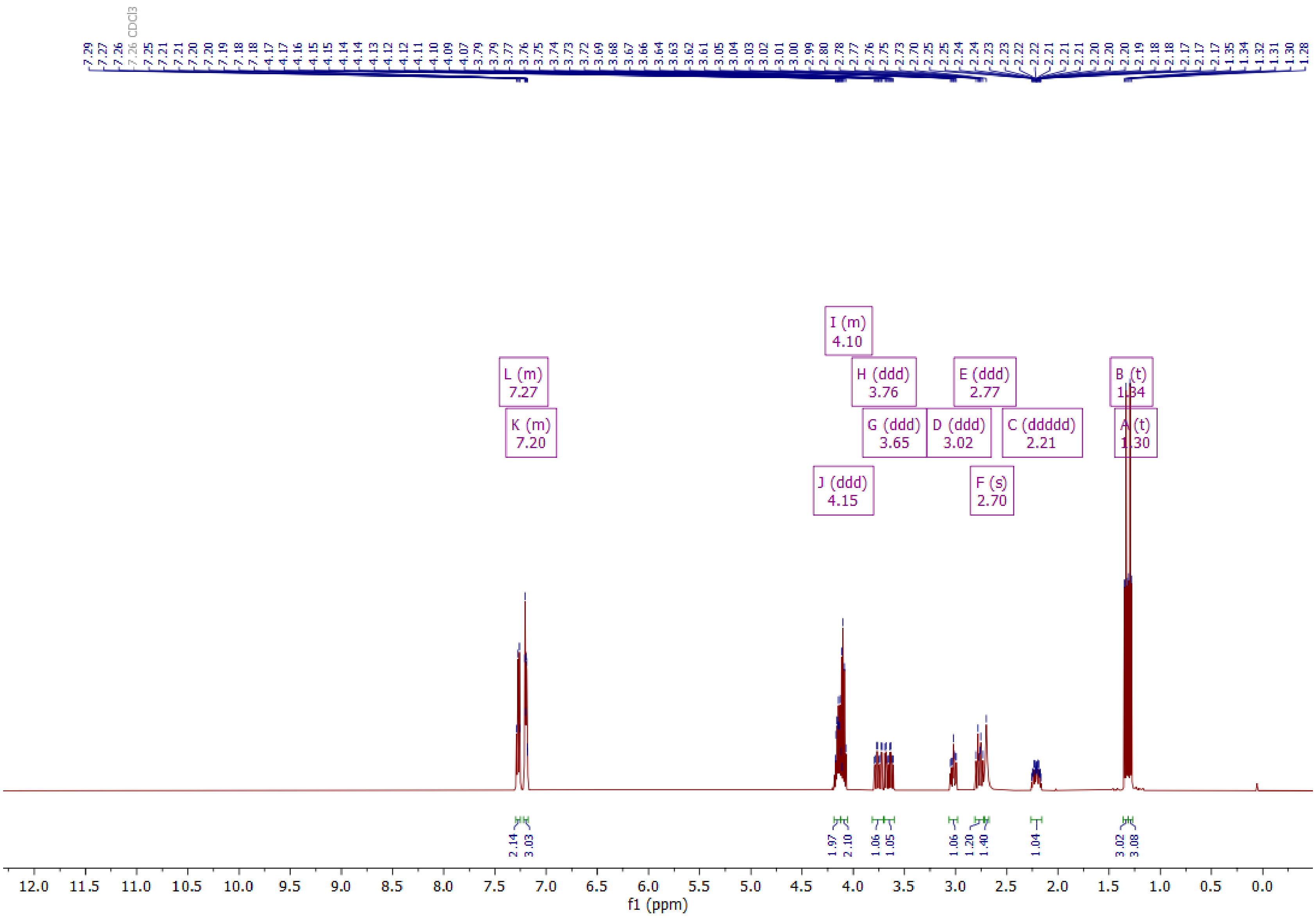

Figure S20. ^{1}H NMR spectrum of **8**.

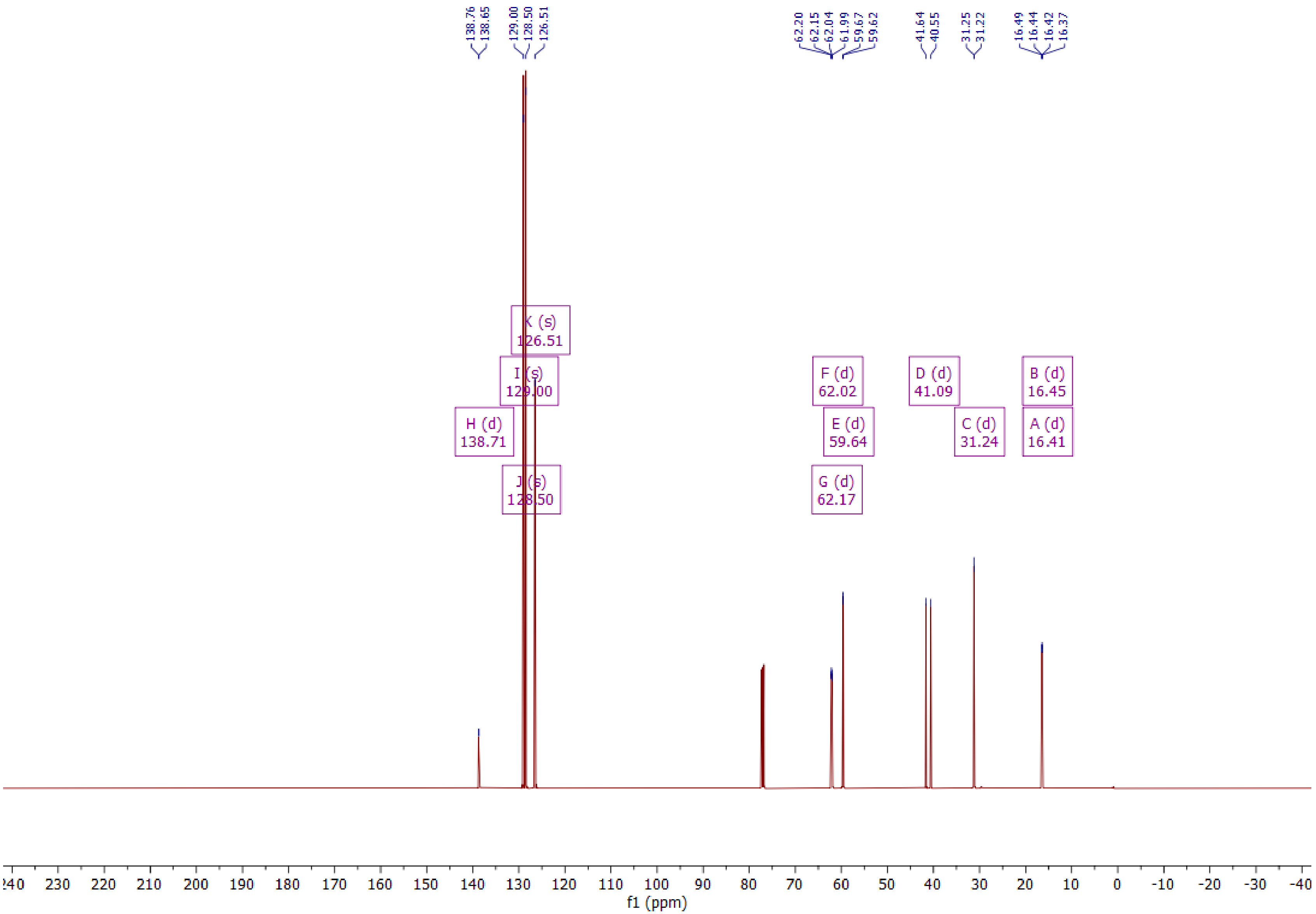

Figure S21. ^{13}C NMR spectrum of **8**.

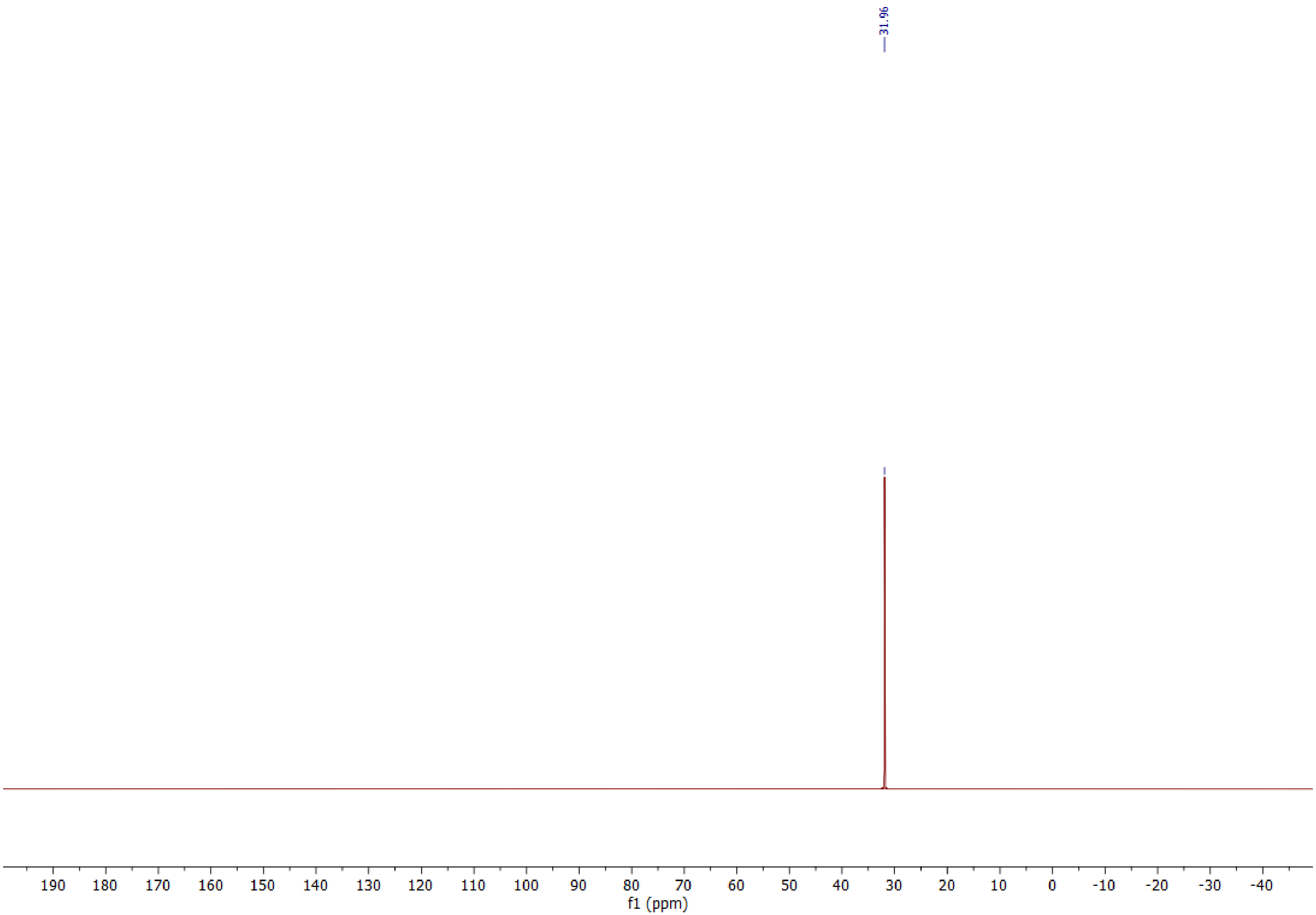

Figure S22. ^{31}P NMR spectrum of **8**.

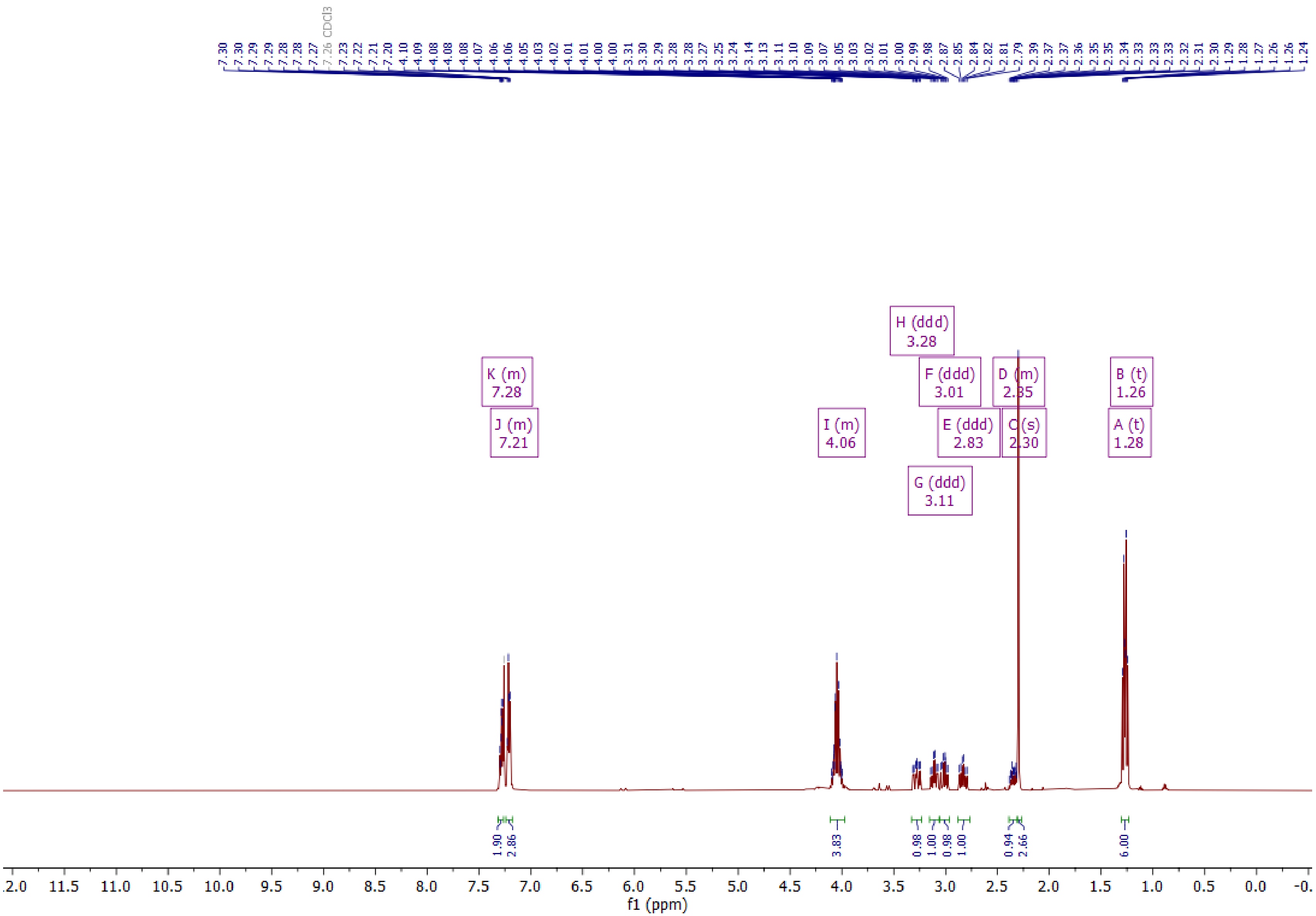

Figure S23. ^{1}H NMR spectrum of **9**.

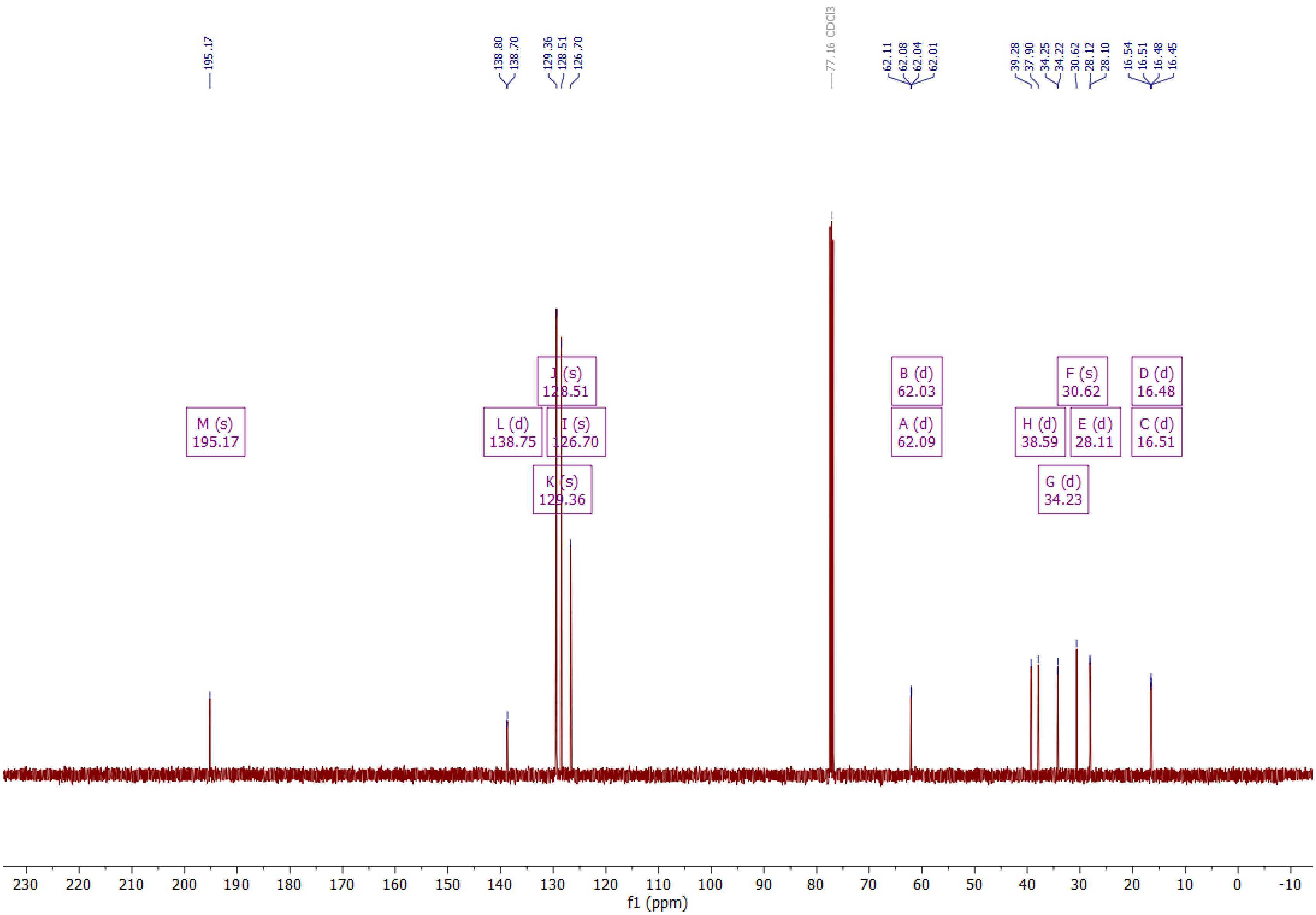

Figure S24. ^{13}C NMR spectrum of **9**.

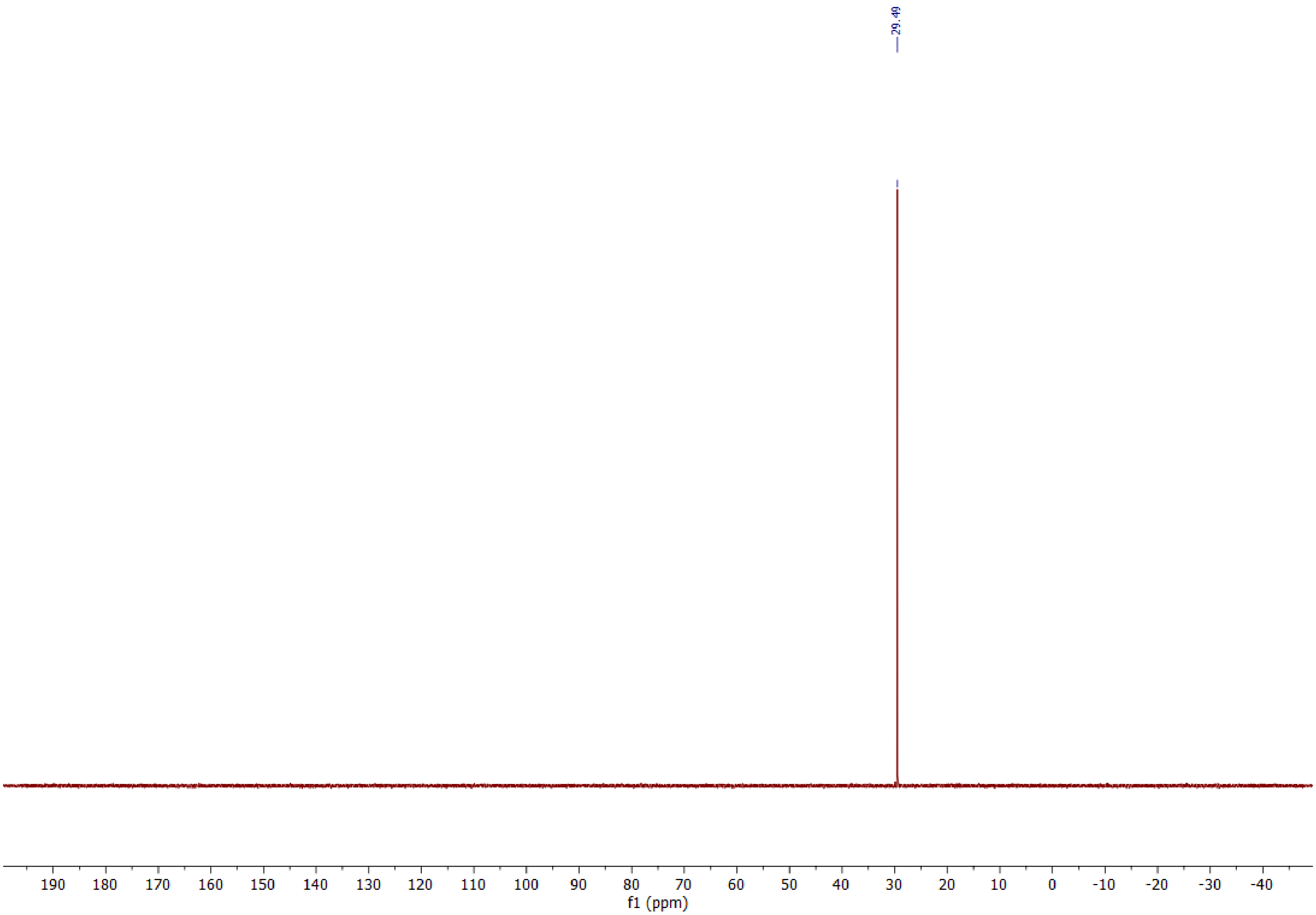

Figure S25. ^{31}P NMR spectrum of **9**.

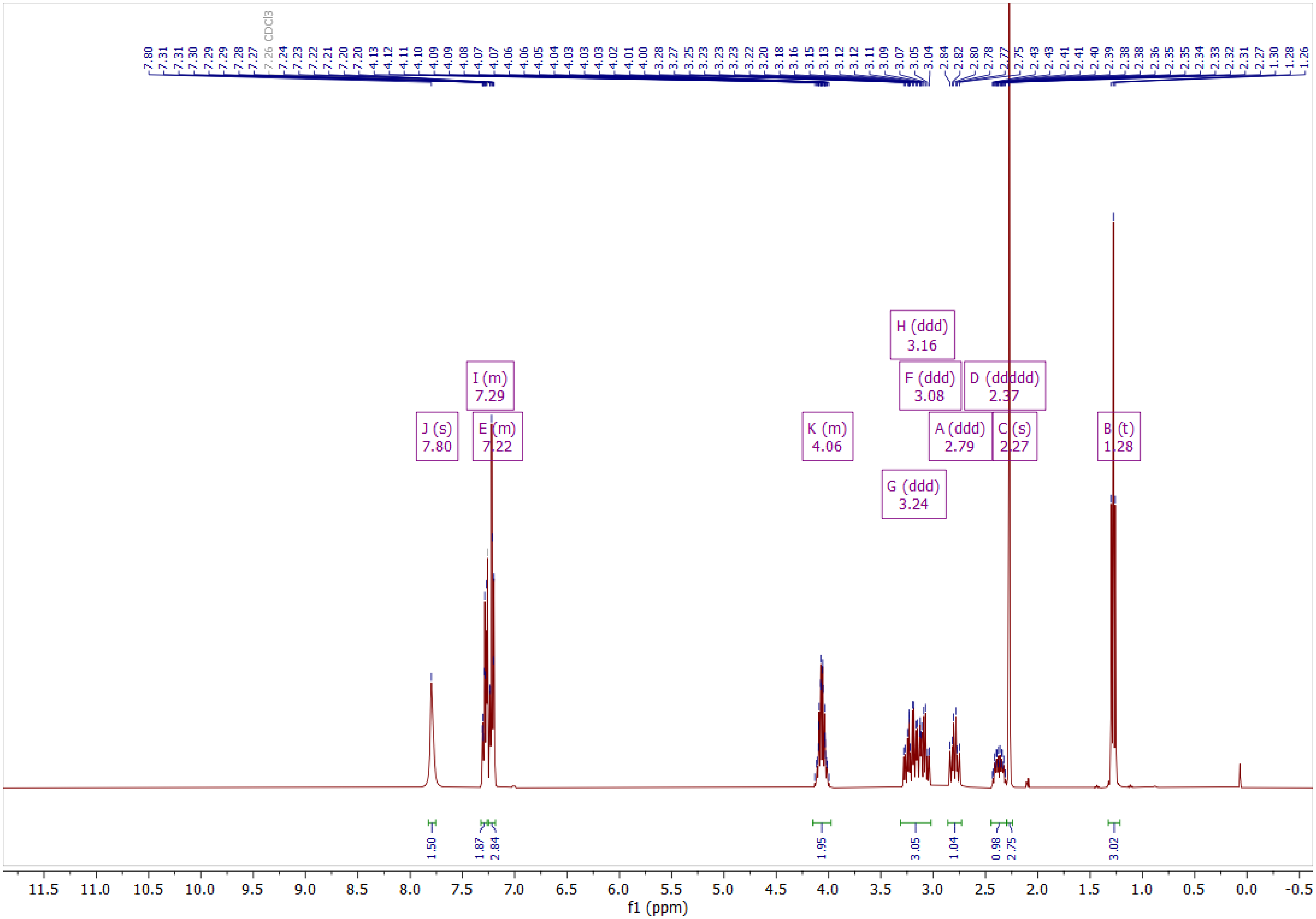

Figure S26. ^{1}H NMR spectrum of **10**.

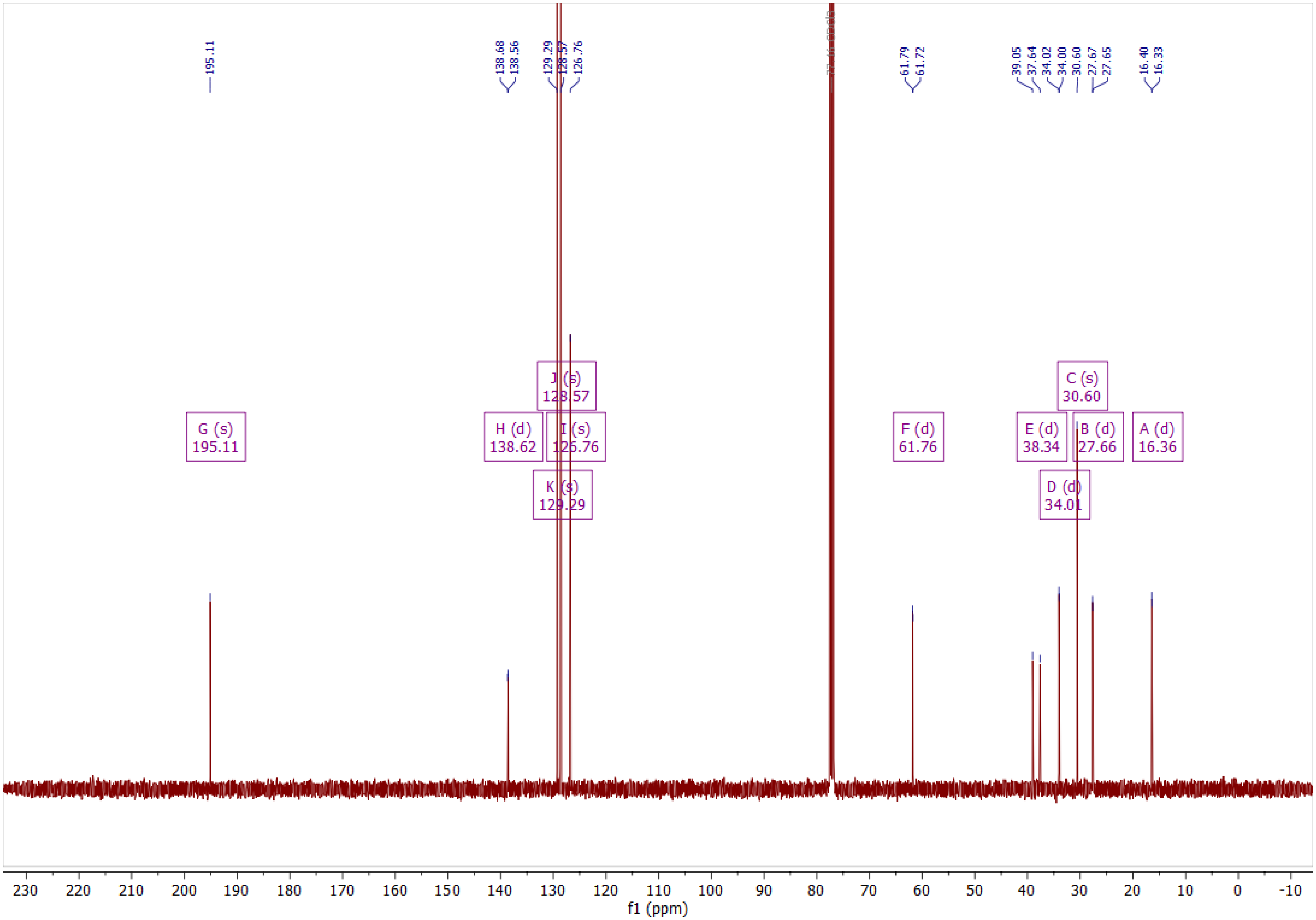

Figure S27. ^{13}C NMR spectrum of **10**.

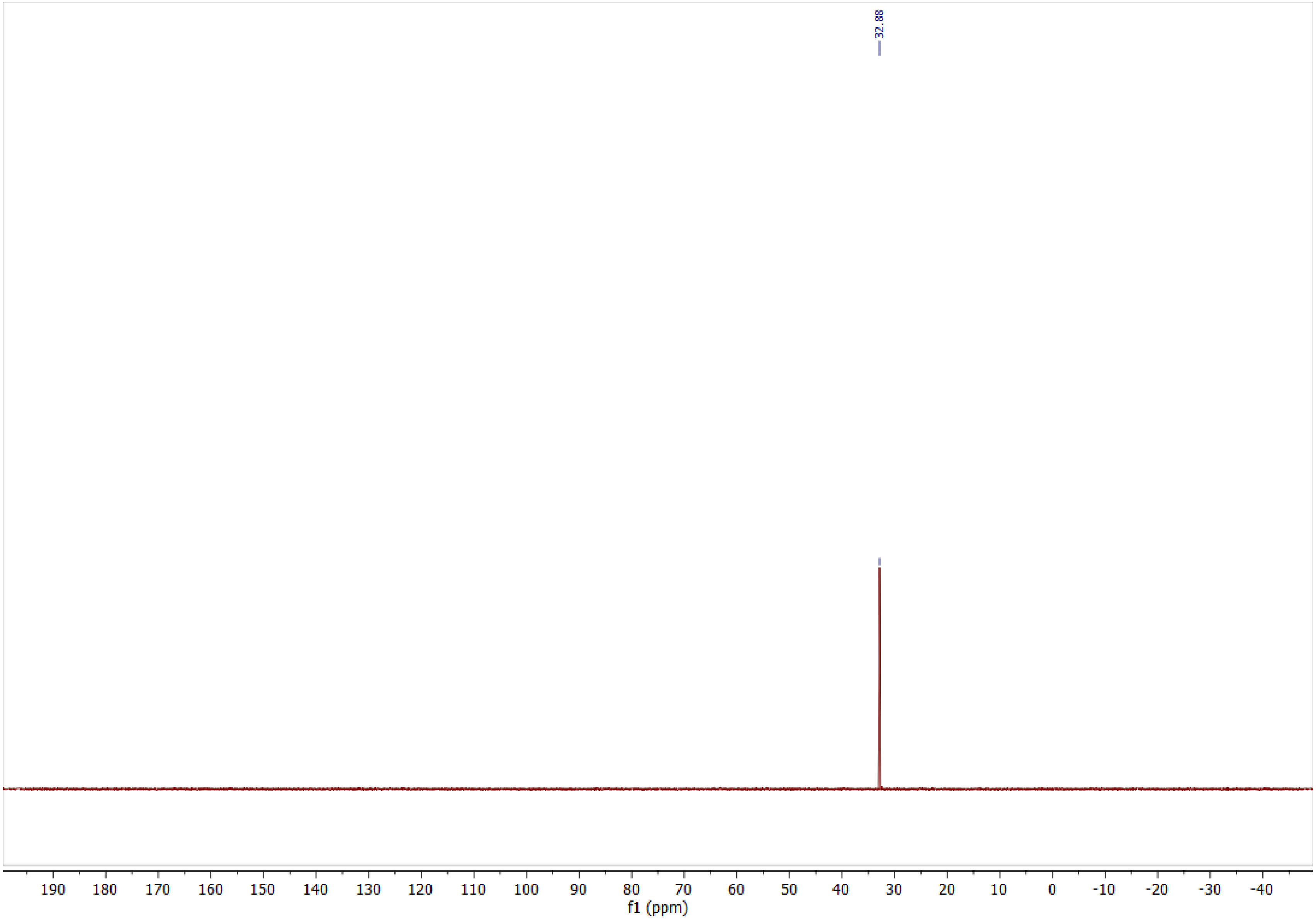

Figure S28. ^{31}P NMR spectrum of **10**.

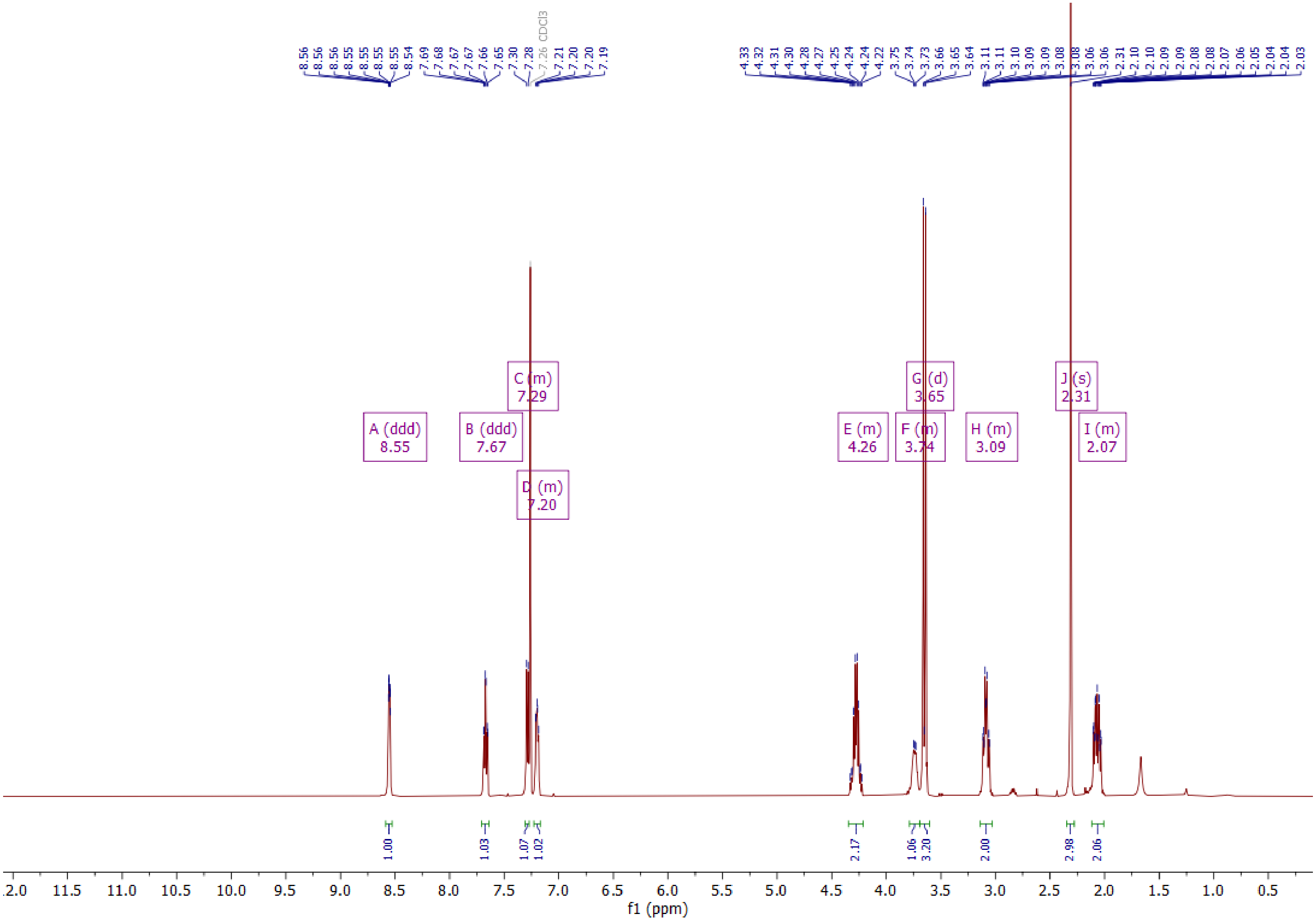

Figure S29. ^{1}H NMR spectrum of **1a**.

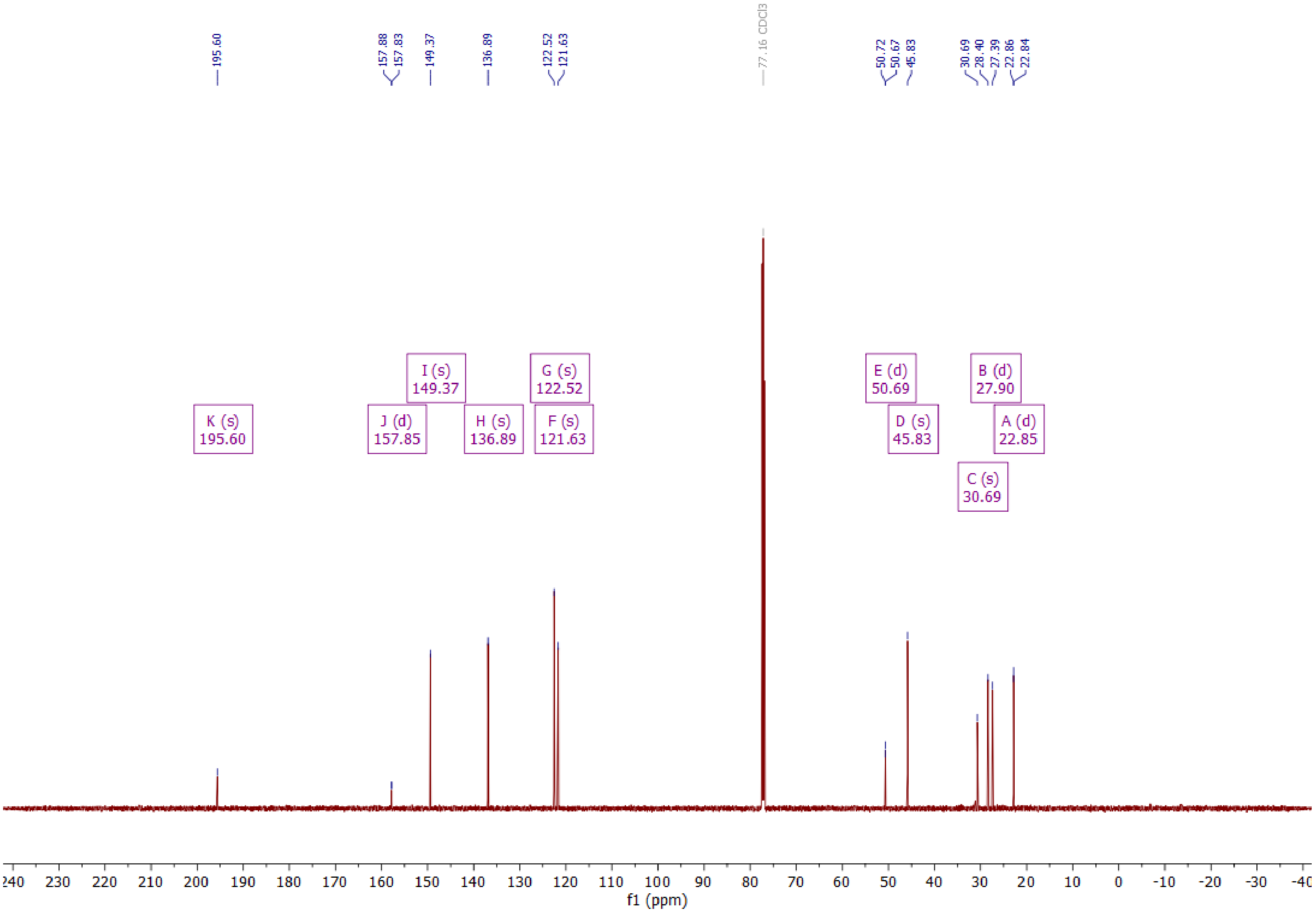

Figure S30. ^{13}C NMR spectrum of **1a**.

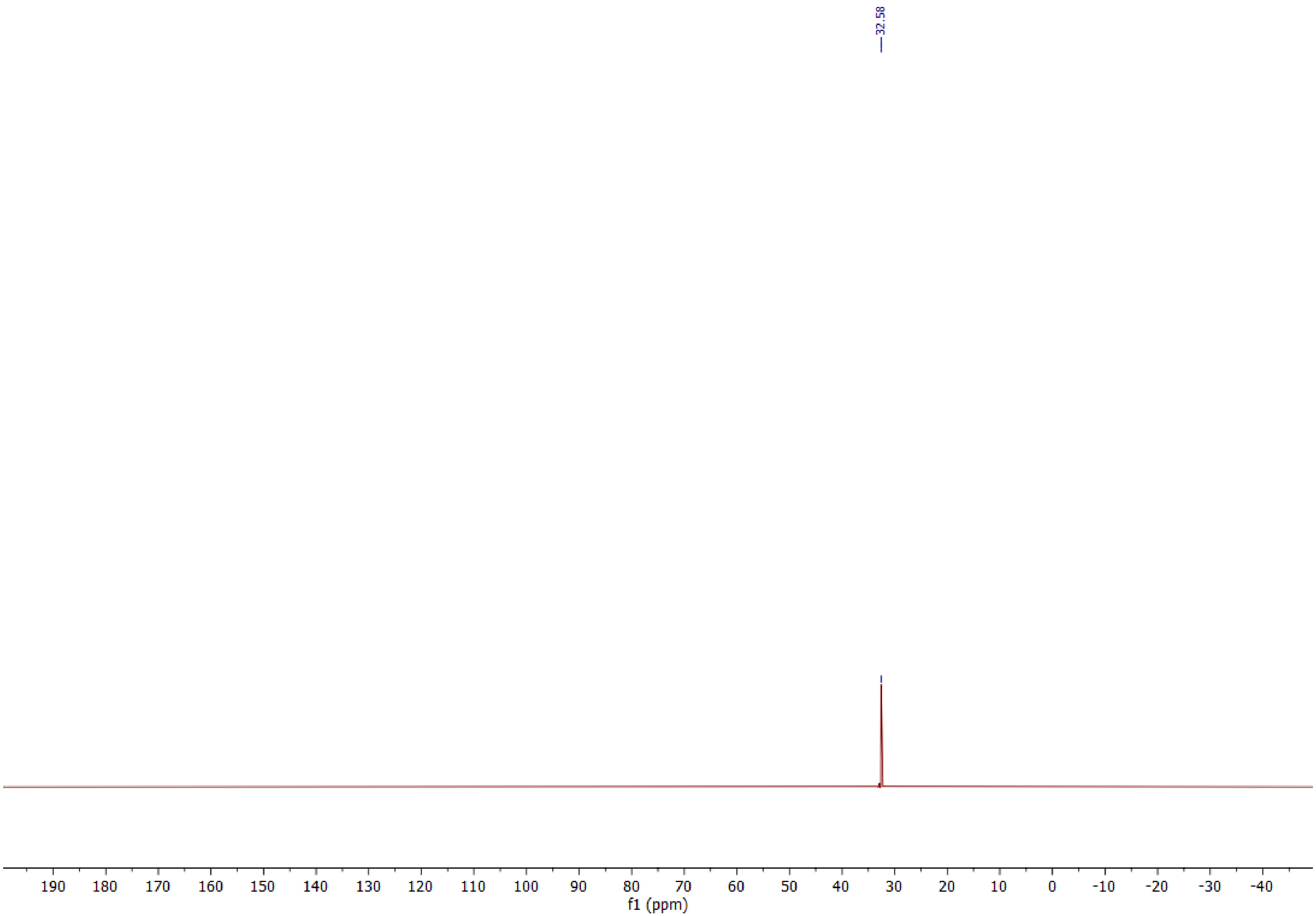

Figure S31. ^{31}P NMR spectrum of **1a**.

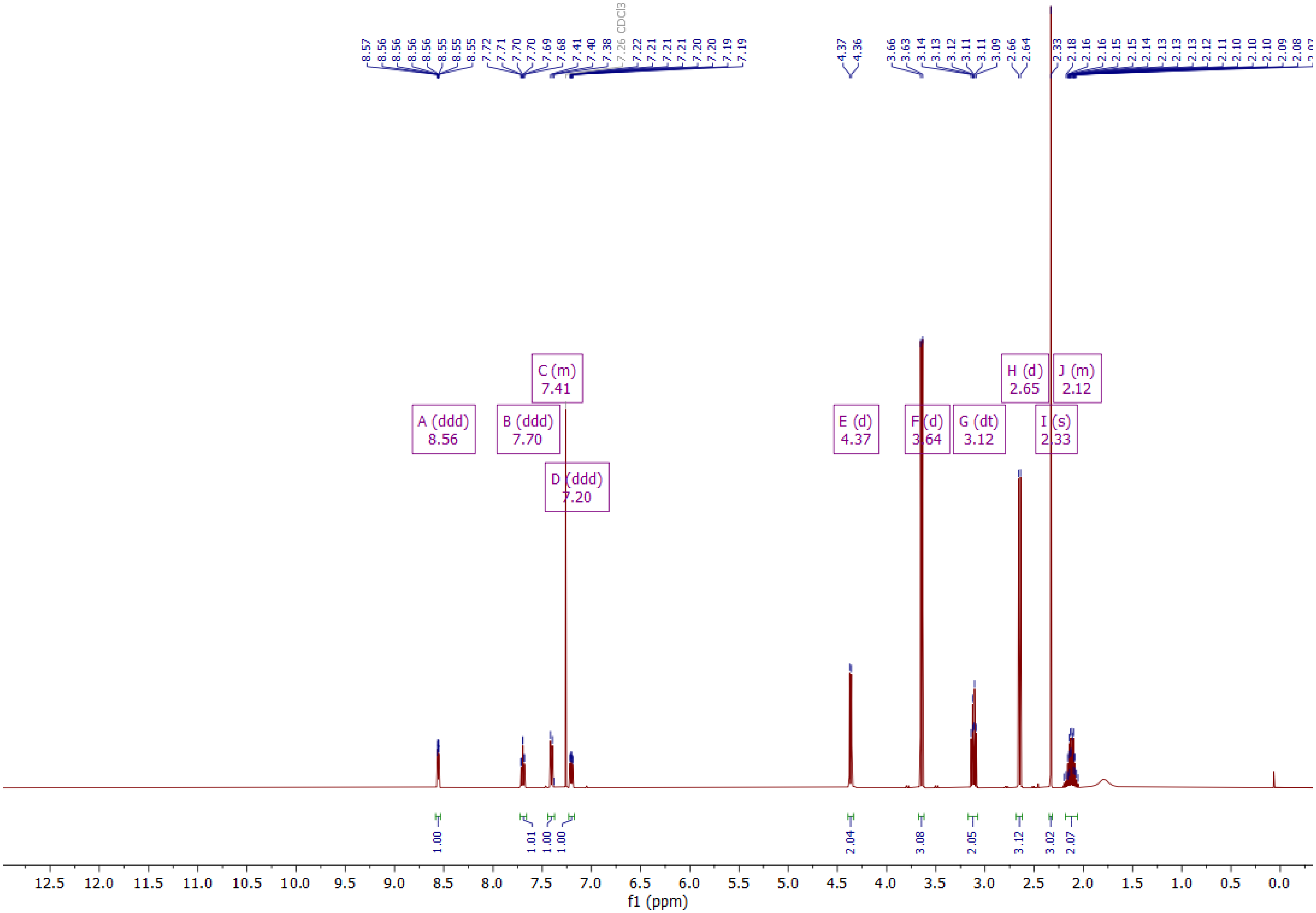

Figure S32. ^{1}H NMR spectrum of **1b**.

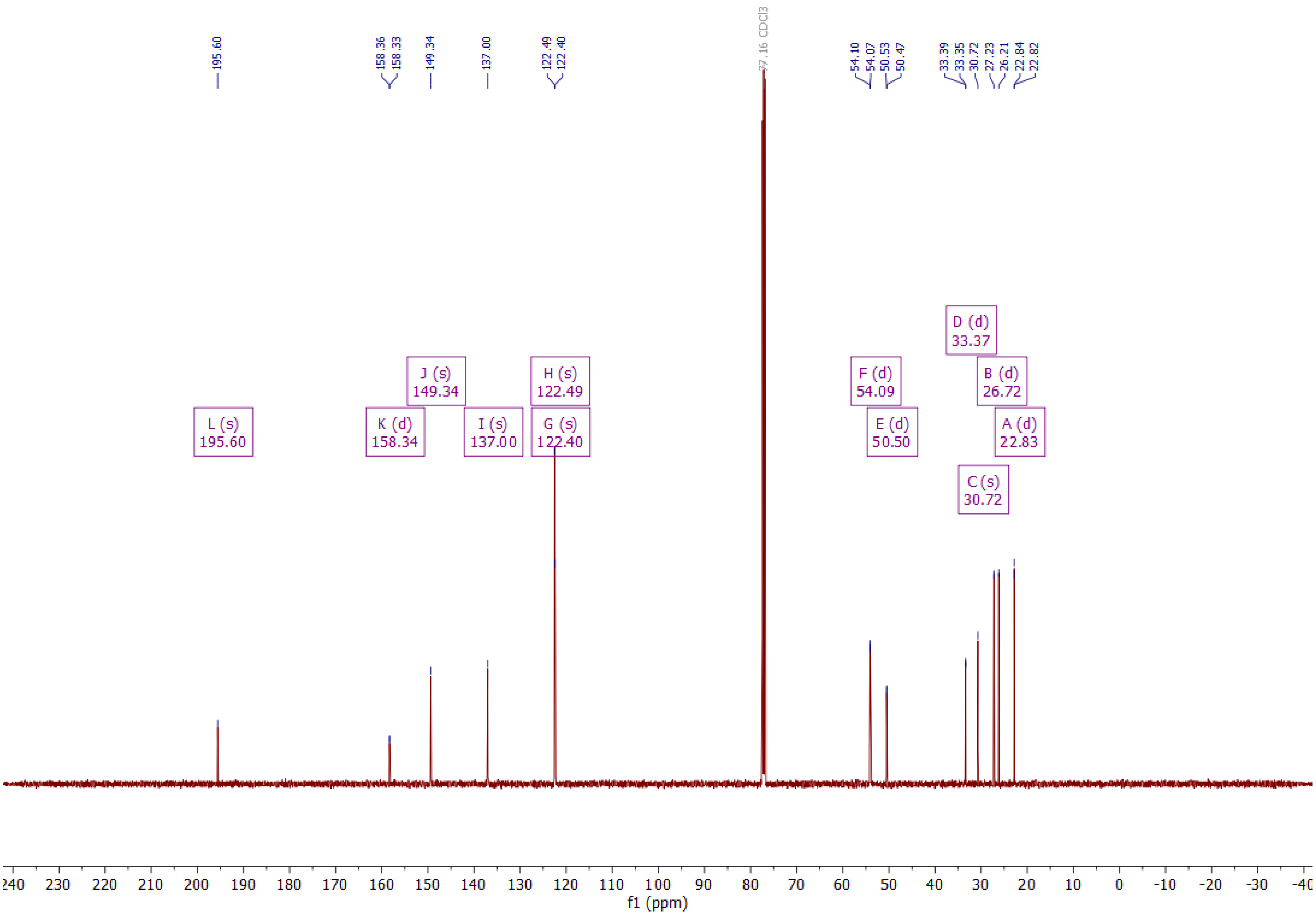

Figure S33. ^{13}C NMR spectrum of **1b**.

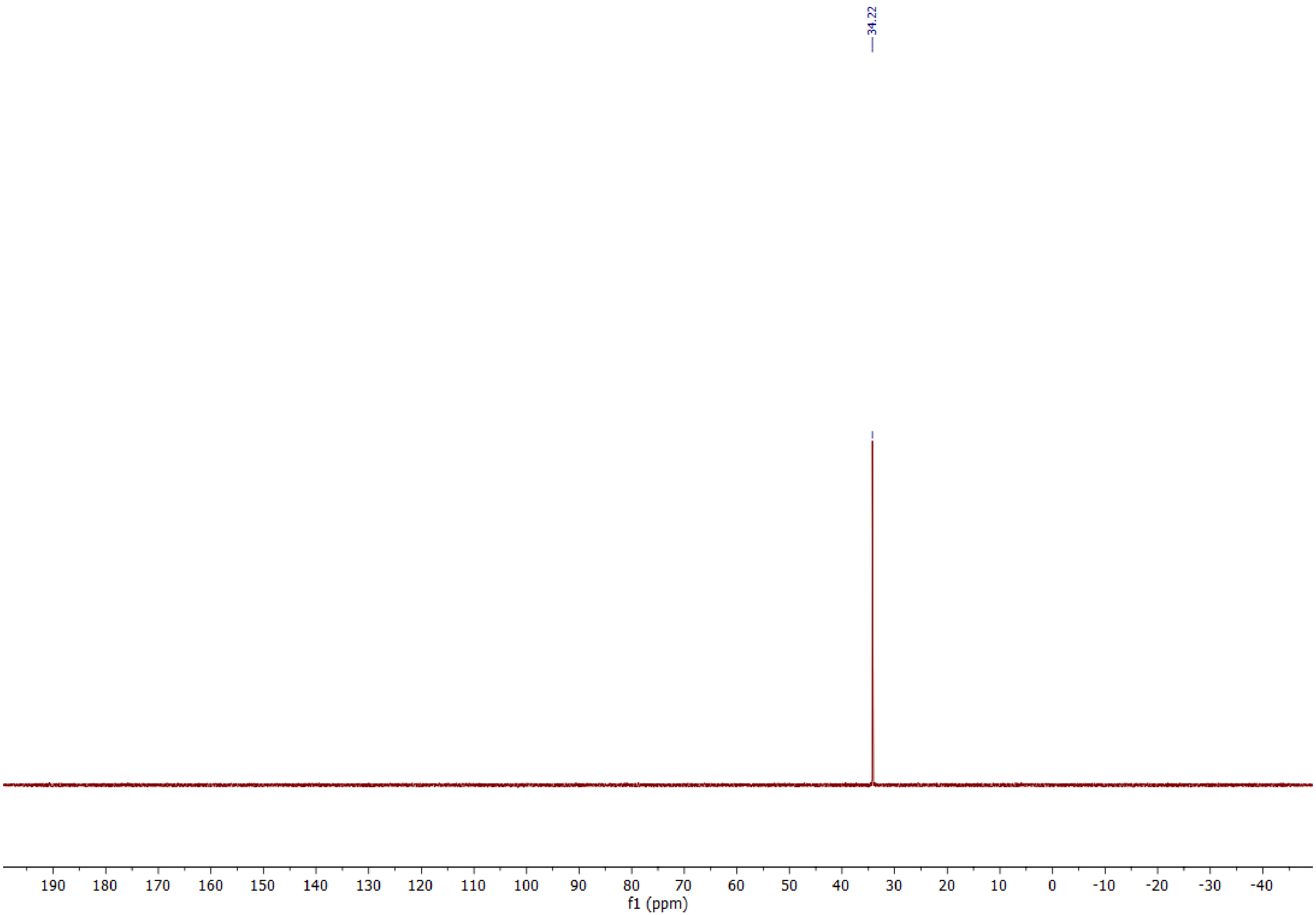

Figure S34. ^{31}P NMR spectrum of **1b**.

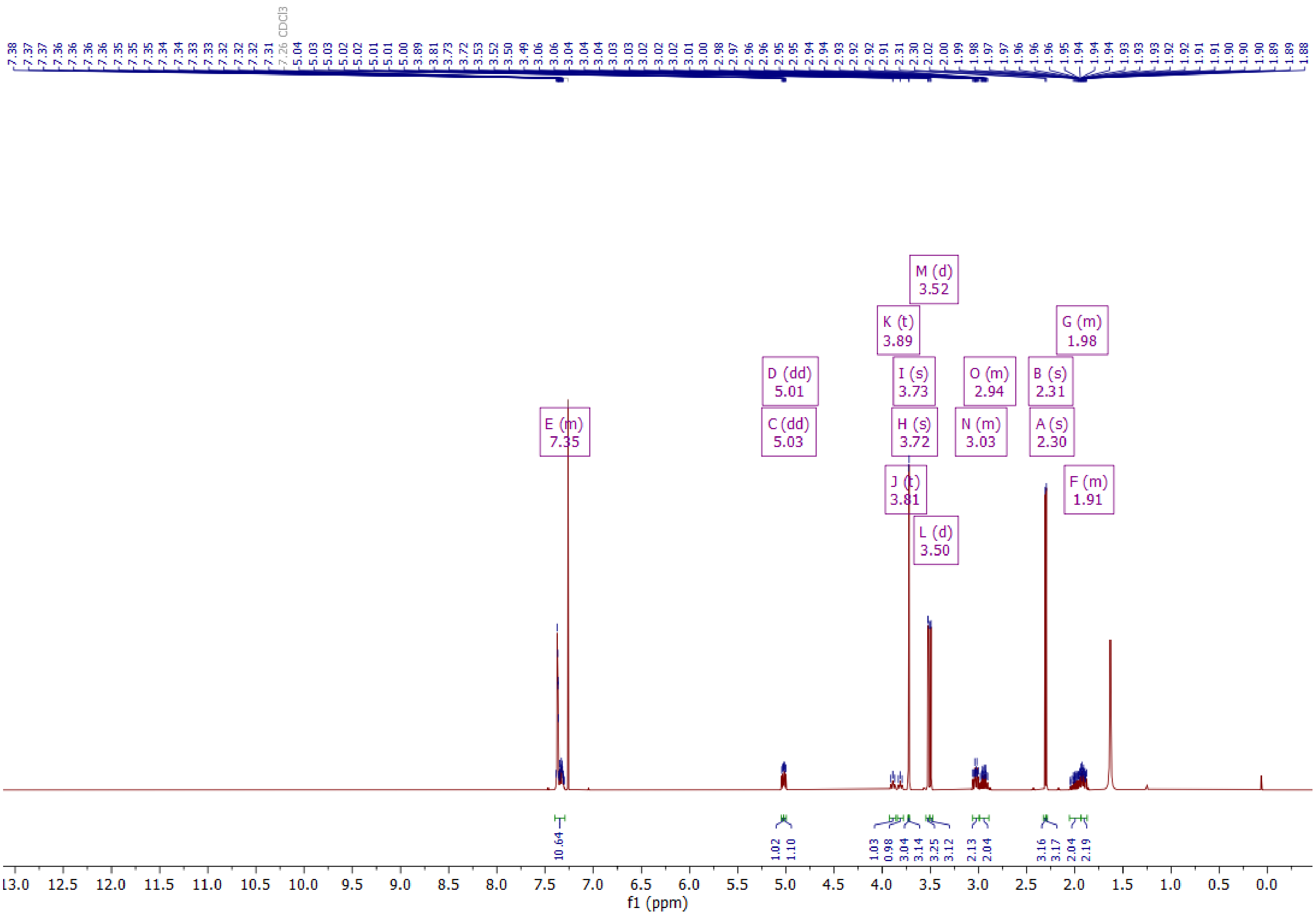

Figure S35. ^{1}H NMR spectrum of **1c**.

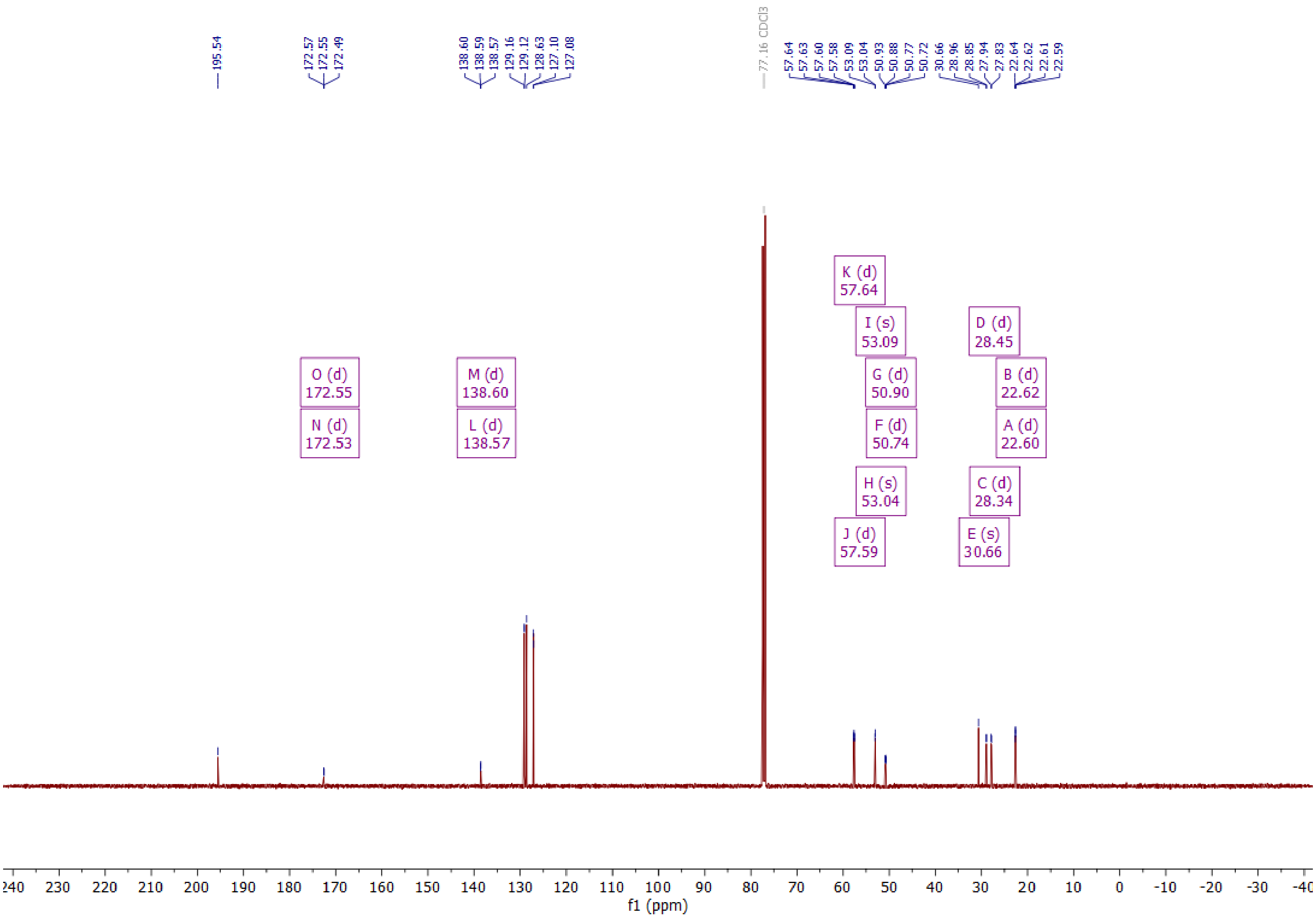

Figure S36. ^{13}C NMR spectrum of **1c**.

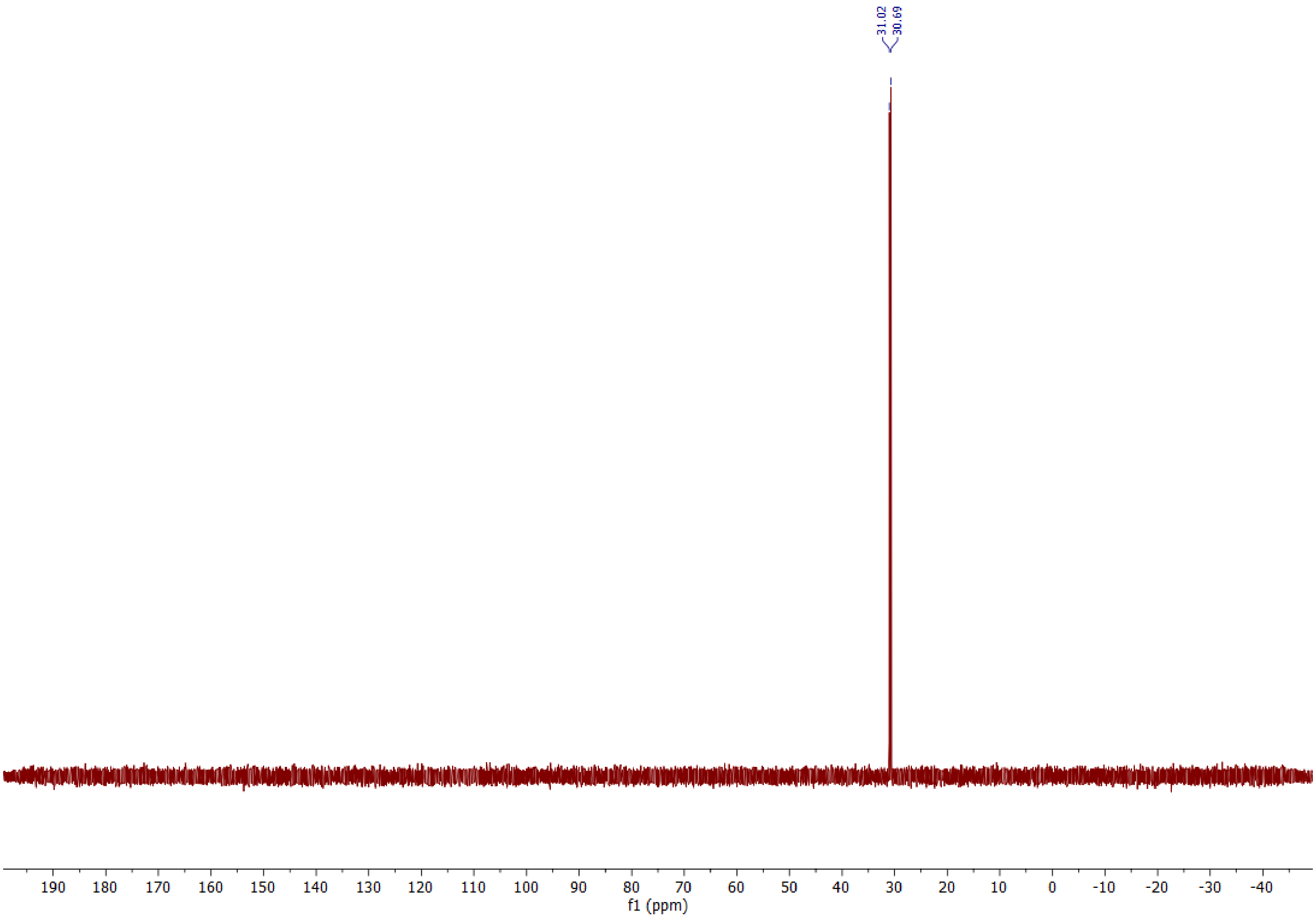

Figure S37. ^{31}P NMR spectrum of **1c**.

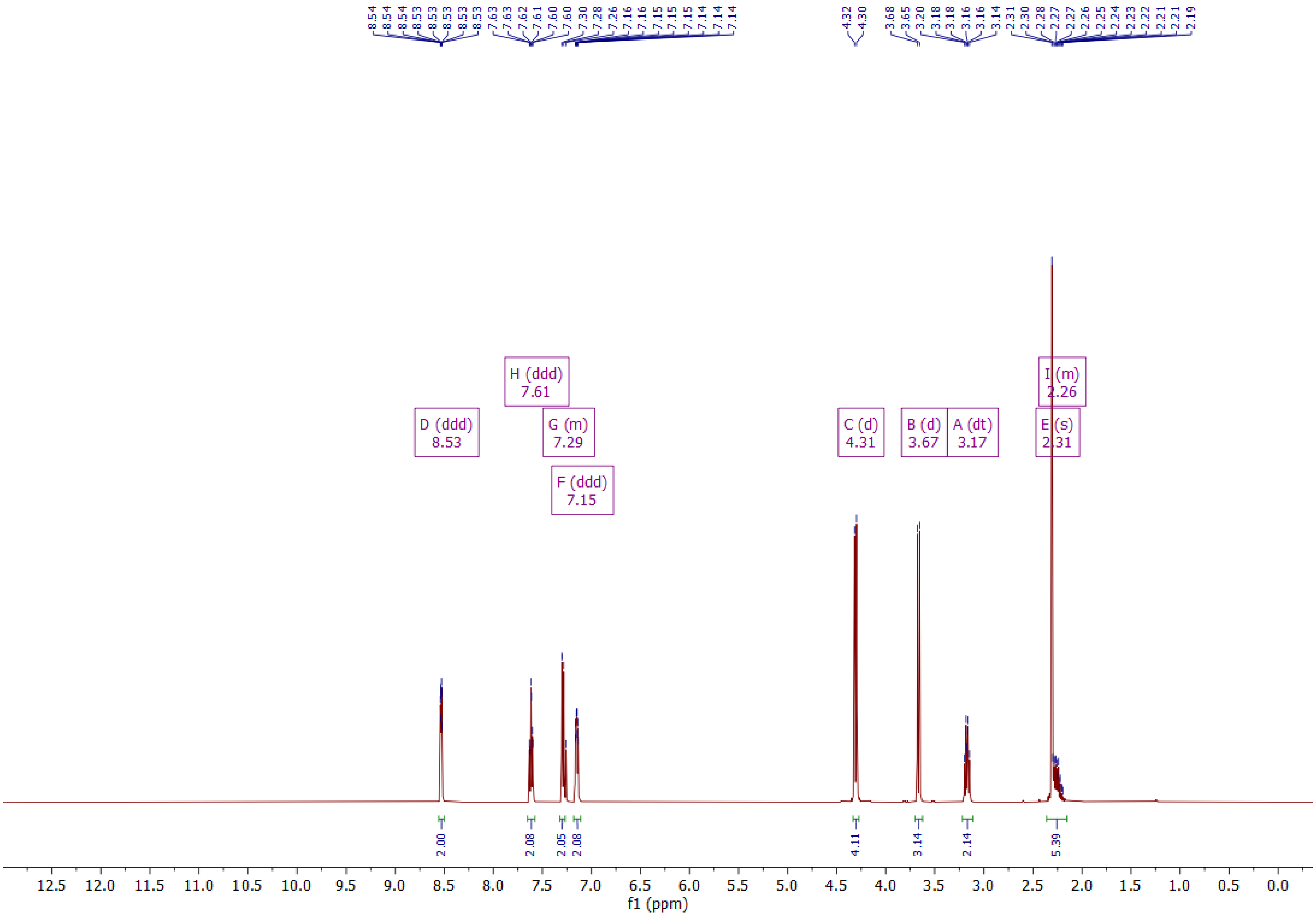

Figure S38. ^{1}H NMR spectrum of **1d**.

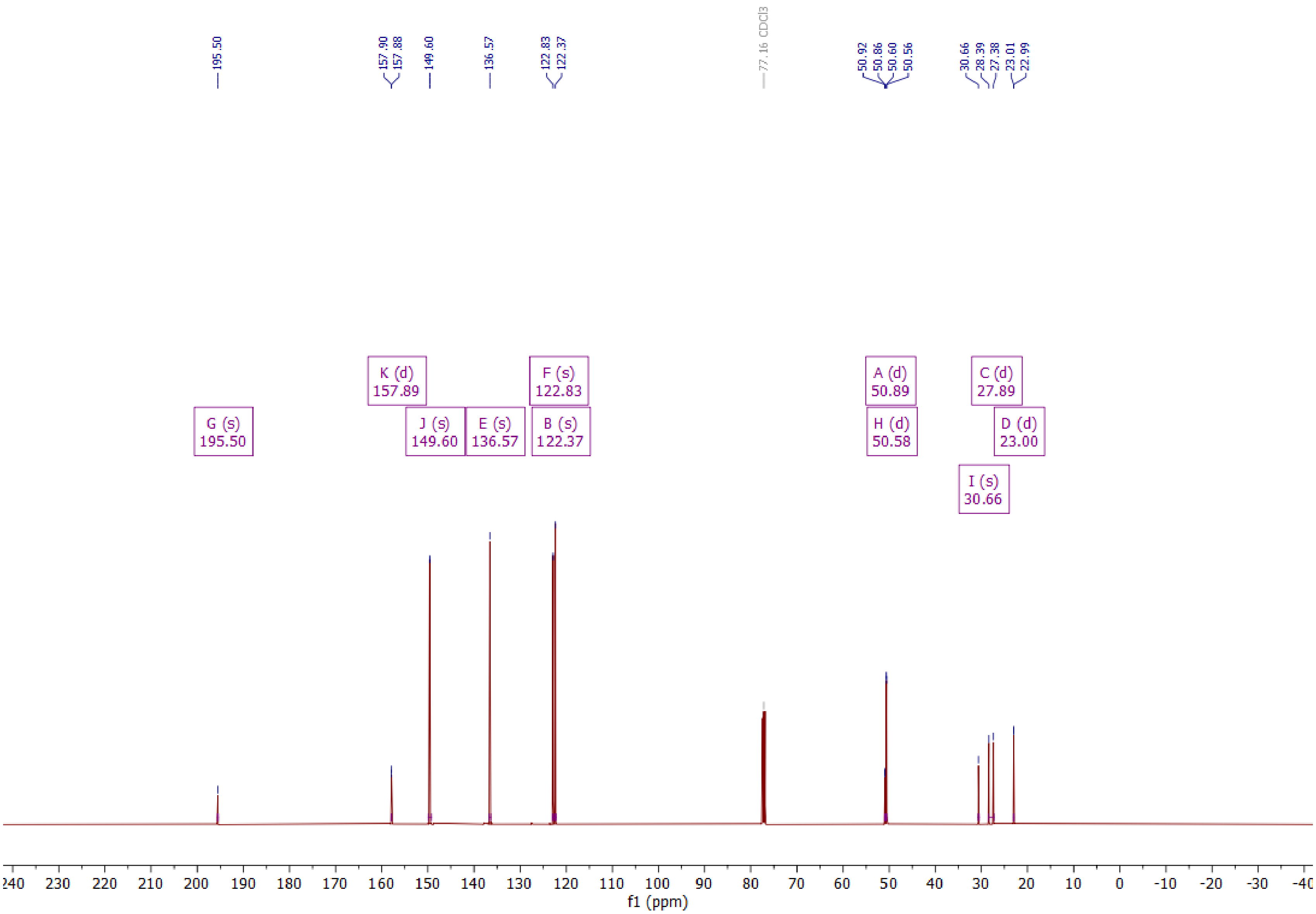

Figure S39. ^{13}C NMR spectrum of **1d**.

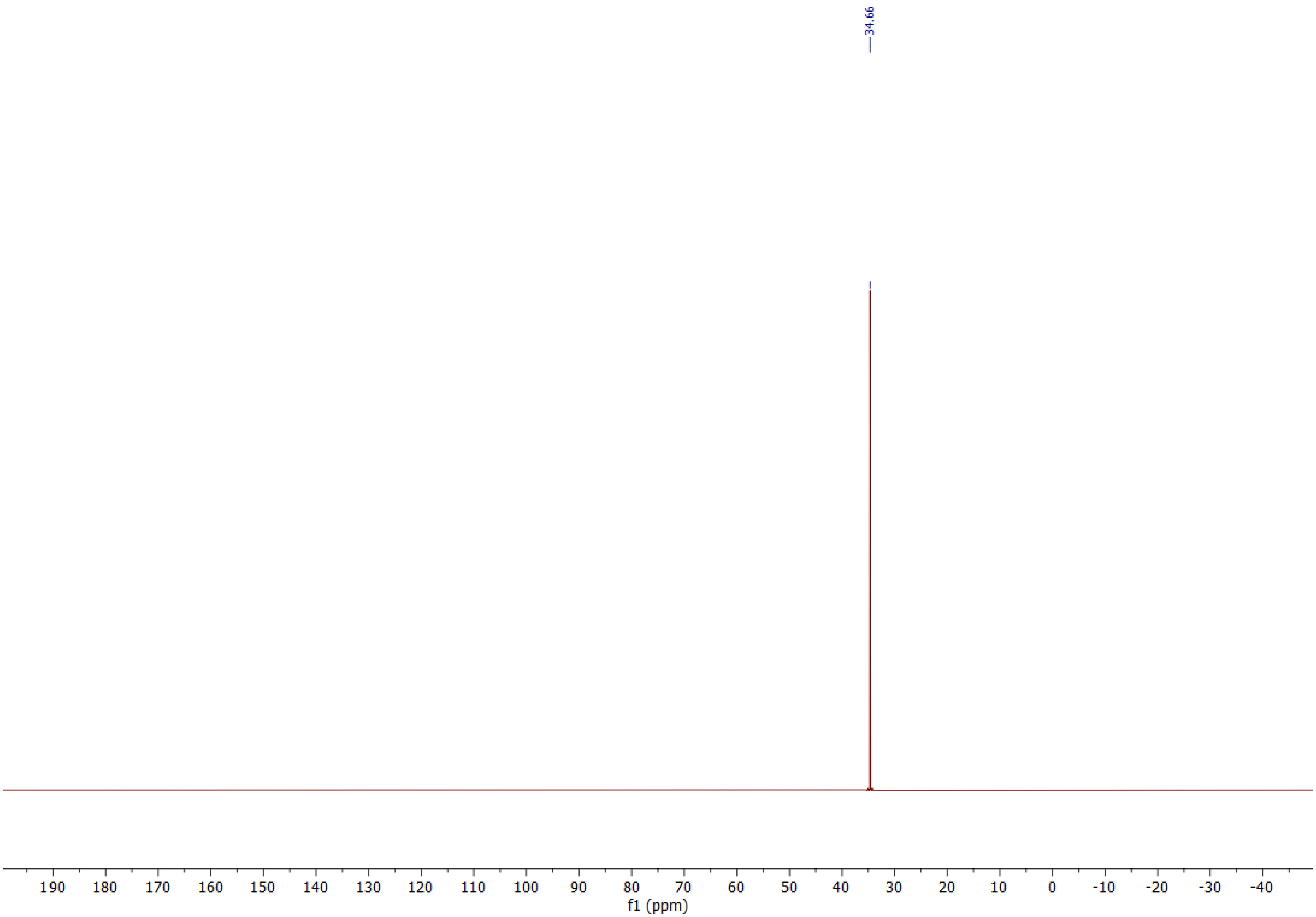

Figure S40. ^{31}P NMR spectrum of **1d**.

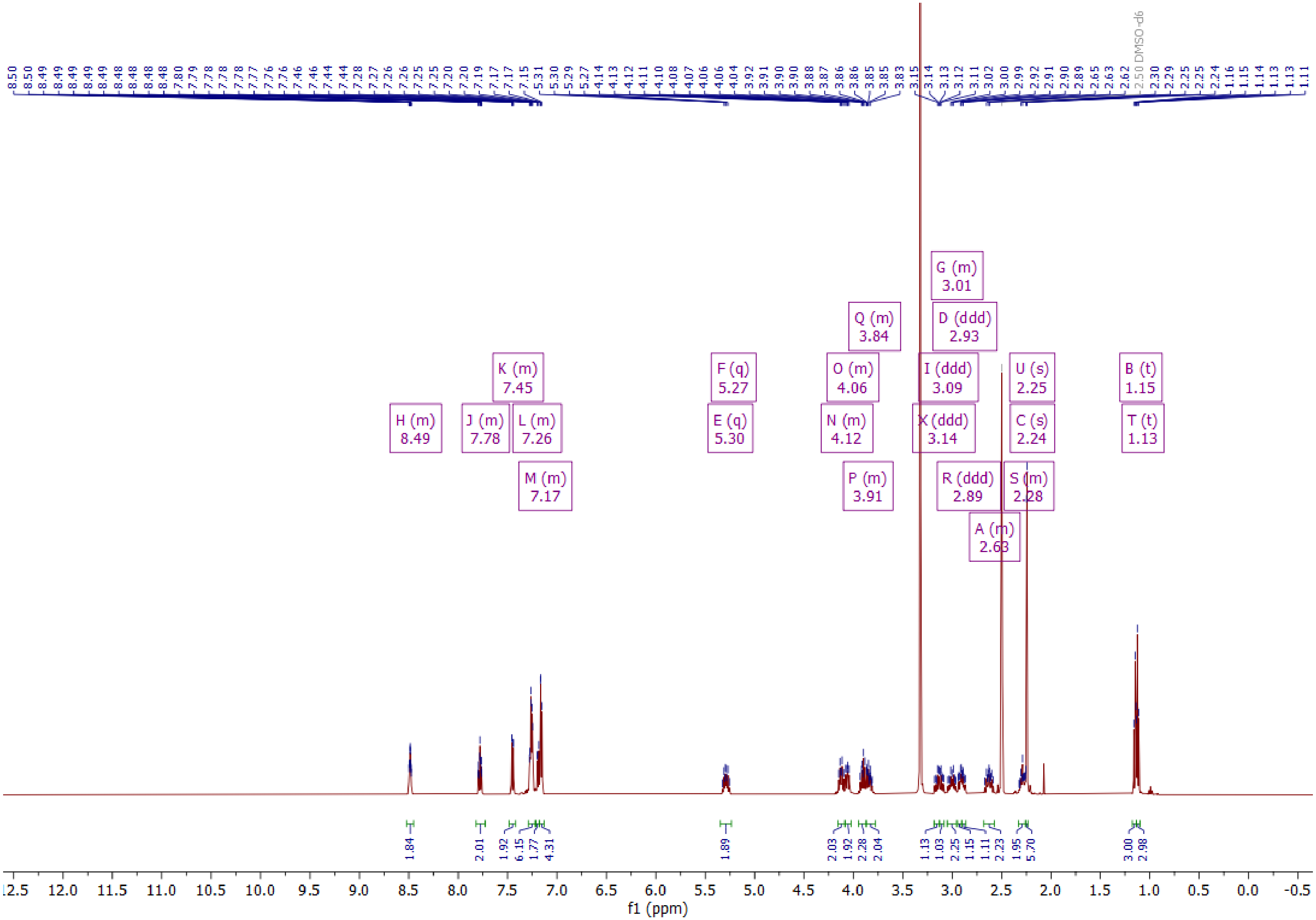

Figure S41. ^{1}H NMR spectrum of **1e**.

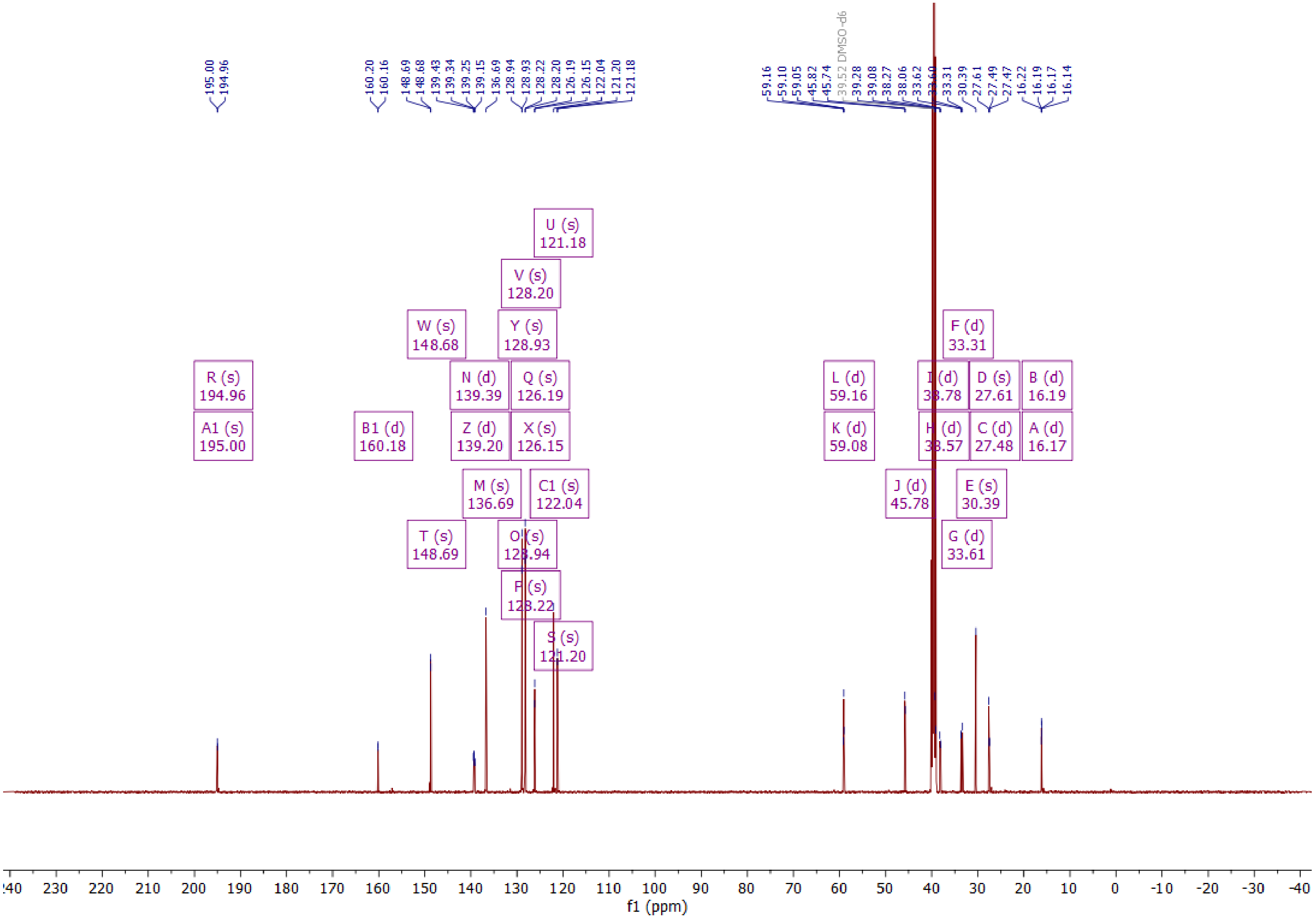

Figure S42. ^{13}C NMR spectrum of **1e**.

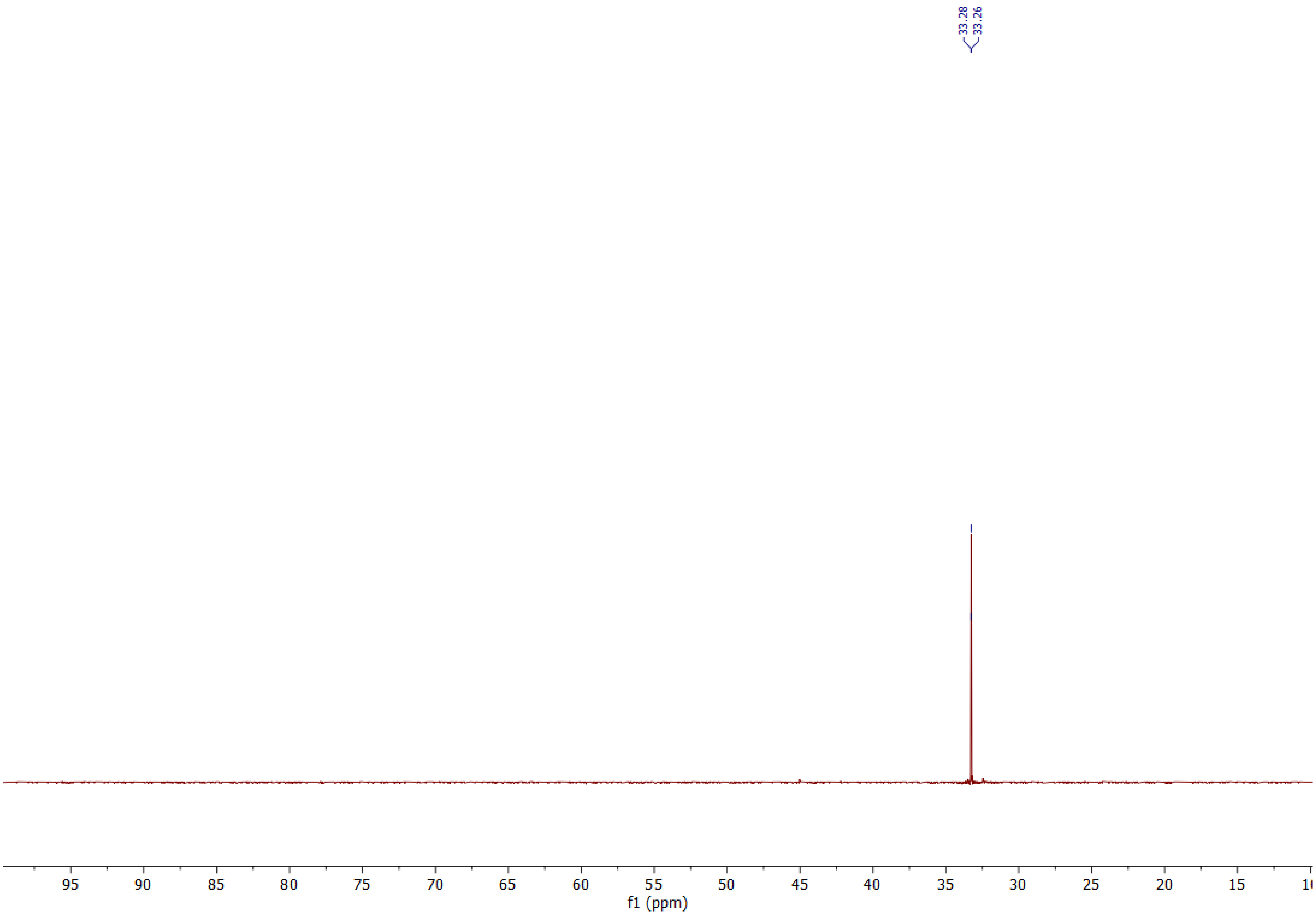

Figure S43. ^{31}P NMR spectrum of **1e**.

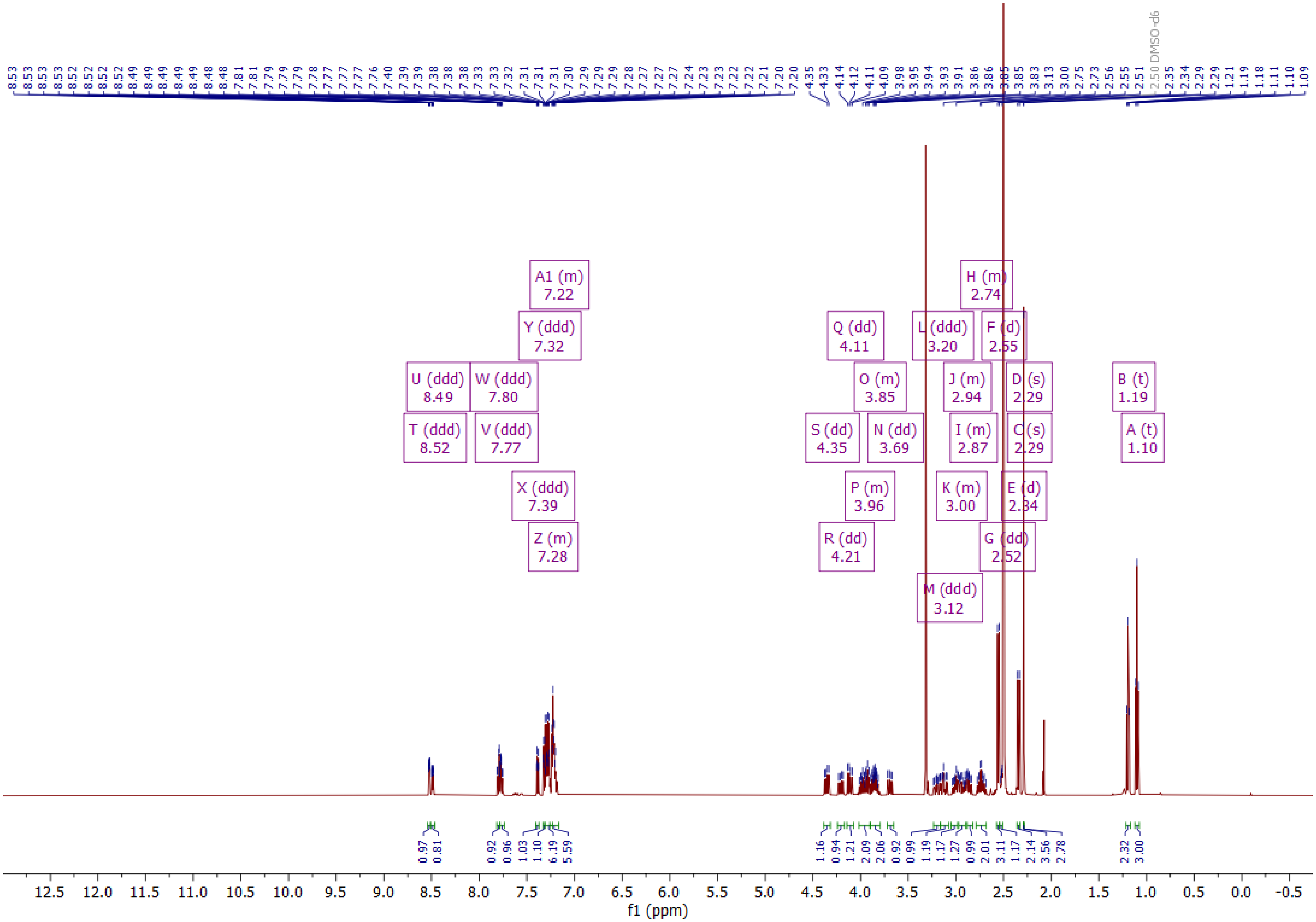

Figure S44. ^{1}H NMR spectrum of **1f**.

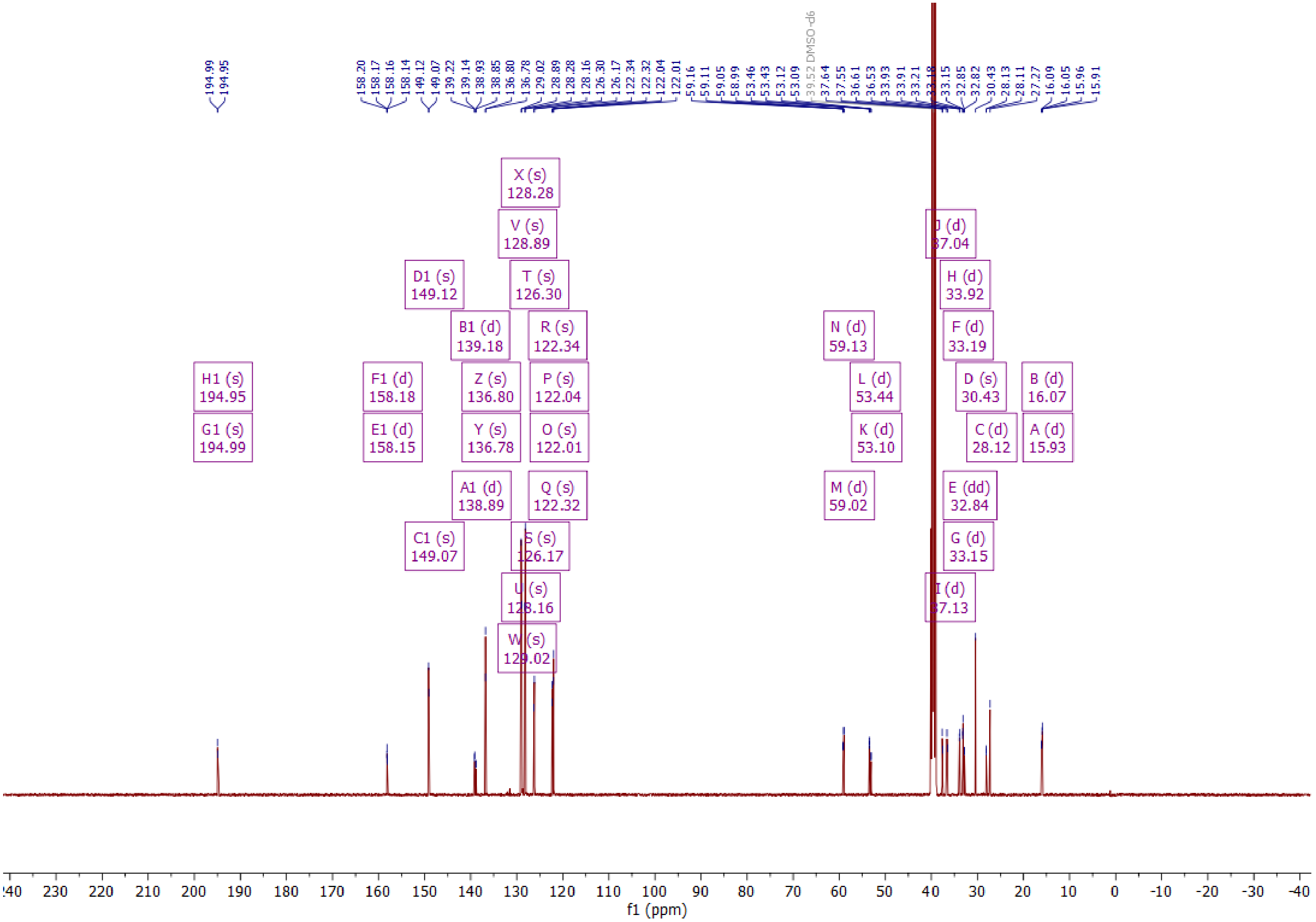

Figure S45. ^{13}C NMR spectrum of **1f**.

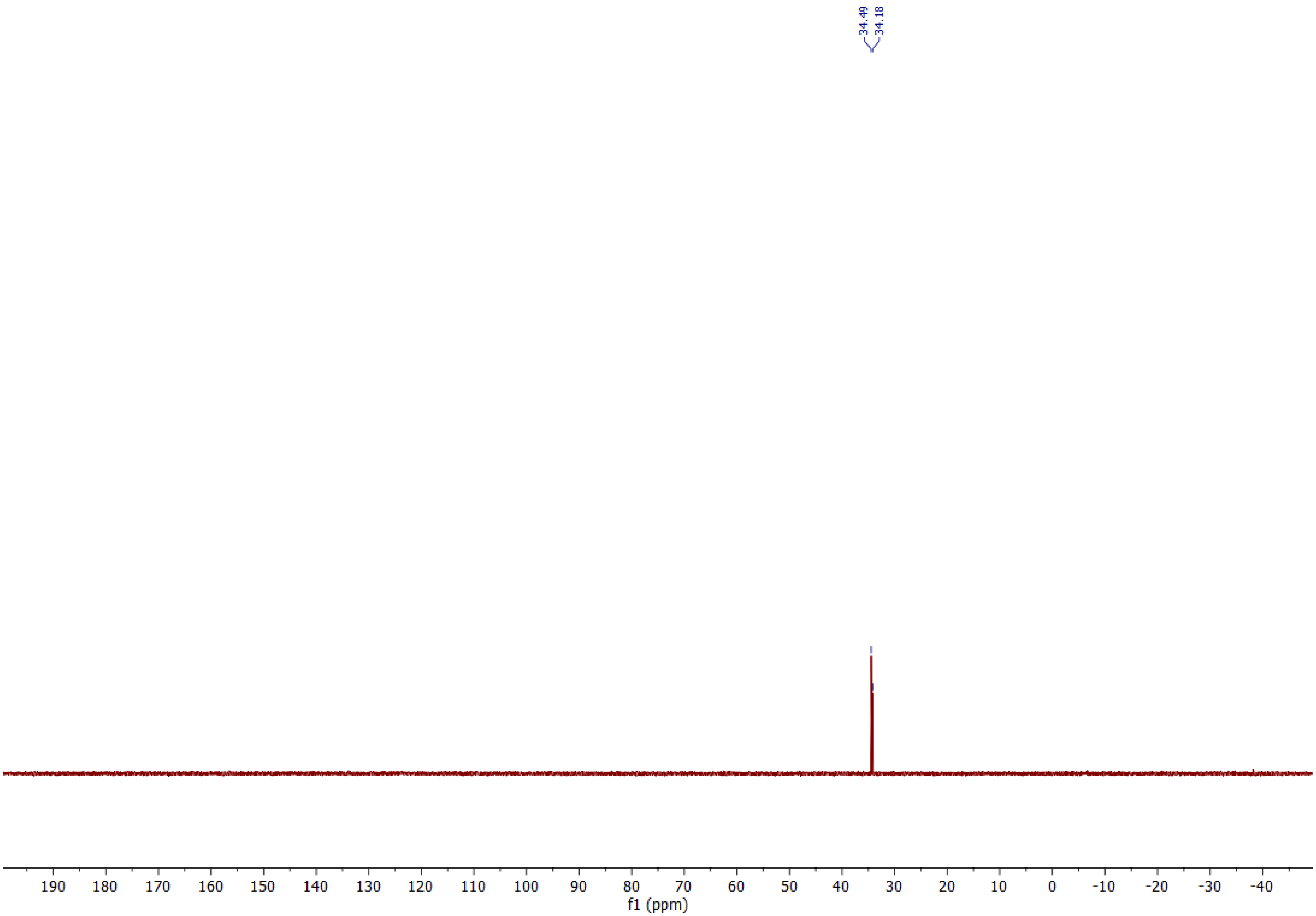

Figure S46. ^{31}P NMR spectrum of **1f**.

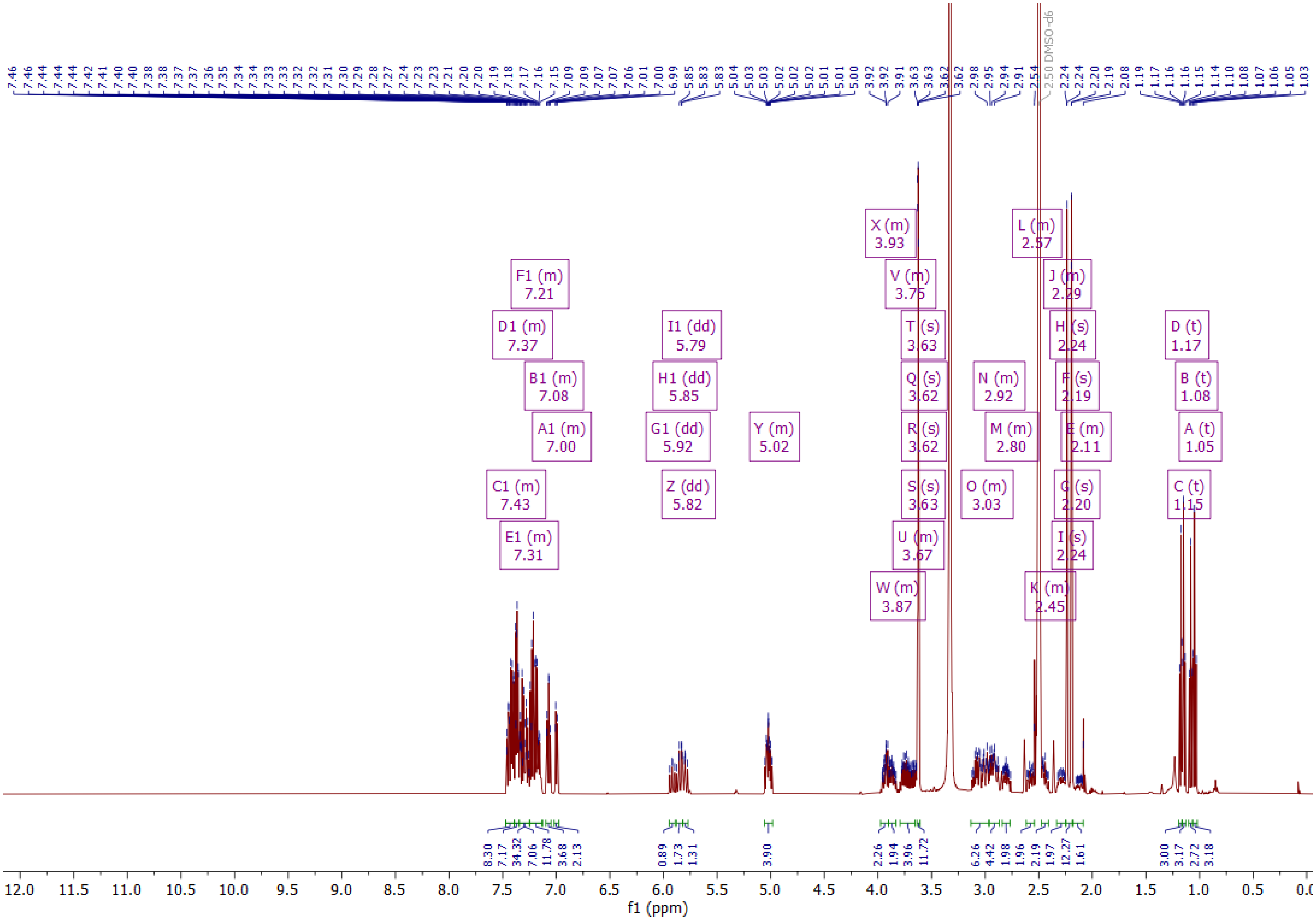

Figure S47. ^{1}H NMR spectrum of **1g**.

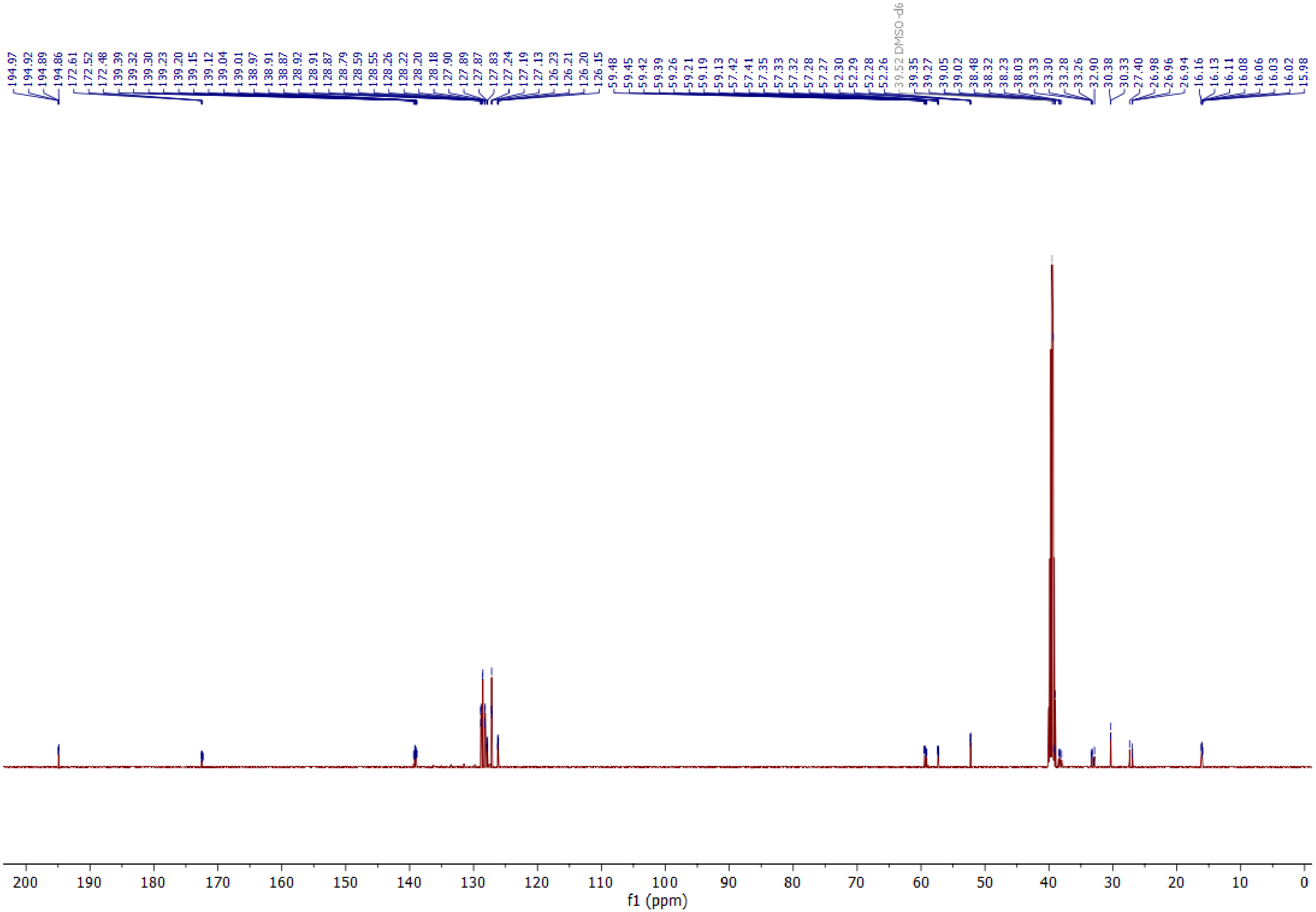

Figure S48. ^{13}C NMR spectrum of **1g**.

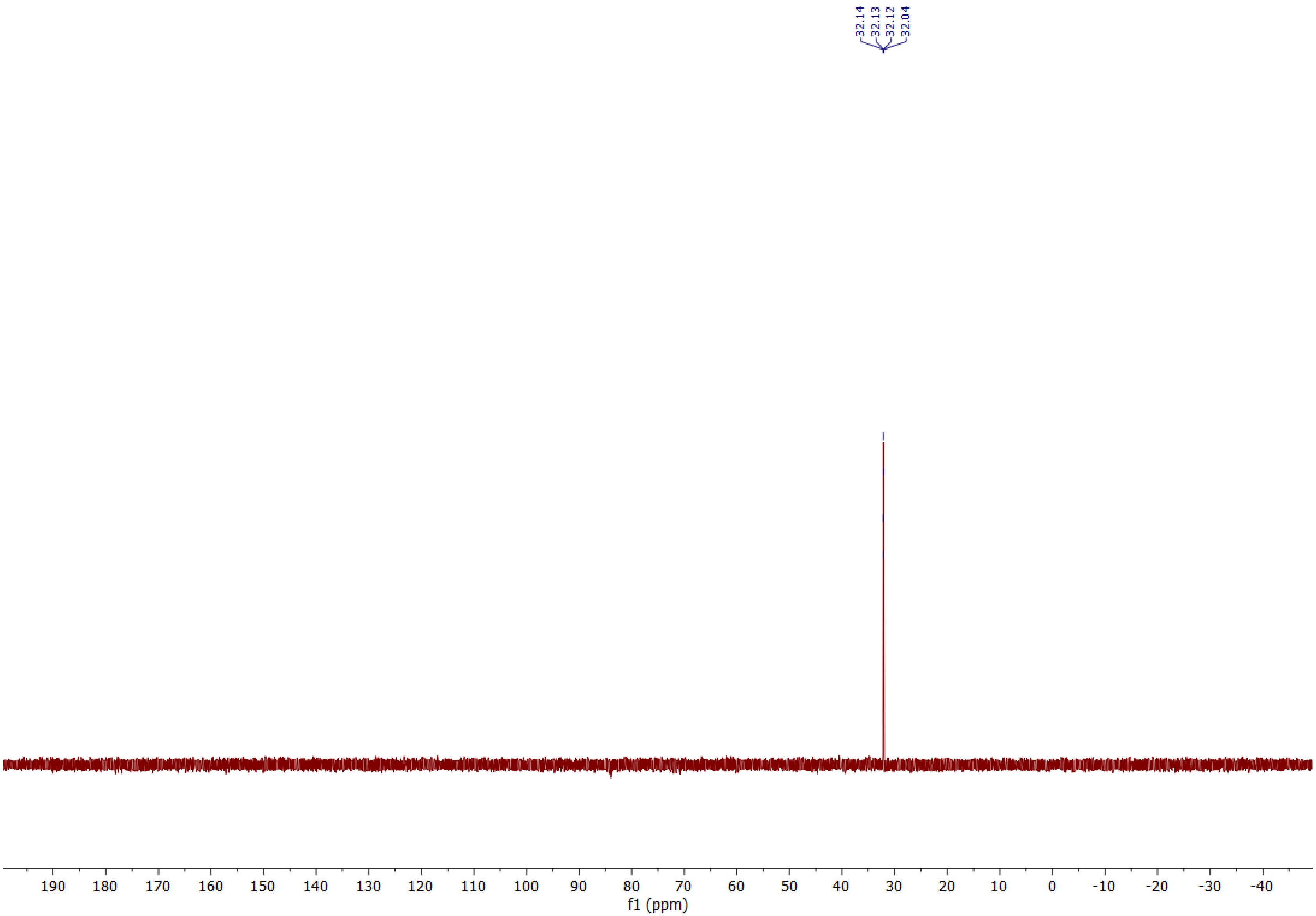

Figure S49. ^{31}P NMR spectrum of **1g**.

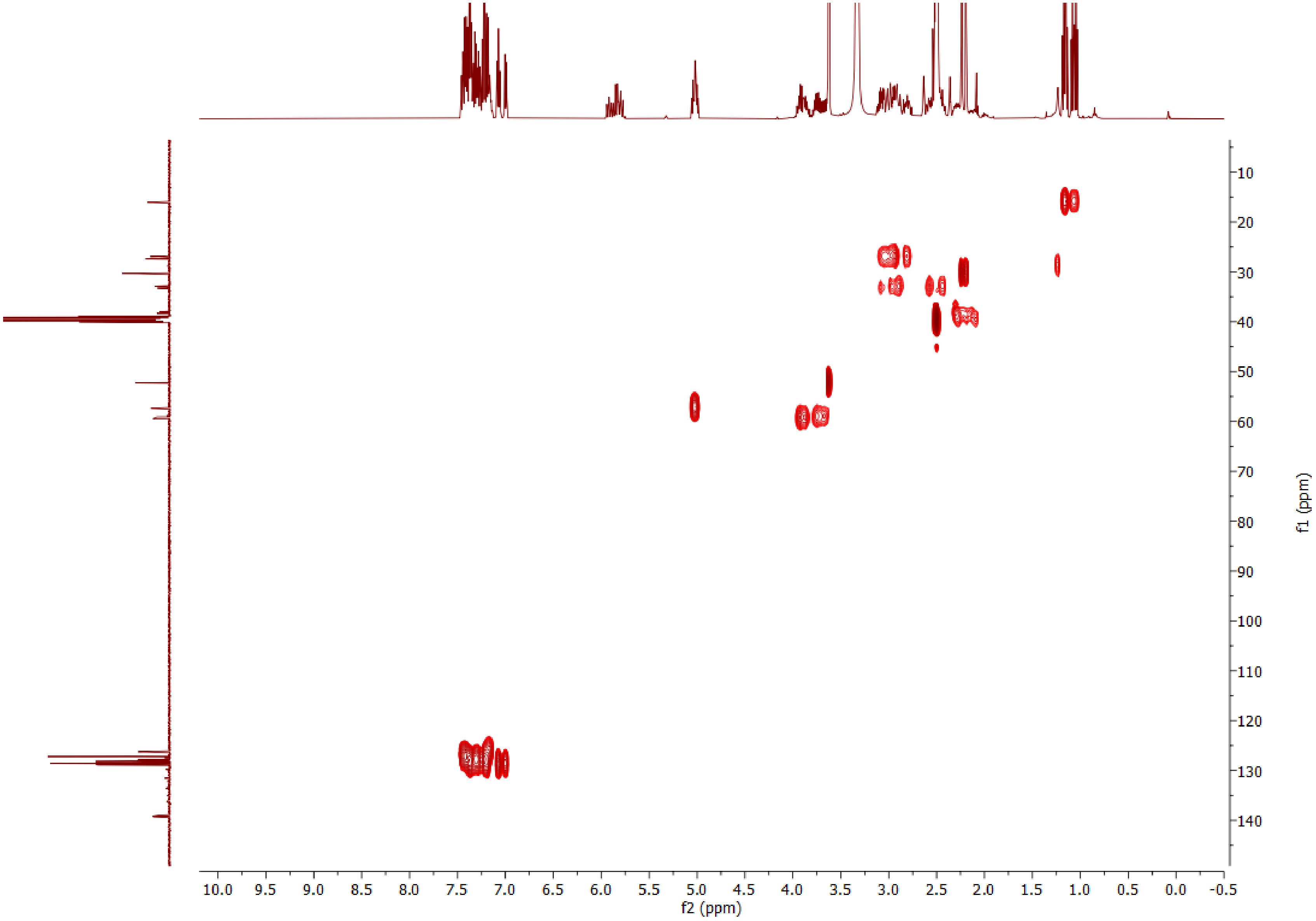

Figure S50. ^{1}H-^{13}C HSQC NMR spectrum of **1g**.

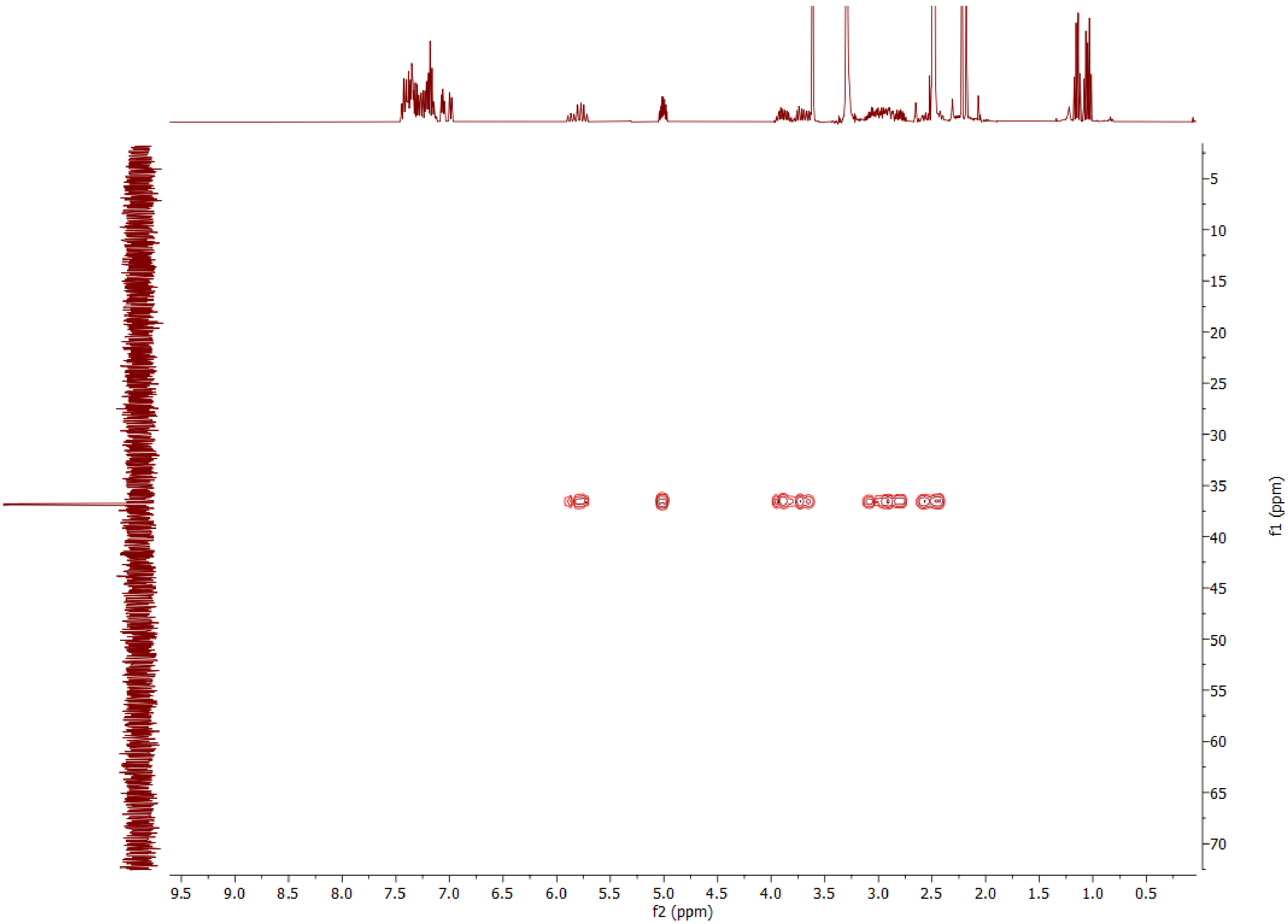

Figure S51. ^{1}H-^{31}P HMBC NMR spectrum of **1g**.

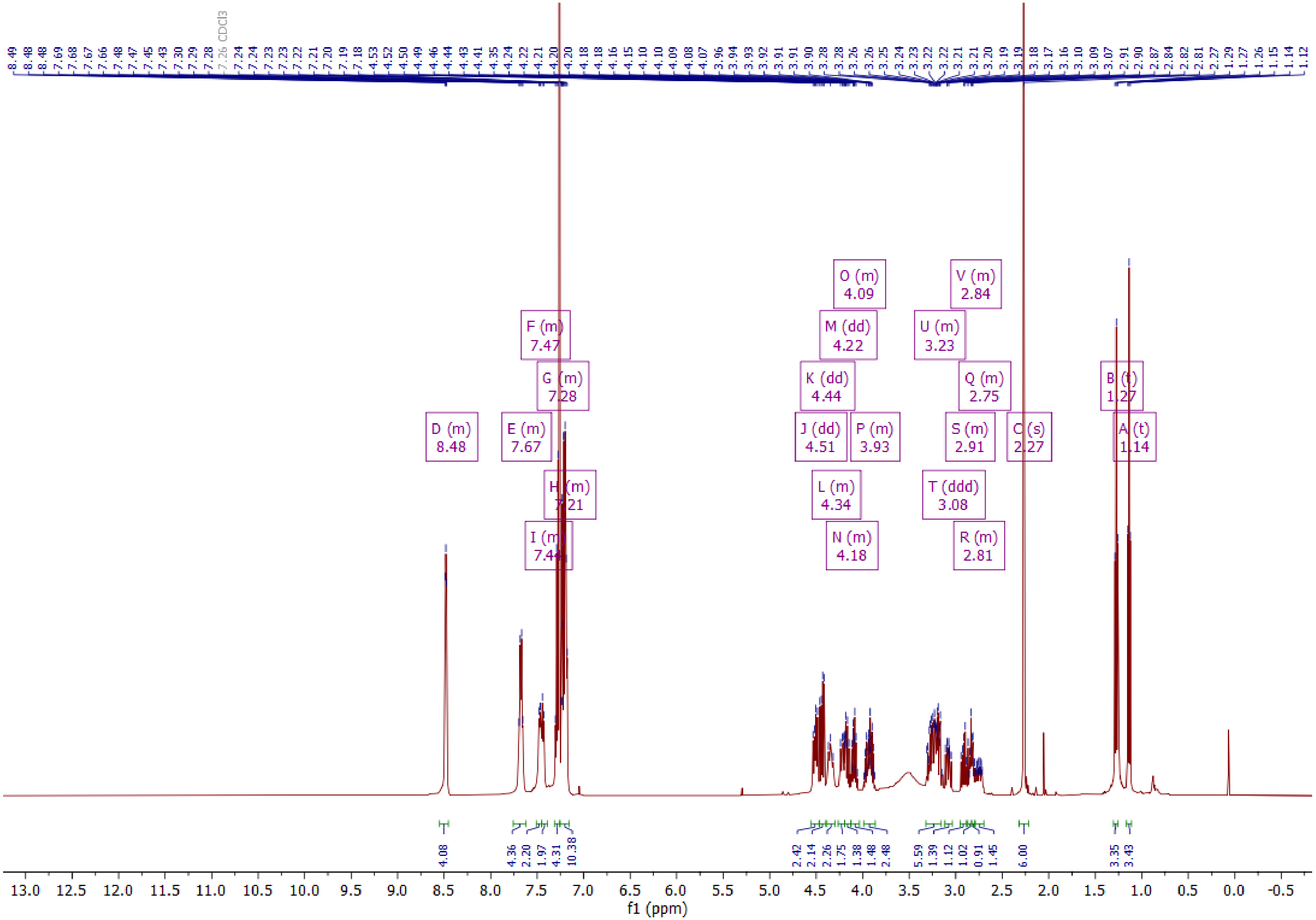

Figure S52. ^{1}H NMR spectrum of **1h**.

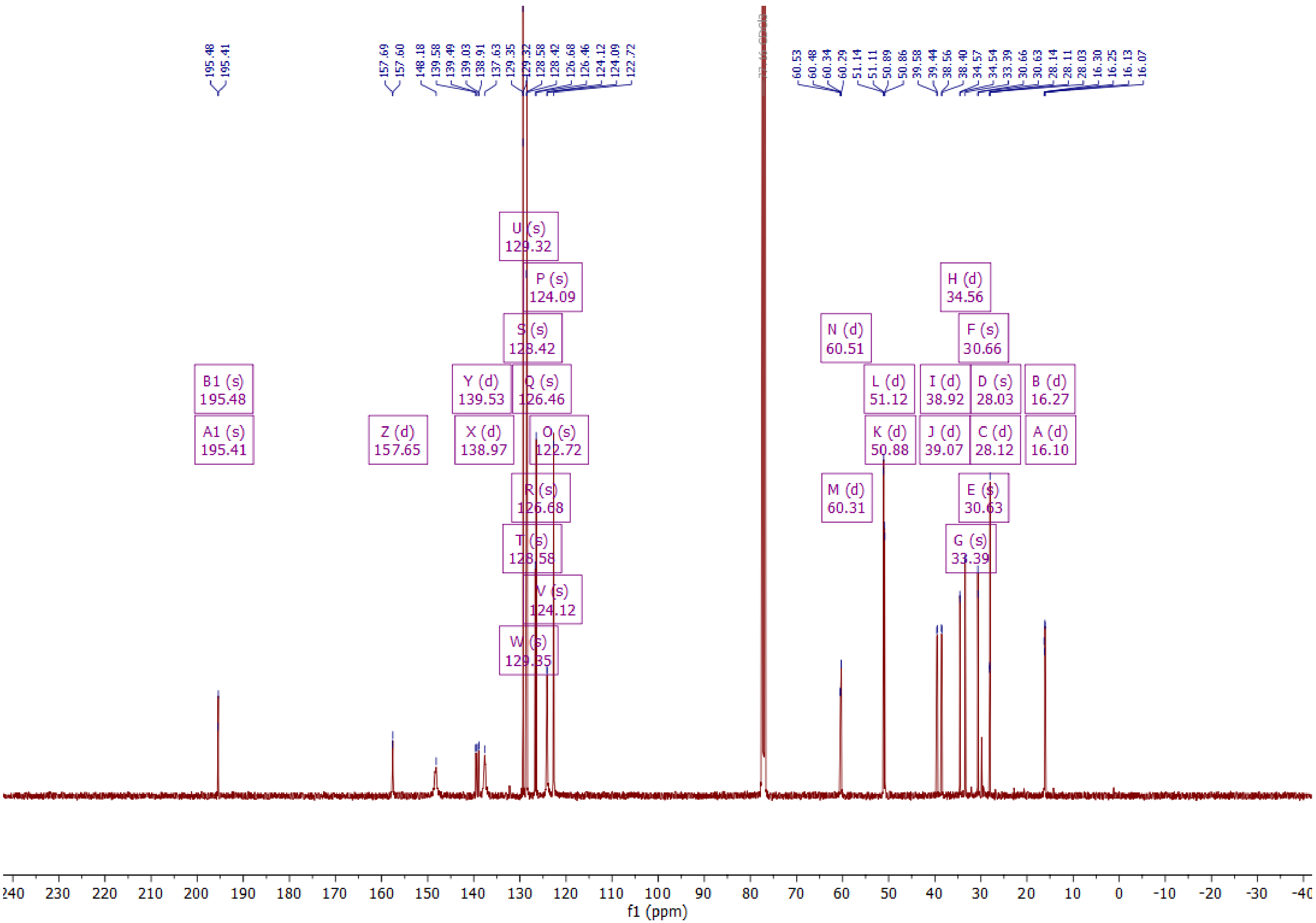

Figure S53. ^{13}C NMR spectrum of **1h**.

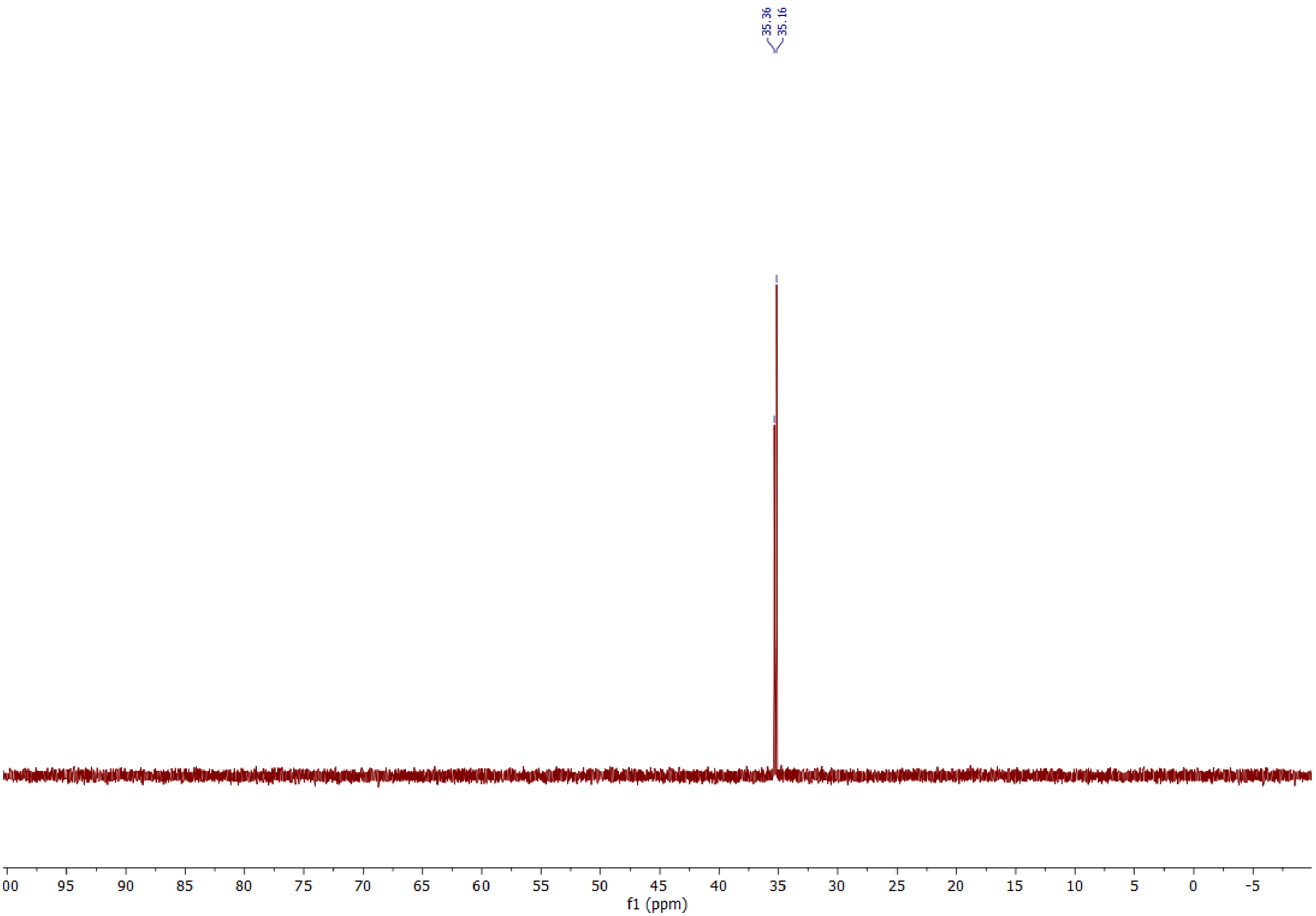

Figure S54. ^{31}P NMR spectrum of **1h**.
